# Chiral Vicinal Diamines Derived from Mefloquine

**DOI:** 10.1021/acs.joc.1c01316

**Published:** 2021-07-27

**Authors:** Dawid J. Kucharski, Rafał Kowalczyk, Przemysław J. Boratyński

**Affiliations:** † Department of Organic and Medicinal Chemistry, Wrocław University of Technology, Wyb. Wyspiańskiego 26, Wrocław 50370 Poland; ‡ Department of Bioorganic chemistry, Wrocław University of Technology, Wyb. Wyspiańskiego 26, Wrocław 50370 Poland

## Abstract

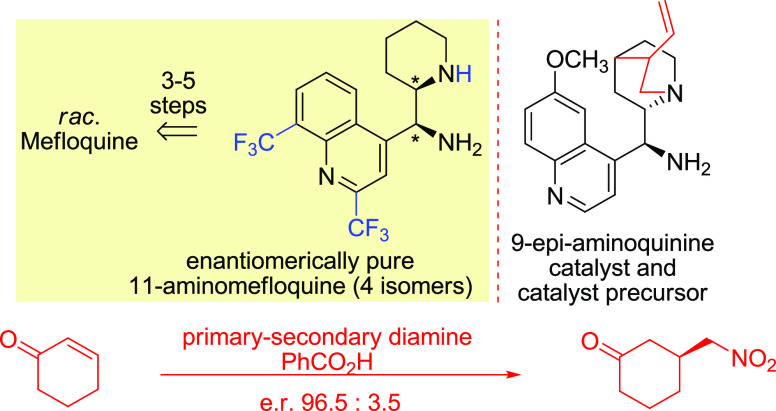

Novel 1,2-diamines based on the mefloquine scaffold prepared in enantiomerically pure forms resemble 9-amino-*Cinchona* alkaloids. Most effectively, 11-aminomefloquine with an *erythro* configuration was obtained by conversion of 11-alcohol into azide and hydrogenation. Alkylation of a secondary amine unit was needed to arrive at diastereomeric *threo*-11-aminomefloquine and to introduce diversity. Most of the substitution reactions of the hydroxyl group to azido group proceeded with net retention of the configuration and involved actual aziridine or plausible aziridinium ion intermediates. Enantiomerically pure products were obtained by the resolution of either the initial mefloquine or one of the final products. The evaluation of the efficacy of the obtained vicinal diamines in enantioselective transformations proved that *erythro*-11-aminomefloquine is an effective catalyst in the asymmetric Michael addition of nitromethane to cyclohexanone (up to 96.5:3.5 er) surpassing *epi*-aminoquinine in terms of selectivity.

## Introduction

**Figure 1:**
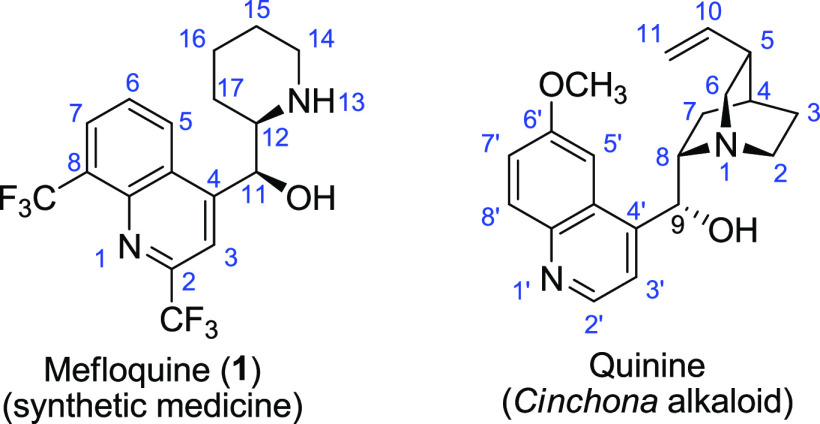
Comparison of the structures of mefloquine and quinine and traditional atom numbering.

Progress in organocatalytic approaches relies on the availability of effective chiral scaffolds and the means for their modification. A spectacular example is the application of *Cinchona* alkaloid derivatives: the most effective organocatalysts were formed by replacing the central hydroxyl group with an amine residue and some further specific modifications.^1,2^ The robust structure of the alkaloids, however, offers rather limited scope for further transformations and grater tuning of the catalyst. The available natural products, i.e., quinine and quinidine, are diastereomers, so different planned enantiomers of the target catalytic product require a separate method development. The pharmaceutical industry produces a close analogue of *Cinchona* alkaloids known as mefloquine, a drug against malaria (trade name Lariam). This compound shares some molecular characteristics of the alkaloids, while the major difference is the replacement of the quinuclidine bicyclic system with a piperidine ring and consequently a tertiary amine with a secondary one (Figure 1).

Mefloquine is sold as a racemate of *erythro* (*anti*) isomer; however, there exist a number of reported asymmetric syntheses^3^ and procedures for the separation^4^ or conversion of enantiomers.^5^ The dextrorotary compound was proven to be more effective against *Plasmodium* than its levorotary antipode. The latter is also much more toxic and prone to induce psychotic behavior.^6^ Mefloquine could one day become available as a single enantiomer as this would likely improve pharmacological properties and reduce side effects. Until now, these efforts have not been commercially successful.

Mefloquine derivatives are able to selectively interact with chiral entities.^7^ Therefore, these scaffolds could be considered for asymmetric catalytic purposes. A wide array of modifications at both reactive positions 11 and 13 could be envisaged to arrive at effective organocatalysts or metal ligands. In this paper, we develop syntheses of all stereoisomers of 11-aminomefloquine by the substitution of the central hydroxyl group with an amino group. Such modification of a similar *Cinchona* scaffold was vital to the progress of organocatalysis.

## Results and Discussion

**Scheme 1:**
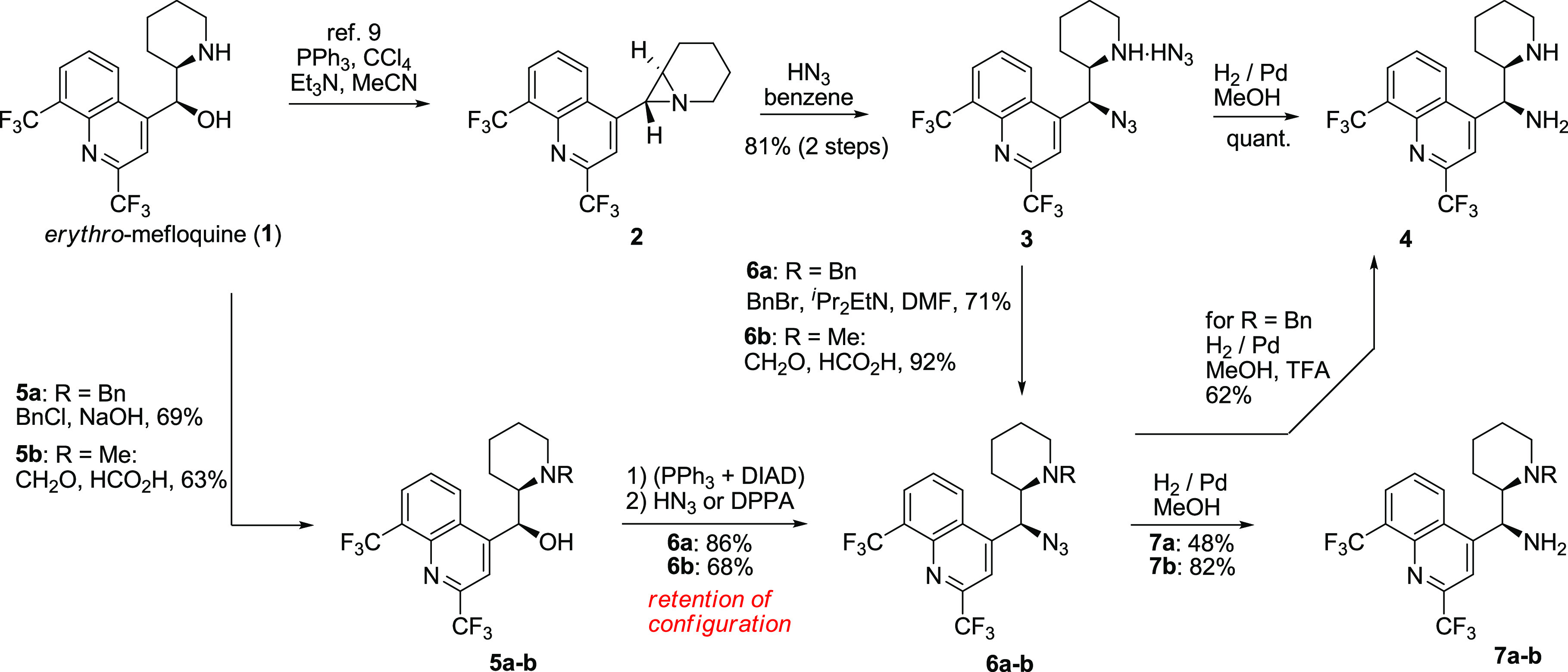
Transformations of Mefloquine (**1**) to *erythro-*11-Aminomefloquine (**4**)

The reactions of *Cinchona* alkaloids at their central 9 position are sometimes known to proceed with unpredictable stereochemical outcomes, which could later be explained by various effects, such as neighboring group participation, chelation, and formation of hydrophilic cavities. Due to stereocovergence in the substitution reactions with carbon and oxygen nucleophiles, some isomers were more easily obtainable than others.^2,8^ The displacement with an azide nucleophile either in the Mitsunobu reaction or the substitution of alkaloid 9-methanesulfonates always proceeded according to the straightforward S_N_2 mechanism. An attempted exploitation of the corresponding reactivity of mefloquine at position 11 resulted in a rather surprising stereochemistry (Scheme 1).

### *erythro*-1,2-Diamine

**Scheme 2:**
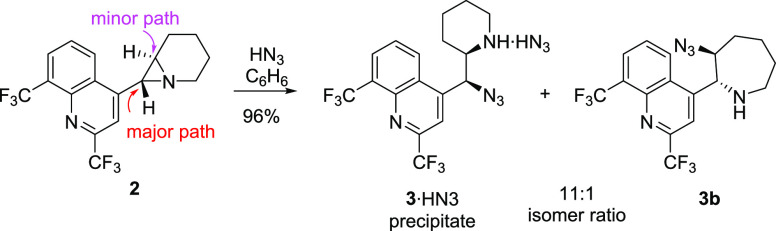
Nucleophilic Ring Opening of Pure Aziridine **2**

A direct implementation of the *Cinchona* alkaloid transformation brought us to the nucleophilic displacement in the Mitsunobu reaction with an azide source followed by Staudinger reduction. The reaction led to the retention of the configuration rather than the expected inversion and gave product **4** in low yield (11–15%). We then turned to producing aziridinie **2** in the Appel reaction following a reported procedure.^9^ The aziridine **2** is moderately stable, though it undergoes gradual decomposition even in the solid state over the period of months. Efforts to isolate pure aziridine generally lead to moderate yields. The aziridine ring was efficiently opened with hydrazoic acid to give azidoamine **3** (Scheme 2). When only partially purified aziridine **2** was subjected to the same process, the corresponding azide **3** was formed very efficiently over two steps (81%). Most of the 11-azide **3** precipitates from the reaction medium in very high purity as a hydrazoic acid salt (75%), as proven by elemental analysis. The investigation of the supernatant composition revealed the formation of 9% of an isomer **3b** possessing a 7-membered ring (Scheme 2). The formation of aziridine occurs via an S_N_2 mechanism,^9^ and the ring-opening also involves S_N_2 reaction resulting in the net retention of the configuration in the azidoamine **3**.

The hydrogenation of 11-azidomefloquine salt (**3**·HN_3_) delivered quantitatively the corresponding *erythro* vicinal primary–secondary diamine **4**. The entire sequence from **1** to **4** can be achieved in up to a 75% yield using only paper filtration as the means of purification. Alternative Staudinger reduction resulted in the formation of a triphenylphosphine adduct observed in the MS spectra which could not be effectively forced to hydrolyze.

**Scheme 3:**
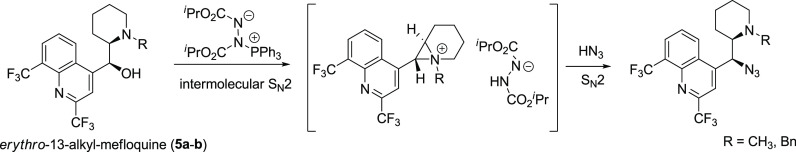
Plausible Course of Retentive Mitsunobu Reaction

In an attempt to produce a diastereomer of diamine **4** along with primary–tertiary diamine analogues, alkyl substituents were introduced at the piperidine nitrogen atom (Scheme 1). The benzyl group was chosen as transient protection since it could be removed under hydrogenation conditions. Mefloquine was benzylated under Schotten–Bauman conditions to give **5a**, while Eschweiler–Clarke methylation provided **5b**. Alkyl mefloquine derivatives **5a**,**b** were then subjected to the Mitsunobu reaction with an azide source. Good yields were achieved when triphenylphosphine was first reacted with azadicarboxylate to produce a zwitterionic adduct prior to the addition of a mixture of hydrazoic acid and **5a** or **5b**. An alternative sequence of the addition of reagents essentially failed to produce the required products. The 11-amines **7a** and **7b** were obtained by hydrogenation using palladium on charcoal in methanol. The benzyl group was removed when the hydrogenation was performed in a 5% TFA solution. Surprisingly, it was found that hydrogenation of both **3** and **6a** resulted in the formation of the same product **4**. Moreover, alkylation of azide **3** under acidic or mildly basic conditions resulted in *erythro*-products **6a**,**b** identical to those produced by the alkylation–Mitsunobu sequence (Scheme 1). These results are indicative of **5a**,**b** reacting with the net retention of the configuration under the Mitsunobu conditions. This phenomenon could be explained by transient aziridinium ion formation followed by ring opening^10^ (Scheme 3). Lack of anchimeric assistance impedes formation of 11-azides from 13-acyl-mefloquine under Mitsunobu conditions.

### *threo*-1,2-Diamine

**Scheme 4:**
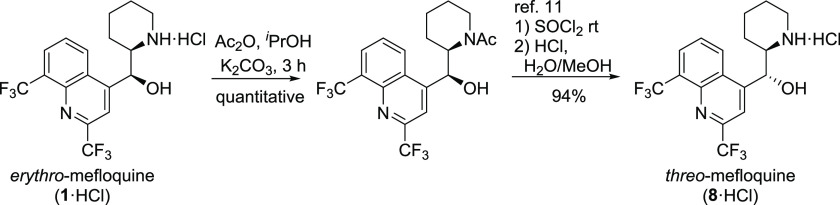
Shortened Method of Inversion of *erythro*-Mefloquine

The catalytically vital *epi*-*Cinchona* alkaloid series is sterochemically analogous to mefloquine derivatives of *threo* configuration, which were not delivered through the alkylation–Mitsunobu sequence starting from the *erythro* isomer. An attempt was made to repeat the previous sequence of transformations with *threo*-mefloquine as a starting material. We followed partially a method for the inversion of *erythro*-mefloquine (**1**) into the *threo*-isomer **8** (*syn*)^11^ (Scheme 4). Here, rather than resorting to N,O-diacetylation and subsequent ester hydrolysis, we performed a single-step selective acetylation of mefloquine (**1**) at position 13 with acetic anhydride in 2-propanol in the presence of K_2_CO_3_. The 13-acetyl derivative was reacted with thionyl chloride and hydrolyzed to provide *threo*-mefloquine (**8**) hydrochloride in a 94% yield.^11^

**Scheme 5:**
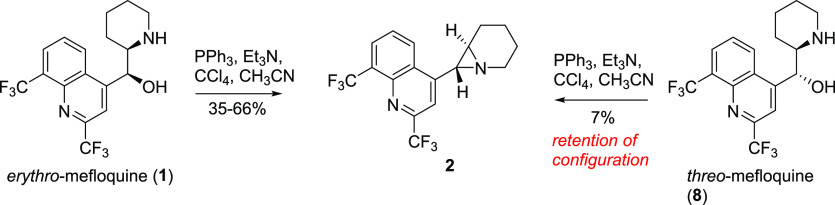
Stereoconvergent Formation of Aziridine **2**

The Mitsunobu reaction proved to be ineffective in converting *threo*-mefloquine directly into an azide while the application of Appel conditions to obtain aziridine resulted in a mixture, from which only a small quantity of aziridine **2** identical to that obtained from *erythro*-mefloquine could be isolated by chromatography with mass spectrometry detection (Scheme 5). The low yield may have resulted from the instability of the expected diastereomer of aziridine **2**. The net retention of the configuration toward aziridine **2** could be explained by two sequential S_N_2 displacements: transient substitution with a chloride ion followed by ring-closing reaction.

**Scheme 6:**
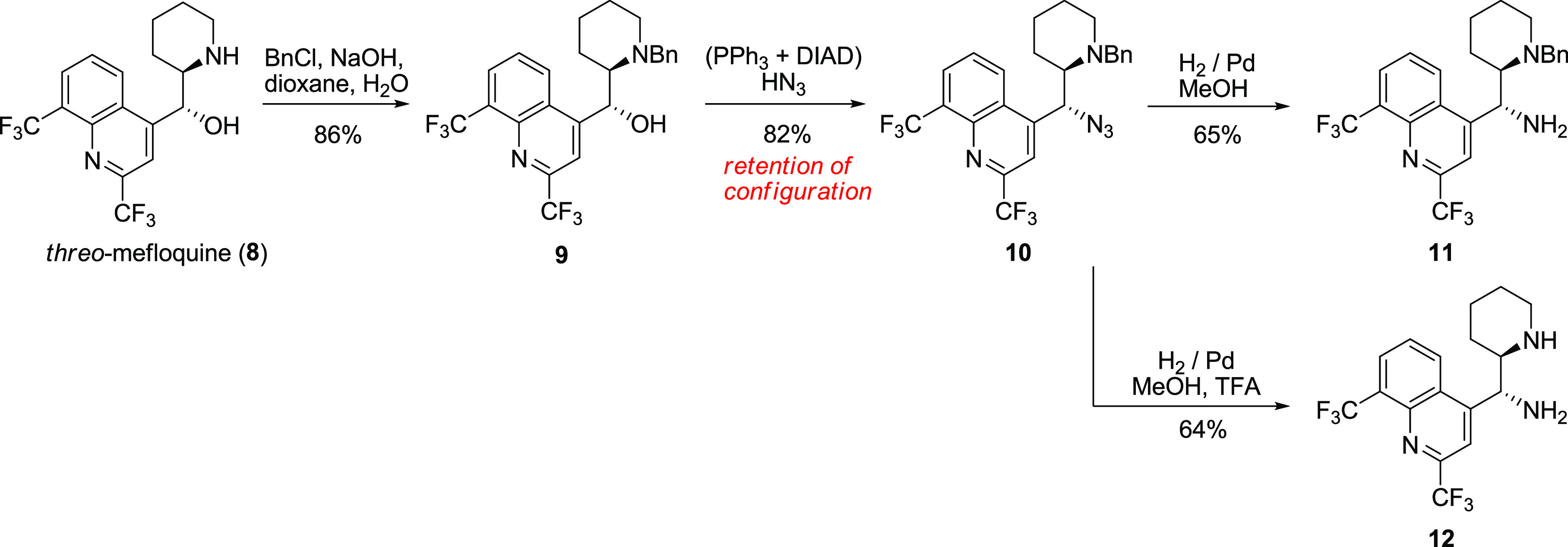
Transformation of *threo*-Mefloquine (**8**) to *threo-*11-Aminomefloquine (**12**)

The reaction of *threo*-mefloquine with benzyl bromide led to the corresponding *N*-alkyl derivative **9**. The displacement of the hydroxyl group under Mitsunobu conditions delivered the respective azide **10** in good yield. The hydrogenation of **10** under acidic and nonacidic conditions finally provided the primary–secondary diamine **12** and primary–tertiary diamine **11** (Scheme 6).

**Scheme 7:**
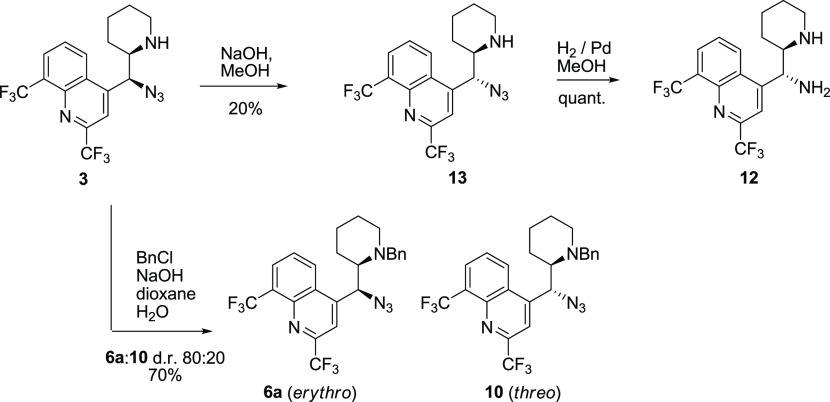
Epimerization of *erythro*-11-Azidomefloquine (**3**) and Alternative Synthesis of *threo*-11-Aminomefloquine (**12**)

The pair of azides **10** and **6a**, as well as the pair of vicinal diamines **4** and **12**, display different NMR spectra. Each mixture of diastereomeric pairs of compounds revealed two distinct sets of signals that did not converge. Partial epimerization at position 11 was also achieved by benzylation of azide **3** under the Schotten–Bauman conditions (dioxane/aqueous NaOH). The resulting mixture contained 20% of *threo*-azide **10** along with the *erythro*-isomer **6a**. Azide **3** could be epimerized under moderately basic conditions (NaOH in MeOH) into a new product **13**, which was also isolated in 20% yield. The hydrogenation of **13** quantitatively yielded diamine **12** converging with the *threo*-mefloquine sequence (Scheme 7). It is noteworthy that NaOH in methanol is insufficient to induce an observable epimerization of 9*S*-azidoquinine. Thus, the electron-withdrawing substituents in the quinoline ring are needed for epimerization to occur at such moderately basic conditions.

In yet another approach, we independently treated 13-benzyl-mefloquine isomers **5a** and **9** with thionyl chloride to obtain intermediate 11-chloro derivatives. Both these transformations converged toward an identical mixture of 11-chloro derivatives. In the NMR spectra, 11-chloro derivative·HCl displayed multiple equilibrating species as evidenced by the NOESY/EXSY experiment (for details, see the Supporting Information). The subsequent reaction with sodium azide resulted in azide **6a** of *erythro* configuration. The yield of the final transformation was low (up to 20%), and the purity of the product was unsatisfactory. We hypothesize that the net inversion of *threo* compound **9** to *erythro* isomer **6a** is a result of three consecutive S_N_2 displacements involving the formation of aziridinium ion.

### Assignment of Relative Configuration

**Figure 2:**
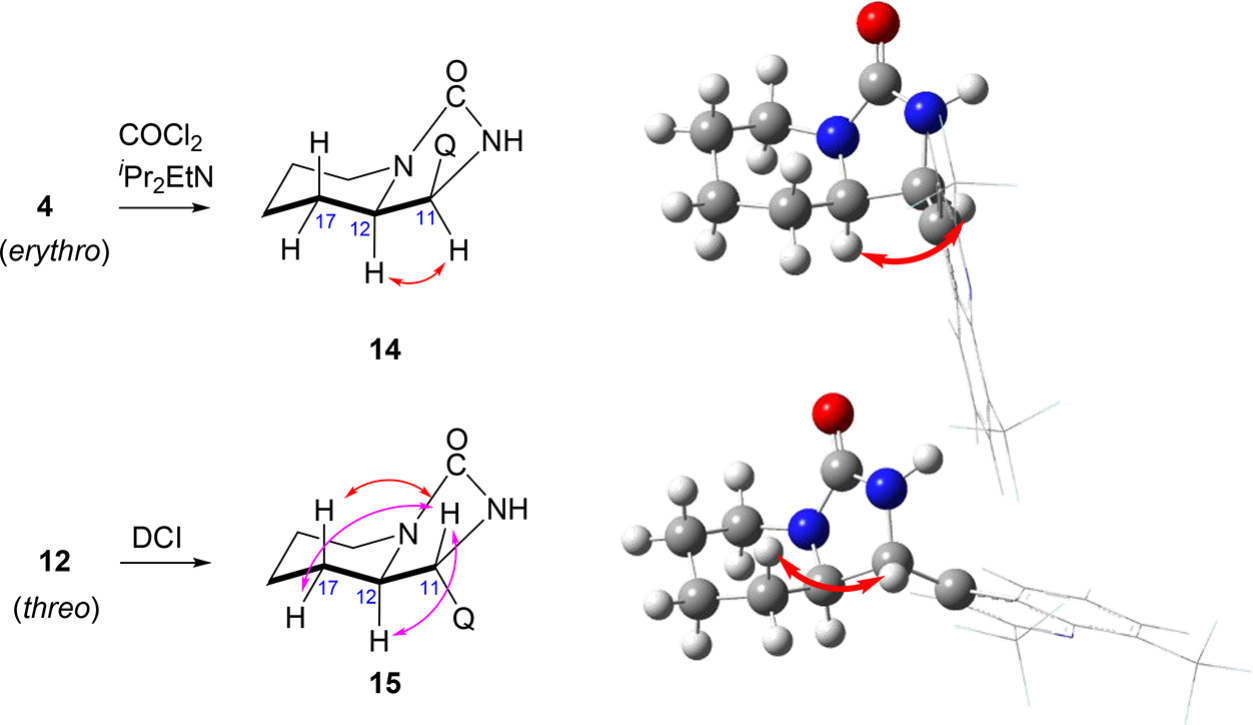
Formation of cyclic urea **14** and **15** derived from 11-aminomefloquines **4** and **12**, diagnostic interactions observed in NOESY spectra, and their molecular models at the DFT/B3LYP/cc-pVDZ level of theory (red, strong correlation, magenta, medium intensity correlation, Q = 2,8-bis(trifluoromethyl)quinolin-4-yl).

The initial assignment of the relative configuration was based on chemical correlation and on the published data on the structure of aziridine **2** and the identification of *erythro* product obtained after aziridine ring opening with acetyl anhydride.^9^ Unequivocal proof for the relative configuration of both target 11-aminomefloquines **4** and **12** was based on the NMR experiments. The diamines were first converted to rigid cyclic urea derivatives **14** and **15**. Only the *erythro* configuration for **4/14** and *threo* for **12/15** could explain the observed NOESY interactions (Figure 2). For the compound of *threo* configuration, the H-11 displayed strong correlation with axial H-17, and weaker correlations with equatorial H-17 and neighboring H-12. In the molecular model of **15** optimized at the DFT/B3LYP/cc-pVDZ level of theory, the corresponding distances from H-11 to H-17(axial), H-17(equatorial), and H-12, were 2.53, 2.62, and 2.79 Å, respectively. The same order of proximity was observed in an X-ray structure of a urea derived from 2-aminomethylpiperidine.^12^ On the other hand, in the *erythro* isomer H-11 displayed a strong correlation only with the neighboring H-12. In the molecular model of **14**, the distance for the observed contact is 2.33 Å, while distances from H-11 to other hydrogen atoms of the piperidine ring are greater than 3.7 Å. The coupling constants between H-11 and H-12 were 8.7 Hz for **14** and 5.5 Hz, for **15**. This experimental finding is congruent with the molecular model, where a nearly perpendicular (dihedral of 100°) arrangement of these hydrogen atoms is expected for the *threo* isomer, while dihedral of −30° was the optimized result for the *erythro* isomer. Unscaled spin coupling calculation at the GIAO/mPW1PW91/6-311+G(2d,p) level predicts 7.5 Hz for *erythro* and 0.7 Hz *threo* isomers.

### Enantiomerically Enriched Products

The initial experiments were performed on commercial racemic mefloquine samples. Enantiomerically pure products were obtained by resolution of diastereomeric salts. In one approach, we crystallized salts of diamine **4** with chiral acids. Separation was successful when (+)-mandelic acid was used. With ethanol, the enantiomeric excess reached 98.5% for (+)-**4** with a 23% yield after single crystallization. Lower selectivity was observed for methanol (77% ee, 43% yield), but it could be improved with recrystallization. The collected crystals contained equimolar quantities of **4**, mandelic acid, and the respective alcohol as crystallization solvent. No resolution of enantiomers was observed for crystals in other solvents (1-propanol, 2-propanol, 1-butanol, ethyl acetate, acetonitrile, dioxane, and water–ethanol mixture). For measuring enantiomeric excess, the diamine **4** or its salt was briefly treated with acetic anhydride at room temperature to convert it to diacetamide **16**, which separates on standard chiral HPLC columns.

Another approach consisted of obtaining (+)-enantiomer of *erythro*-mefloquine ((+)-**1**) with (−)-ditoluyltartaric acid by reiterating a patented report.^13^ Crystallization crops from ethyl acetate removed most of one enantiomer from the supernatant. Recycling of free (−)-mefloquine base from this enriched solution followed by crystallization with (+)-ditoluyltartaric acid led to a sample of enantiomeric purity exceeding 99%. The overall yield for pure enantiomers was 86% and additional 10% of essentially unresolved material could be recovered. For the purpose of measuring the enantiomeric purity, *erythro*-mefloquine was converted to the *N*,*O*-diacetyl compound^9^ with acetyl anhydride.

The retention times of the *N*,*O*-diacetyl derivative of parent aminoalcohol and *N*,*N′*-diacetyl derivative **16** of the same configuration are similar. The signs of optical rotation are the same for amino alcohol **1** and diamine **4** sharing the same configuration. The 11*R*,12*S* stereochemistry of (−)-mefloquine has been previously established unambiguously.^6,14^

The conversion of enantiomerically pure mefloquine into **4** or **12** as shown in Schemes 1 and 6 resulted in essentially the same yields as with the racemic compound. The reactions proceeded without erosion of optical purity, as proven by HPLC chromatography of diacetyl derivatives of the initial material, intermediate azide **3** and the final product after derivatization (**16**). Unlike single site epimerization, racemization is rather unlikely because it requires a change of configuration at two stereogenic centers.

### Example of Catalytic Activity

**Figure 3:**
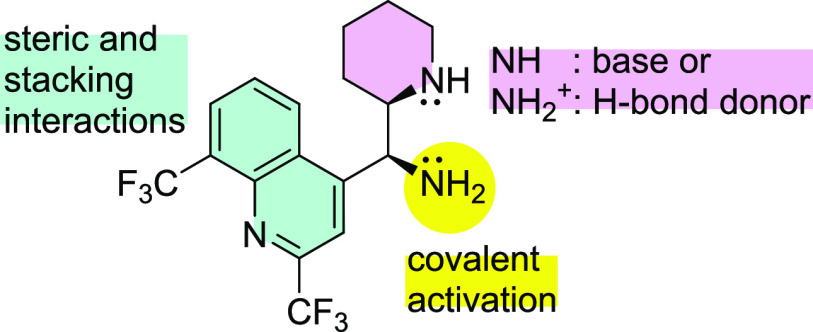
Features of 11-aminomefloquine catalyst.

From the perspective of potential catalytic applications, enantiomerically pure 11-aminomefloquine **4** and **12** offer two activation sites: the primary and secondary amine.^15^ The NH_2_ group was devised to form a covalent bond and thus to activate carbonyl derivatives by lowering LUMO energy in the expected intermediate iminium or iminium ion.^16^ The ancillary secondary amine unit provides a basic center to activate a nucleophile (Figure 3).

To support our hypothesis on the central role of the secondary amine unit in the catalyst’s structure, we have chosen the addition of nitromethane to cyclohexenone as a model transformation. In this type of reaction, the major challenge is attributed to the anomalous nitroalkane proton transfer reflecting the pivotal role of the structure and strength of the applied base. Successful control of chirality transfer in reactions applying nitroalkanes required multifunctional catalysts decorated with additional groups such as hydroxyl group in a set of aminoindanol or aminophenol-based hydrogen bond donors.^17^ In our hands, the chiral primary–secondary diamines gave appreciable level of enantioselection. The reaction catalyzed by a combination of (+)-*erythro*-diamine **4** and achiral carboxylic acid provided up to 93.5% ee (Table 1).

**Table 1:**
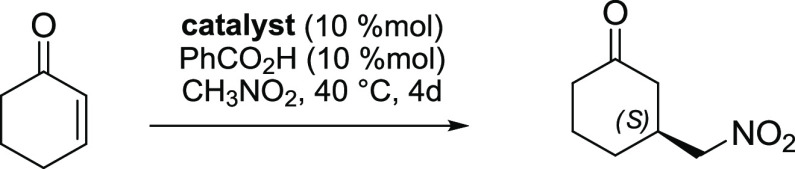
Asymmetric Michael Addition of Nitromethane to Cyclohexenone

entry	catalyst (%), ee of catalyst	yield^a^ (%)	% ee (config)
1	(+)-(11*S*,12*R*)-**4**, >99	44^b^	93.5 (*S*)
2	(+)-(11*S*,12*R*)-**4**, 98	46	92 (*S*)
3	(+)-(11*S*,12*R*)-**4**, 50	46	54 (*S*)
4	(−)-(11*R*,12*S*)-**4**, 98	46	91 (*R*)
5	(−)-(11*R*,12*R*)-**12**, 98	30	77 (*R*)
6	(−)-(11*R*,12*S*)-1, >99	3	37 (*R*)
7	(+)-(11*S*,12*S*)-**8**, 99	2	20 (*S*)
8	(−)-(11*R*,12*S*)-**7b**, >99	22	*rac*
9	(−)-(11*R*,12*S*)-**7a**, >99	6	*rac*
10	(−)-(11*R*,12*R*)-**11**, 98	19	*rac*
11	(9*S*,8*S*)-EAQN,^c^ 100	93	84 (*S*)

a Yield was estimated by quantitative NMR.

b Preparative yield.

c 9-*epi*-Aminoquinine.

**Figure 4:**
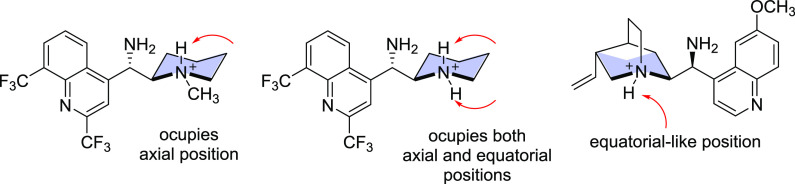
Orientation of hydrogen atoms in protonated catalyst species.

The enantiomeric excess of the formed adduct exhibited a linear correlation with the enantiomeric composition of the applied catalyst (Table 1, entries 1–4). The importance of the primary amine unit was rather undisputable while the parent mefloquine was both unreactive and unselective in this reaction. The diamine **4** outperformed in terms of selectivity even 9-deoxy-*epi*-aminoquinine (Table 1, entry 11), which gave only 84% ee but nearly quantitative yield. The *threo* isomer **12**, which shares the same relative configuration as this alkaloid derivative, delivered moderate enantioselectivity as well. The superiority of secondary amine over tertiary amine was proven for **7b**, **7a**, and **11**, which are *N*-methyl and *N*-benzyl derivatives of 11-aminomefloquine of the *erythro* and *threo* configurations. No chirality transfer was observed there (Table 1, entries 8–10). In protonated form (NH_2_^+^), any of the two differently oriented hydrogen atoms in **4**·H^+^ may interact with another species via hydrogen bonds, which cannot be achieved for analogous *Cinchona* derivatives (Figure 4). We perceive the presence of the NH proton at the equatorial position to be pivotal for achieving high enantioleselectivities. When a proton attached to the tertiary nitrogen atom of quinuclidine in *epi*-aminoquinine occupies an equatorial-like position, chiral discrimination was provided albeit at a lower level.

## Conclusions

We have demonstrated an efficient method for the preparation of chiral primary-secondary diamines from mefloquine. No chromatography was required to obtain high yields of the *erythro*-isomer of 11-aminomefloquine (**4**). Most of the studied transformations proceeded with a net retention of the configuration involving aziridine or aziridinium ion participation. Product **4** was proven to provide effective chirality transfer in an asymmetric Michael addition, indicating the crucial role of the combination of primary and secondary amine groups. This feature as well as the availability of enantiomers rather than diastereomers is distinct from the natural product-based catalysts. We anticipate that 11-aminomefloquines and their further derivatives could provide an interesting alternative to the well-established 9-amino-*Cinchona* alkaloid framework, although the starting material may be up to 10 times more expensive.

## Experimental Section

### General Comments

All reagents were obtained from commercial suppliers and used as received. Racemic mefloquine HCl was sourced from China by a local chemical distributor. Reported procedures were used for the preparation of **8** from acetyl mefloquine^11^ and compound **5b**.^18^ NMR spectra were recorded on 400 and 600 MHz instruments with TMS as an internal standard for ^1^H and ^13^C spectra (δ_H_ = 0 and δ_C_ = 0) while ^19^F NMR spectra were internally referenced to α,α,α-trifluorotoluene (PhCF_3_, δ_F_ = −63.72 ppm). HRMS spectra were obtained on a high-resolution TOF mass spectrometer with an electron spray ionization (ESI) source. Optical rotations were measured in 10 cm tube on an automatic polarimeter with sodium lamp. Melting points are uncorrected. HPLC chromatography was performed on IA-3 and IC-3 4.6 × 250 mm columns at 40 and 22 °C, respectively with UV detection. Flash chromatography was performed on standard silica gel 60, 230–400 mesh, Brockman I active neutral, basic, or acidic aluminum oxide.

### General Procedure for Liberation of Mefloquine Free Base^13^

Mefloquine salt (25 mmol) was suspended in methanol (70 mL) and stirred. Aqueous NaOH (1M, 300 mL, 12 equiv) was added via a dropping funnel for 0.2 h. Stirring was continued for 18 h at room temperature. Precipitate was collected by filtration and washed with water (8 × 25 mL). The white solid was air-dried and then vacuum-dried at 85 °C for 6 h. Yield 92–98%.

### Separation of *erythro*-Mefloquine Enantiomers

*erythro*-Mefloquine free base (**1**, 21.4 g, 56.5 mmol) was dissolved in EtOAc (800 mL), and a solution of (+)-*O*,*O*-di(*p*-toluyl)tartaric acid (22.7 g, 58.9 mmol, 1.04 equiv) in EtOAc (160 mL) was added. The mixture was stirred for 24 h, and the precipitated wool-like crystals were separated by filtration and washed with EtOAc (200 mL). The solid and the filtrate were processed separately: the solid was triturated with EtOAc (350 mL) for 1 h at reflux (water bath) and 24 h at room temperature, collected by filtration, washed with EtOAc (100 mL), and air-dried to obtain white crystalline salt of (−)-mefloquine (20.3 g, 47%). The liberation of the free base according to the general procedure afforded 9.04 g (42%, >98% ee) of (−)-(11*R*,12*S*)-**1**.

The filtrate was concentrated to ca. half of the volume and stored for 24 h at room temperature. The precipitate was removed by filtration and the solution concentrated to afford an oily residue containing acid and enriched (+)-mefloquine (up to 90% ee). The liberation of free base according to the general procedure and repeated separation with 1 equv of (−)-*O*,*O*-di(*p*-toluyl)tartaric acid yielded crystalline salt of (+)-mefloquine. The liberation of free base according to the general procedure afforded 9.37 g (44%, 99.7% ee) of (+)-(11*S*,12*R*)-**1**.

Removed washings and precipitate were combined, and after the liberation of the free base, (±)-mefloquine was recovered in ca. 10% yield.

The determination of enantiomer composition: a sample of **1** (ca. 1–2 mg) was dissolved in acetic anhydride and stirred at 110 °C for 3 h. The sample was concentrated in vacuo. HPLC (IA-3, 2-propanol:hexane 1:9, 1 mL/min, λ = 285 nm) *t*_R_ = 6.5 min for (+)-(11*S*,12*R*)-**1** and 10.1 min for (−)-(11*R*,12*S*)-**1**

### *erythro*-11-Azidomefloquine Hydrazoic Acid Salt (**3**·HN_3_)

*erythro*-Mefloquine free base (**1**, 6.55 g, 17.3 mmol) was suspended in acetonitrile (50 mL) under argon atmosphere. Next, triphenylphosphine (4.77 g, 18.2 mmol, 1.05 equiv) and triethylamine (2.41 mL, 17.3 mmol, 1.0 equiv) were added. After 5 min, CCl_4_(1.68 mL, 17.3 mmol, 1.0 equiv) was added dropwise. Within 5–10 min the mixture formed a solution, then gradual precipitation occurred. After 3 days, the solvent was removed, and the residue was suspended in Et_2_O (50 mL). The mixture was filtered, and the separated solid was extracted with Et_2_O (2 × 25 mL). The combined filtrate was concentrated *in vacuo*, providing crude *threo*-mefloquine aziridine **2** as yellow oil (7.90 g).

Crude *threo*-mefloquine aziridine (7.90 g) was dissolved in a 1.67 M solution of hydrazoic acid in benzene (35 mL, 58.5 mmol of HN_3_). Pure product **3** crystallized from the solution within 24 h as hydrazoic acid salt. The product was filtered, washed with benzene, and air-dried to afford 5.79 g of colorless to light yellow crystals (75%). Chromatography of the mother liquor on silica gel (CH_2_Cl_2_/MeOH 50:1 then 10:1) gave 0.48 g of side product **3b** and an additional portion of 0.45 g of **3** (total 81.5%). The ^1^H NMR spectra of **3** hydrazoic acid salt and **3** free base display no differences. Mp = 133–140 °C (dec, MeOH). ^1^H NMR (400 MHz, CDCl_3_, TMS) δ = 8.41 (d, *J* = 8.6 Hz, 1H), 8.22 (d, *J* = 7.2 Hz, 1H), 7.93 (s, 1H), 7.80 (t, *J* = 8.0 Hz, 1H), 5.24 (d, *J* = 6.0 Hz, 1H), 3.03–3.07 (m, 1H), 2.93–2.98 (m, 1H), 2.55 (td, *J* = 12.2, 2.7 Hz, 1H), 2.27 (br s, 2H), 1.84–1.87 (m, 1H), 1.65–1.68 (m, 1H), 1.57–1.60 (m, 1H), 1.20–1.43 (m, 3H) ppm. ^13^C{^1^H} NMR (100 MHz, CDCl_3_, TMS) δ = 148.4 (q, *J* = 35.6 Hz), 146.5, 144.3, 129.9 (q, *J* = 30.4 Hz), 129.4 (q, *J* = 5.4 Hz), 127.9, 127.6, 127.2, 123.5 (q, *J* = 274.0 Hz), 121.2 (q, *J* = 275.7 Hz), 116.7 (q, *J* = 2.0 Hz), 67.6, 60.6, 47.2, 28.1, 26.2, 24.2 ppm. ^19^F NMR (376 MHz, CDCl_3_, PhCF_3_) δ = −61.32 (s, 3F), −68.86 (s, 3F) ppm. HRMS (ESI-TOF) [C_17_H_15_F_6_N_5_ + H]^+^*m*/*z* calcd: 404.1304, found 404.1306. Anal. Calcd for C_17_H_15_F_6_N_5_·HN_3_ (446.36): C, 45.74; H, 3.61; N, 25.10. Found: C, 45.93; H, 4.23; N, 24.74. HPLC (IA-3, 2-propanol/hexane 1:9, 1 mL/min, λ = 282 nm) *t*_R_ = 4.8 min for (11*R*,12*S*)-**3** and 5.5 min for (11*S*,12*R*)-**3**.

From (−)-(11*R*,12*S*)-*erythro*-mefloquine (11*R*,12*S*)-**3**·HN_3_ was obtained: [α]_D_^24^ = −41 (*c* 1, MeOH). From (+)-(11*S*,12*R*)-*erythro*-mefloquine (11*S*,12*R*)-**3**·HN_3_ was obtained: [α]_D_^20^ = +46 (*c* 1, MeOH).

### (6*S*,7*S*)-7-(2,8-Bis(trifluoromethyl)quinoline-4-yl-1-azabicyclo[4.1.0]heptane (**2**)

Following the procedure for the synthesis of **3**, starting from (−)-(11*R*,12*S*)-mefloquine (**1**, 9.14 g, 24 mmol), intermediate crude **2** was purified by chromatography on neutral Al_2_O_3_ (hexane/EtOAc 1:0–10:1), and then double recrystallization from petroleum ether at −20 °C^9^ gave white wool-like crystals 4.82 g (55%). Mp = 78.5–79 °C. [α]_D_^25^ = +136 (*c* 1, C_6_H_6_). ^1^H NMR data matched literature data for racemic compound.^9^^1^H NMR (600 MHz, CDCl_3_, TMS) δ = 8.38 (d, *J* = 8.4 Hz, 1H), 8.15 (d, *J* = 7.2 Hz, 1H), 7.86 (s, 1H), 7.73 (t, *J* = 7.9 Hz, 1H), 3.56–3.62 (m, 1H), 3.22 (d, *J* = 2.1 Hz, 1H), 3.03 (ddd, *J* = 13.6, 8.4, 5.4 Hz, 1H), 2.15–2.26 (m, 4H), 1.52–1.66 (m, 4H) ppm. ^13^C{^1^H} NMR (151 MHz, CDCl_3_, TMS) δ = 150.5, 148.8 (q, *J* = 35.0 Hz), 143.4, 129.4 (q, *J* = 30.1 Hz), 128.6 (q, *J* = 5.5 Hz), 128.3, 127.5, 126.8, 123.6 (q, *J* = 273.8 Hz), 121.3 (q, *J* = 275.1 Hz), 114.4 (q, *J* = 2.1 Hz), 48.5, 42.9, 41.7, 22.1, 21.1, 18.2 ppm.

For racemic compound **2**. Mp = 84.5–87 °C (lit.^9^ mp = 85.5–87 °C).

### (2*R*,3*S*)-3-Azido-2-(2,8-bis(trifluoromethyl)quinolin-4-yl)azepane (**3b**)

The title product was isolated as described in the preparation of **3** from (+)-(11*S*,12*R*)-**1** in a 7% yield (0.48 g) as a yellow crystalline solid. Mp = 93–96 °C. [α]_D_^20^ = −29 (*c* 1, MeOH). ^1^H NMR (400 MHz, CDCl_3_, TMS) δ = 8.61(d, *J* = 8.6 Hz, 1H), 8.19 (d, *J* = 7.2 Hz, 1H), 7.92 (s, 1H), 7.76 (t, *J* = 8.0 Hz, 1H), 4.22 (d, *J* = 9.0 Hz, 1H), 3.89–3.93 (m, 1H), 3.28–3.34 (m, 1H), 2.82–2.89 (m, 1H) 2.31–2.38 (m, 1H), 1.99–2.07 (m, 1H), 1.87–1.93 (m, 2H), 1.78–1.84 (m, 2H), 1.58–1.63 (m, 1H) ppm. ^13^C{^1^H} NMR (151 MHz, CDCl_3_, TMS) δ = 152.3, 148.5 (q, *J* = 35.4 Hz), 144.4, 129.6 (q, *J* = 30.1 Hz), 129.2 (q, *J* = 5.3 Hz), 128.8, 127.5, 127.2, 123.7 (q, *J* = 273.5 Hz), 121.3 (q, *J* = 275.6 Hz), 116.1, 68.2, 66.8, 50.6, 31.9, 30.5, 21.7 ppm. ^19^F NMR (376 MHz, CDCl_3_, PhCF_3_) δ = −61.30 (s, 3F), −68.86 (s, 3F) ppm. HRMS (ESI-TOF) [C_17_H_15_F_6_N_5_ + H]^+^*m*/*z* calcd: 404.1304, found: 404.1308

For racemic compound **3b**. Mp = 70–74 °C.

### *erythro*-11-Aminomefloquine (**4**)

*erythro*-11-Azidomefloquine hydrazoic acid salt (**3**·HN_3_) (5.79 g, 13.0 mmol) was dissolved in methanol (110 mL), and palladium on carbon was added (5%, 103 mg, 0.4mol %). The reaction vessel was loaded with hydrogen (6.0 bar), and the mixture was stirred for 3 h. After that time, the mixture was filtered, and the solvent was removed. Aminomefloquine **4** (4.89 g) was obtained in a quantitative yield as a white crystalline solid. Mp: 77 °C (MeOH). ^1^H NMR (400 MHz, CDCl_3_, TMS) δ = 8.45 (d, *J* = 8.3 Hz, 1H), 8.17 (d, *J* = 7.5 Hz, 1H), 8.10 (s, 1H), 7.77 (dd, *J* = 8.3, 7.5 Hz, 1H), 4.79 (d, *J* = 5.3 Hz, 1H), 3.03–3.07 (m, 1H), 2.90–2.96 (m, 1H), 2.57–2.62 (m, 1H), 1.78–1.83 (m, 1H), 1.47–1.65 (m, 5H), 1.28–1.41 (m, 2H), 1.17–1.26 (m, 1H) ppm. ^13^C{^1^H} NMR (100 MHz, CDCl_3_, TMS) δ = 152.8, 148.4 (q, *J* = 35.3 Hz), 144.0, 129.6 (q, *J* = 30.2 Hz), 128.9 (q, *J* = 5.5 Hz), 127.9, 127.8, 127.0, 123.7 (q, *J* = 274.9 Hz), 121.4 (q, *J* = 276.9 Hz), 115.8 (q, *J* = 2.1 Hz) 61.9, 56.0, 47.4, 27.0, 26.2, 24.5 ppm. ^19^F NMR (376 MHz, CDCl_3_, PhCF_3_) δ = −61.29 (s, 3F), −68.86 (s, 3F) ppm. HRMS (ESI-TOF) [C_17_H_17_F_6_N_3_ + H]^+^*m*/*z* calcd: 378.1399, found: 378.1396.

Enantiomerically pure products were pale amorphous solids. For (11*S*,12*R*)-**4**. [α]_D_^25^ = +46 (*c* 1.1, MeOH). For (11*R*,12*S*)-**4**. [α]_D_^24^ = −48 (*c* 1.3, MeOH).

### Separation of (±)-**4** with l-Mandelic Acid

Alcohol (ethanol or methanol) solutions of (+)-l-mandelic acid (0.62 g in 5 mL, 1 equiv) and racemic **4** (1.5 g, 4.1 mmol, in 5 mL) were mixed and stored at room temperature, at 4 °C and at −20 °C, sequentially for 24 h intervals. The white solid was removed by filtration and washed with cold alcohol. Sample obtained from methanol (1.03 g) was recrystallized from the same solvent to give 0.73 g of white crystals. For (+)-(11*S*,12*R*)-**4**l-mandelic acid salt·MeOH, 95% ee. Mp = 167–169 °C (MeOH).

For (+)-(11*S*,12*R*)-**4**l-mandelic acid salt EtOH, 98% ee: Mp = 157–166 °C (EtOH).

The l-mandelic acid salt of (11*S*,12*R*)-**4** methanol solvate (1.15 g, 2.05 mmol) was suspended in a mixture of aqueous NaOH (10%, 7 mL) and CH_2_Cl_2_ (20 mL). After 5 min of stirring, the mixture was separated and the aqueous layer extracted with CH_2_Cl_2_ (3 × 15 mL). Combined organic phases were dried over anhydrous Na_2_SO_4_ and evaporated to give (11*S*,12*R*)-**4** free base as pale oil (697 mg, 90%). [α]_D_^20^ = +44 (*c* 1.1, MeOH).

### *erythro*-13-Acetyl-11-acetamidomefloquine (**16**)

*erythro*-11-Aminomefloquine (**4**) (22 mg, 0.06 mmol) was dissolved in acetic anhydride (0.5 mL). After 1 h, the solvent was removed in vacuo, and the product was obtained as white crystals (27 mg, quantitative yield). For the purpose of measuring enantiomeric purity, salts of **4** were treated directly with acetic anhydride as described above. ^1^H NMR (600 MHz, CDCl_3_, TMS) δ = 8.86 (d, *J* = 8.7 Hz, 1H), 8.19 (d, *J* = 7.4 Hz, 1H), 8.07 (s, 1H), 7.82 (dd, *J* = 8.7, 7.4 Hz, 1H), 6.53 (t, *J* = 9.5 Hz, 1H), 5.51–5.57 (m, 1H), 3.29 (dd, *J* = 14.3, 3.3 Hz, 1H) 2.50 (td, *J* = 14.3, 2.5 Hz, 1H), 2.04–2.15 (m, 1H), 2.03 (s, 3H), 1.95–2.01 (m, 1H), 1.88 (s, 3H), 1.63–1.81 (m, 4H) ppm. ^13^C{^1^H} NMR (151 MHz, CDCl_3_, TMS) δ = 170.2, 170.1, 148.2 (q, *J* = 35.3 Hz), 147.8, 144.3, 129.6 (q, *J* = 29.6 Hz), 129.2 (q, *J* = 5.4 Hz), 128.4, 128.3, 128.0, 123.7 (q, *J* = 273.7 Hz), 121.4 (q, *J* = 275.6 Hz), 116.4, 50.8, 46.7, 43.1, 25.3, 25.1, 23.1, 21.5, 19.4 ppm. ^19^F NMR (376 MHz, CDCl_3_, PhCF_3_) δ = −61.26 (s, 3F), −68.95 (s, 3F) ppm. HPLC (IA-3, 2-propanol/hexane 1:9, 1 mL/min, λ = 285 nm) *t*_R_ = 5.9 min for (11*S*,12*R*)-**16** and 10.8 min for (11*R*,12*S*)-**16**. HRMS (ESI-TOF) [C_21_H_21_F_6_N_3_O_2_ + H]^+^*m*/*z* calcd: 462.1611, found: 462.1659 [C_21_H_21_F_6_N_3_O_2_ + Na]^+^*m*/*z* calcd: 484.1430, found: 484.1428

### *erythro*-13-Benzylmefloquine (**5a**)

*erythro*-Mefloquine hydrochloride (6.01 g, 14.5 mmol) was suspended in a mixture of dioxane (50 mL) and aqueous NaOH (15%, 20 mL). After 5 min of stirring, benzyl chloride (1.90 mL, 16.5 mmol, 1.14 equiv) was added dropwise. The mixture was stirred for 6 days at room temperature. Subsequently, the phases were separated and the aqueous layer was extracted with CH_2_Cl_2_ (3 × 15 mL). Combined organic phases were dried over anhydrous Na_2_SO_4_, and the solvents were evaporated. The crystallization from methanol (35 mL) provided 4.65 g (69%) of colorless crystals. Mp: 151–152 °C (MeOH). ^1^H NMR (400 MHz, CDCl_3_, TMS) δ = 8.22 (s 1H), 8.15 (d, *J* = 7.2 Hz, 1H), 8.08 (d, *J* = 8.5 Hz, 1H), 7.70 (t, *J* = 7.9 Hz, 1H), 7.38–7.43 (m, 4H), 7.31–7.36 (m, 1H), 6.01 (d, *J* = 3.2 Hz, 1H), 4.58 (d, *J* = 13.2 Hz, 1H), 4.35 (br s, 1H), 3.44 (d, *J* = 13.2 Hz, 1H), 3.04–3.07 (m, 1H), 2.85 (dt, *J* = 11.3, 3.5 Hz, 1H), 2.23 (td, *J* = 11.9, 2.5 Hz, 1H), 1.53–1.61 (m, 2H), 1.31–1.47 (m, 2H), 0.94–1.04 (m, 1H), 0.60–0.63 (m, 1H) ppm. ^13^C{^1^H} NMR(100 MHz, CDCl_3_, TMS) δ = 150.5, 148.5 (q, *J* = 35.2 Hz), 143.8, 138.2, 129.8 (q, *J* = 30.1 Hz), 129.0, 128.8, 128.7 (q, *J* = 5.5 Hz), 127.7, 127.06, 127.02, 126.6, 123.7 (q, *J* = 273.7 Hz), 121.5 (q, *J* = 275.6 Hz), 115.9 (q, *J* = 2.1 Hz), 67.7, 63.6, 58.7, 53.5, 25.3, 24.9, 23.4 ppm. HRMS (ESI-TOF) [C_24_H_22_F_6_N_2_O + H]^+^*m*/*z* calcd: 469.1709, found: 469.1709.

For (11*R*,12*S*)-**5a**. [α]_D_^25^ = −23 (*c* 1, MeOH).

### *erythro*-11-Azido-13-benzylmefloquine (**6a**)

#### Method A

Triphenylphosphine (3.68 g, 14.03 mmol, 2.0 equiv) was dissolved in a mixture of toluene (40 mL) and THF (22.5 mL). Then diisopropyl azadicarboxylate (DIAD, 3.04 mL, 15.43 mmol, 2.2 equiv) was added dropwise at 0 °C, and the suspension was stirred for 10 min. In another flask, *erythro*-13-benzylmefloquine (**5a**, 3.29 g, 7.03 mmol) was dissolved in THF (16.5 mL), cooled to 0 °C, mixed with diphenylphosphoryl azide (DPPA, 3.03 mL dropwise, 14.03 mmol, 2.0 equiv), and stirred for 10 min under argon. Then, this mixture was added at 0 °C to the previously prepared suspension of Ph_3_P and DIAD. The mixture was allowed to slowly attain room temperature and stirred for 18 h. The solution was concentrated and the crude product was purified using column chromatography on silica gel (hexane/EtOAc 9:1), giving 3.54 g of yellowish amorphous solid containing 17% of triphenylphosphine oxide (86% yield).

#### Method B

*erythro*-11-Azidomefloquine hydrazoic acid salt (**3·**HN_3_, 0.353 g, 0.791 mmol) and *N,N*-diisopropylethylamine (0.414 mL, 2.43 mmol, 3.08 equiv) were dissolved in DMF (8 mL). Then benzyl bromide (0.170 mL, 1.43 mmol, 1.81 equiv) was added. The mixture was kept for 18 h at room temperature. The mixture was concentrated in vacuo, diluted with ethyl acetate (25 mL), washed with saturated aqueous NaHCO_3_ (25 mL), and brine (3 × 25 mL), and dried over anhydrous Na_2_SO_4_. The crude product was purified on a silica gel column (hexane/EtOAc 9:1) to give a light yellow amorphous solid (0.278 g, 71% yield). ^1^H NMR(400 MHz, CDCl_3_, TMS) δ = 8.15 (d, *J* = 7.2 Hz, 1H), 8.02 (d, *J* = 8.5 Hz, 1H), 7.87 (s, 1H), 7.64 (t, *J* = 7.9 Hz, 1H), 7.16–7.19 (m, 3H), 7.08–7.10 (m, 2H), 5.77 (d, *J* = 5.5 Hz, 1H), 4.27 (d, *J* = 13.2 Hz, 1H), 3.54 (d, *J* = 13.2 Hz, 1H), 3.06–3.11 (m, 1H), 2.86–2.90 (m, 1H), 2.33–2.39 (m, 1H), 1.70–1.85 (m, 2H), 1.57–1.63 (m, 1H), 1.45–1.53 (m, 1H), 1.27–1.41 (m, 2H) ppm. ^13^C{^1^H} NMR (151 MHz, CDCl_3_, TMS) δ = 148.1 (q, *J* = 35.5 Hz), 147.6, 144.2, 138.6, 129.8 (q, *J* = 30.3 Hz), 129.0 (q, *J* = 5.4 Hz), 128.6, 128.5, 127.40, 127.36, 126.9, 123.6 (q, *J* = 273.8 Hz), 121.3 (q, *J* = 275.7 Hz), 117.1 (q, *J* = 1.7 Hz), 63.1, 62.2, 58.8, 51.7, 23.1, 22.9, 22.4 ppm (one signal not observed due to overlap). ^19^F NMR (376 MHz, CDCl_3_, PhCF_3_) δ = −61.32 (s, 3F), −68.86 (s, 3F) ppm. HRMS (ESI-TOF): [C_24_H_21_F_6_N_5_ + H]^+^*m*/*z* calcd: 494.1774, found: 494.1774.

For (11*R*,12*S*)-**6a**. [α]_D_^25^ = −13 (*c* 0.9, MeOH).

### *erythro*-11-Azido-13-methylmefloquine (**6b**)

#### Method A

Triphenylphosphine (1.47 g, 5.60 mmol, 1.1 equiv) was dissolved in CH_2_Cl_2_ (25 mL), the solution was cooled to 0 °C, and diisopropyl azadicarboxylate (DIAD, 1.00 mL, 5.08 mmol, 1.0 equiv) was added dropwise with stirring. Stirring was continued for 10 min at 0 °C.

In another flask, *erythro*-13-methylmefloquine^18^ (**5b**, 2.00 g, 5.10 mmol) was dissolved in CH_2_Cl_2_ (45 mL), and a solution of hydrazoic acid in benzene (1.67 M, 6.1 mL, 10.2 mmol, 2.0 equiv) was added. After 10 min, the previously prepared mixture of phosphine and DIAD was added. The reaction mixture was allowed to slowly attain room temperature and stirred for 5 days. Then the mixture was concentrated and the crude product was purified using column chromatography on silica gel (CH_2_Cl_2/_EtOAc 9:1), giving 1.45 g of yellowish amorphous solid (68%).

#### Method B

*erythro*-11-Azidomefloquine hydrazoic acid salt (**3·**HN_3_, 522 mg, 1.17 mmol) was suspended in formic acid (0.6 mL) and formaldehyde solution (36–38% aqueous, 0.60 mL). The mixture was heated in an oil bath at 80 °C overnight. Then the suspension was evaporated to dryness and dissolved in CH_2_Cl_2_(30 mL). The organic phase was washed with a saturated solution of NaHCO_3_. The aqueous layer was extracted with CH_2_Cl_2_. The combined organic phases were dried over anhydrous Na_2_SO_4_. The solvent was evaporated giving pure product (450 mg, 92%) as pale crystalline solid. Mp = 114.5–117 °C dec. ^1^H NMR (400 MHz, CDCl_3_, TMS) δ = 8.20–8.23 (m, 2H), 7.98 (s, 1H), 7.81 (t, *J* = 7.9 Hz, 1H), 6.00 (d, *J* = 2.6 Hz, 1H), 3.03–3.06 (m, 1H), 2.72 (s, 3H), 2.34 (dt, *J* = 10.9, 2.6 Hz, 1H), 2.12–2.19 (m, 1H), 1.55–1.70 (m, 4H), 0.89–1.00 (m, 2H) ppm. ^13^C{^1^H} NMR (100 MHz, CDCl_3_, TMS) δ = 148.3 (q, *J* = 35.5 Hz), 146.9, 144.2, 130.0 (q, *J* = 30.5 Hz), 129.1 (q, *J* = 5.5 Hz), 127.8, 126.9, 126.5, 123.5 (q, *J* = 273.8 Hz), 121.2 (q, *J* = 275.8 Hz), 116.9 (q, *J* = 2.1 Hz), 67.2, 61.0, 57.7, 43.5, 25.4, 24.4, 23.8 ppm. ^19^F NMR (376 MHz, CDCl_3_, PhCF_3_) δ = −61.33 (s, 3F), −68.88 (s, 3F) ppm. HRMS (ESI-TOF) [C_18_H_17_F_6_N_5_ + H]^+^*m*/*z* calcd: 418.1461, found: 418.1461.

For (11*R*,12*S*)-**6b**. [α]_D_^24^ = −96 (*c* 1.1, MeOH).

### *erythro*-11-Amino-13-benzylmefloquine (**7a**)

*erythro*-11-Azido-13-benzyl-mefloquine (**6a**, 55.0 mg, 0.112 mmol) was dissolved in methanol (2.5 mL) and palladium on carbon was added (5%, 5.0 mg, 2 mol %). The reaction vessel was loaded with hydrogen (6.0 bar) and the mixture was stirred for 24 h. Then, the mixture was filtered and the solvent was evaporated. The product was obtained by flash chromatography on silica gel (hexane/EtOAc 2:3 to 0:1 gradient) as yellow oil (25 mg, 48% yield).

^1^H NMR(400 MHz, CDCl_3_, TMS) δ = 8.38 (s, 1H), 8.22 (d, *J* = 8.6 Hz, 1H), 8.15 (d, *J* = 7.2 Hz, 1H), 7.70 (t, *J* = 7.9 Hz, 1H), 7.28–7.45 (m, 5H), 5.48 (d, *J* = 3.5 Hz, 1H), 4.57 (d, *J* = 13.4 Hz, 1H), 3.41 (d, *J* = 13.4 Hz, 1H), 2.97–3.04 (m, 1H), 2.65 (dt, *J* = 10.8, 3.3 Hz, 1H), 2.11 (td, *J* = 11.6, 3.0 Hz, 1H), 1.80 (br s, 2H), 1.58–1.71 (m, 2H), 1.36–1.55 (m, 2H), 0.89–1.03 (m, 1H), 0.75–0.84 (m, 1H) ppm. ^13^C{^1^H} NMR (100 MHz, CDCl_3_, TMS) δ = 153.2, 148.4 (q, *J* = 34.8 Hz), 144.1, 139.4, 129.8 (q, *J* = 29.9 Hz), 129.3, 128.7, 128.60, 128.56, 127.6, 127.3, 126.9, 123.8 (q, *J* = 273.8 Hz), 121.6 (q, *J* = 275.6 Hz), 117.1 (q, *J* = 1.9 Hz), 64.7, 58.5, 54.1, 50.5, 25.5, 24.1, 24.0 ppm. ^19^F NMR (376 MHz, CDCl_3_, PhCF_3_) δ = −61.29 (s, 3F), −68.87 (s, 3F) ppm. HRMS (ESI-TOF) [C_24_H_23_F_6_N_3_ + H]^+^*m*/*z* calcd: 468.1869, found: 468.1867.

For (11*R*,12*S*)-**7a**. [α]_D_^22^ = −30 (*c* 0.7, MeOH).

### *erythro*-11-Amino-13-methylmefloquine (**7b**)

*erythro*-11-Azido-13-methylmefloquine (**6b**, 2.26 g, 5.42 mmol) was dissolved in methanol (75 mL), and palladium on carbon was added (5%, 170 mg, 1.6 mol %). The reaction vessel was loaded with hydrogen (6.0 bar), and the mixture was stirred overnight. After that time, the mixture was filtered and the solvent was removed. The crude product was purified using column chromatography on basic Al_2_O_3_ (hexane/EtOAc 1:0 to 0:1 gradient). The product (1.73 g) was obtained in a 82% yield as a light yellow solid. Mp: 128–131 °C. ^1^H NMR (400 MHz, CDCl_3_, TMS) δ = 8.36 (s, 1H), 8.30 (d, *J* = 8.6 Hz, 1H), 8.14 (d, *J* = 7.2 Hz, 1H), 7.72 (t, *J* = 7.9 Hz, 1H), 5.40 (d, *J* = 3.3 Hz, 1H), 2.95–2.98 (m, 1H), 2.62 (s, 3H), 2.24 (dt, *J* = 11.1, 3.0 Hz, 1H), 2.12–2.19 (m, 1H), 1.77 (br s, 2H), 1.49–1.62 (m, 4H), 0.83–0.95 (m, 1H), 0.69–0.74 (m, 1H) ppm. ^13^C{^1^H} NMR (100 MHz, CDCl_3_, TMS) δ = 153.0, 148.3 (q, *J* = 34.7 Hz), 144.0, 129.6 (q, *J* = 29.8 Hz), 128.6 (q, *J* = 5.5 Hz), 127.5, 127.3, 126.9, 123.8 (q, *J* = 273.7 Hz), 121.6 (q, *J* = 274.9 Hz), 117.0 (q, *J* = 2.1 Hz), 66.9, 57.9, 50.4, 43.3, 25.9, 24.1, 23.7 ppm. ^19^F NMR (376 MHz, CDCl_3_, PhCF_3_) δ = −61.27 (s, 3F), −68.85 (s, 3F) ppm. HRMS (ESI-TOF) [C_18_H_19_F_6_N_3_ + H]^+^*m*/*z* calcd: 392.1556, found: 392.1563.

For (11*R*,12*S*)-**7b**. [α]_D_^25^ = −75 (*c* 1.1, MeOH).

### *erythro*-13-Acetylmefloquine

*erythro*-Mefloquine hydrochloride (**1**·HCl, 27.3 g, 65.9 mmol) was suspended in isopropyl alcohol (300 mL), and solid K_2_CO_3_ (30.5 g, 221 mmol, 3.4 equiv) was added. The suspension was heated under reflux for 10 min in a heating mantle. After the mixture had reached room temperature, acetic anhydride (8.5 mL, 89.9 mmol, 1.37 equiv) was added dropwise, and the suspension was stirred overnight. Then, the mixture was concentrated, and the residue was suspended in distilled water (130 mL) for 1 h. The white crystalline solid was separated by filtration, washed with water, and air-dried. Residual inorganic material was removed by dissolving most of the sample in CHCl_3_ (300 mL) followed by filtration and evaporation to give 27.7 g of product, a quantitative yield as a white crystalline solid. Mp: 197–199 °C (lit.^11^ mp 202 °C).

### *threo*-13-Benzylmefloquine (**9**)

*threo*-Mefloquine hydrochloride^11^ (**8**·HCl, 2.75 g, 6.63 mmol) was suspended in dioxane (20 mL) and aqueous NaOH (10%, 7 mL) and stirred for 5 min. Benzyl bromide (0.89 mL, 7.49 mmol, 1.13 equiv) was added dropwise. The mixture was stirred for 8 days at room temperature. Next, the phases were separated, and the organic phase was washed with a saturated NaHCO_3_ solution. The aqueous layer was extracted with CH_2_Cl_2_ (3 × 10 mL). Organic phases were combined, dried over anhydrous Na_2_SO_4_, and evaporated. Crystallization from methanol (15 mL) gave a pure product (three crops, 2.68 g, 86%) as light yellow crystals. Mp = 121–123 °C. ^1^H NMR (400 MHz, CDCl_3_, TMS) δ = 8.05–8.08 (m, 2H), 7.71 (s, 1H), 7.36–7.45 (m, 6H), 5.46 (br s, 1H), 5.38 (d, *J* = 10.0 Hz, 1H), 3.96 (s, 2H), 3.10–3.17 (m, 1H), 2.86–2.95 (m, 2H), 1.60–1.84 (m, 4H), 1.46–1.49 (m, 1H), 0.99–1.03 (m, 1H) ppm. ^13^C{^1^H} NMR (100 MHz, CDCl_3_, TMS) δ = 152.0, 148.0 (q, *J* = 35.1 Hz), 144.3, 138.6, 129.47, 128.9, 128.8 (q, *J* = 5.5 Hz), 128.6, 127.92, 127.91, 126.7, 123.7 (q, *J* = 273.5 Hz), 121.3 (q, *J* = 275.3 Hz), 117.2 (q, *J* = 2.0 Hz), 68.5, 60.7, 57.2, 47.1, 20.8, 19.34, 19.31 ppm (one signal not observed due to overlap). ^19^F NMR (376 MHz, CDCl_3_, PhCF_3_) δ = −61.37 (s, 3F), −68.96 (s, 3F) ppm. HRMS (ESI-TOF) [C_24_H_22_F_6_N_2_O + H]^+^*m*/*z* calcd: 469.1709, found: 469.1711.

For (11*R*,12*R*)-**9**. Mp = 132–133.4 °C. [α]_D_^24^ = −83 (*c* 1, MeOH).

### *threo*-11-Azido-13-benzylmefloquine (**10**)

*threo*-13-Benzyl-mefloquine (**9**, 1.40 g, 3.00 mmol) was dissolved in dry THF (5 mL) and a solution of hydrazoic acid in benzene (1.50 M, 3.10 mL, 4.65 mmol, 1.55 equiv) was added. In another flask, triphenylphosphine (1.33 g, 5.08 mmol, 1.7 equiv) was dissolved in dry THF (10 mL) and the solution was cooled to 0 °C. Then, diisopropyl azadicarboxylate (DIAD, 1.00 mL, 5.08 mmol, 1.7 equiv) was added dropwise and soon a precipitate was formed. This suspension was added to the previously prepared solution of the substrate and hydrazoic acid, and stirred for 18 h at room temperature. Then, the mixture was concentrated and the crude product was purified using column chromatography on acidic aluminum oxide (CH_2_Cl_2_/EtOAc 2:3) giving 1.34 g of yellow crystalline solid (82%). Mp: 109.6–111.8 °C (EtOAc). ^1^H NMR (400 MHz, CDCl_3_, TMS) δ = 8.23 (d, *J* = 8.6 Hz, 1H), 8.15 (d, *J* = 7.2 Hz, 1H), 7.75 (s, 1H), 7.61 (t, *J* = 8.0 Hz, 1H), 7.27–7.42 (m, 5H), 5.53 (d, *J* = 9.4 Hz, 1H), 4.05 (d, *J* = 13.3 Hz, 1H), 4.00 (d, *J* = 13.3 Hz, 1H), 3.30–3.33 (m, 1H), 3.10–3.15 (m, 1H), 2.77–2.80 (m, 1H), 1.48–1.66 (m, 5H), 0.88–0.91 (m, 1H) ppm. ^13^C{^1^H} NMR (151 MHz, CDCl_3_, TMS) δ = 148.2 (q, *J* = 35.5 Hz), 147.5, 144.5, 139.5, 129.8 (q, *J* = 30.3 Hz), 129.2 (q, *J* = 5.3 Hz), 128.9, 128.55, 128.45 127.4, 127.3, 127.2, 123.6 (q, *J* = 273.7 Hz), 121.2 (q, *J* = 275.6 Hz), 117.3, 63.6, 61.5, 58.0, 47.3, 23.1, 21.2, 21.1 ppm. ^19^F NMR (376 MHz, CDCl_3_, PhCF_3_) δ = −61.34 (s, 3F), −68.90 (s, 3F) ppm. HRMS (ESI-TOF) [C_24_H_21_F_6_N_5_ + H]^+^*m*/*z* calcd: 494.1774, found: 494.1773

For (11*R*,12*R*)-**10**. Yellow amorphous solid, [α]_D_^26^ = −67 (*c* 0.9, MeOH).

### *threo*-11-Amino-13-benzylmefloquine (**11**)

*threo*-11-Azido-13-benzylmefloquine (**10**, 48.5 mg, 0.0984 mmol) was dissolved in methanol (2.5 mL). Next, palladium on carbon was added (5%, 5.0 mg, 2.5 mol %) was added. The reaction vessel was loaded with hydrogen (6.0 bar), and the mixture was stirred for 24 h. Then, the mixture was filtered and the solvent was removed. The product was obtained by flash chromatography on silica gel (hexane/EtOAc 1:0 to 2:3 gradient) as a pale amorphous solid (30 mg, 65% yield). ^1^H NMR (400 MHz, CDCl_3_, TMS) δ = 8.53 (d, *J* = 8.6 Hz, 1H), 8.13 (d, *J* = 7.2 Hz, 1H), 7.97 (s, 1H), 7.60 (t, *J* = 8.0 Hz, 1H), 7.30–7.44 (m, 5H), 5.05 (d, *J* = 9.9 Hz, 1H), 3.95 (s, 2H), 2.99–3.08 (m, 2H), 2.74–2.78 (m, 1H), 2.14 (br s, 2H), 1.36–1.73 (m, 5H), 0.93–0.99 (m, 1H) ppm. ^13^C{^1^H} NMR (100 MHz, CDCl_3_, TMS) δ = 154.5, 148.3 (q, *J* = 35.0 Hz), 144.3, 139.8, 129.5 (q, *J* = 30.0 Hz), 129.0, 128.8 (q, *J* = 5.5 Hz), 128.62, 128.56, 127.4, 126.6, 123.8 (q, *J* = 273.6 Hz), 121.4 (q, *J* = 275.5 Hz), 117.2 (q, *J* = 2.0 Hz), 63.6, 55.6, 52.1, 47.0, 22.1, 21.0, 19.2 ppm (one signal not observed due to overlap). ^19^F NMR (376 MHz, CDCl_3_, PhCF_3_) δ = −61.32 (s, 3F), −68.88 (s, 3F) ppm. HRMS (ESI-TOF) [C_24_H_23_F_6_N_3_ + H]^+^*m*/*z* calcd: 468.1869, found: 468.1869.

For (11*R*,12*R*)-**11**. [α]_D_^25^ = −57 (*c* 1, MeOH).

### *threo*-11-Aminomefloquine (**12**)

*threo*-11-Azido-13-benzyl-mefloquine (**10**, 103 mg, 0.209 mmol) was dissolved in a mixture of methanol (17 mL) and TFA (0.80 mL). Palladium on carbon (5%, 8 mg, 2 mol %) was added, the reaction vessel was loaded with hydrogen (6.0 bar), and the mixture was stirred for 18 h. Then the catalyst was filtered off, and the solution was concentrated in vacuo. The crude product was diluted with ethyl acetate (25 mL) and washed with saturated aqueous NaHCO_3_ (10 mL). The organic phase was dried over anhydrous Na_2_SO_4_, and the solvent was evaporated. Crude product was purified using column chromatography on basic aluminum oxide (EtOAc/MeOH 1:0 to 0:1 gradient). The product (54 mg, 64% yield) was obtained as a light orange amorphous solid. ^1^H NMR (400 MHz, CDCl_3_, TMS) δ = 8.42 (d, *J* = 8.6 Hz, 1H), 8.19 (d, *J* = 7.2 Hz, 1H), 7.97 (s, 1H), 7.75 (t, *J* = 7.9 Hz, 1H), 4.68 (d, *J* = 6.5 Hz, 1H), 3.12–3.15 (m, 1H), 2.78–2.83 (m, 1H), 2.58 (td, *J* = 11.9, 2.8 Hz, 1H), 2.03 (br s, 3H), 1.74–1.77 (m, 1H), 1.59–1.62 (m, 1H), 1.25–1.47 (m, 4H) ppm. ^13^C NMR (151 MHz, CDCl_3_, TMS) δ = 153.9, 148.4 (q, *J* = 35.0 Hz), 144.1, 129.7 (q, *J* = 30.1 Hz), 129.0 (q, *J* = 5.5 Hz), 127.8, 127.7, 127.2, 123.7 (q, *J* = 273.7 Hz), 121.4 (q, *J* = 275.5 Hz), 115.4 (q, *J* = 1.8 Hz), 62.2, 55.8, 47.0, 30.3, 26.2, 24.7 ppm. ^19^F NMR (376 MHz, CDCl_3_, PhCF_3_) δ = −61.31 (s, 3F), −68.85 (s, 3F) ppm. HRMS (ESI-TOF) [C_17_H_17_F_6_N_3_ + H]^+^*m*/*z* calcd: 378.1399, found: 378.1398.

For (11*R*,12*R*)-**12**. [α]_D_^20^ = −22 (*c* 1, MeOH).

### *threo*-11-Azidomefloquine (**13**)

*erythro*-11-Azidomefloquine hydrazoic acid salt (**3**·HN_3_ 65 mg, 0.15 mmol) was dissolved in methanol (1.6 mL) and sodium hydroxide (25 mg, 0.56 mmol, 3.7 equiv) was added. The mixture was briefly stirred until dissolution and stored at room temperature for 72 h. The solution was diluted with saturated aqueous NaHCO_3_ (25 mL) and CH_2_Cl_2_ (25 mL), washed with saturated NaCl (15 mL), dried over K_2_CO_3_, and evaporated. Chromatography on silica gel (CH_2_Cl_2_/MeOH 20:1) gave 12 mg of white solid (20% yield, dr 95:5). ^1^H NMR (600 MHz, CDCl_3_, TMS) δ = 8.42 (d, *J* = 8.9 Hz, 1H) 8.23 (d, *J* = 7.1 Hz, 1H), 7.88 (s, 1H), 7.80 (dd, *J* = 8.9, 7.1 Hz, 1H), 5.14 (d, *J* = 8.2 Hz, 1H), 3.13–3.18 (m, 1H), 2.89 (ddd, *J* = 10.6, 8.9, 2.7 Hz, 1H), 2.63 (td, *J* = 12.1, 2.9 Hz, 1H), 2.0 (br., 1H), 1.68–1.73 (m, 1H), 1.58–1.63 (m, 1H), 1.39–1.47 (m, 1H), 1.13–1.26 (m, 2H), 1.06–1.10 (m, 1H) ppm. ^13^C{^1^H} NMR (151 MHz, CDCl_3_, TMS) δ = 148.5 (q, *J* = 35.7 Hz), 146.6, 144.4, 129.8 (q, *J* = 30.8 Hz), 129.4 (q, *J* = 5.3 Hz), 127.8, 127.7, 127.5, 123.5 (q, *J* = 273.6 Hz), 121.2 (q, *J* = 275.5 Hz), 117.0, 68.1, 60.8, 46.8, 29.7, 25.8, 24.3 ppm. HRMS (ESI-TOF) [C_17_H_15_F_6_N_5_ + H]^+^*m*/*z* calcd: 404.1299, found:404.1306

### *erythro*-Hexahydro-1-(2,8-Bis(trifluoromethyl)-4-quinolinyl)-imidazo-[1,5-*a*]pyridin-3(2*H*)-one (**14**)

*erythro*-11-Aminomefloquine (**4**, 50 mg, 0.14 mmol) was dissolved in CH_2_Cl_2_ (4 mL), and *N,N*-diisopropylethylamine (50 μL, 0.29 mmol, 2 equiv) and phosgene solution (20% in toluene, 15 μL, ca. 1.1 equiv) were added. The mixture was stirred for 18 h, evaporated, and filtered through a plug of silica gel with EtOAc. The mixture was evaporated and the residue triturated with chloroform (1.5 mL). Obtained 31 mg of white crystalline product (58%). ^1^H NMR (600 MHz, DMSO-*d*_6_, TMS) δ = 8.65 (d, *J* = 8.5 Hz, 1H), 8.40 (d, *J* = 7.3 Hz, 1H), 8.01 (s, 1H), 7.97 (dd, *J* = 8.5, 7.3 Hz, 1H), 7.16 (d, *J* = 1.2 Hz, 1H), 5.80 (dd, *J* = 8.5, 1.2 Hz, 1H), 4.20 (ddd, *J* = 12.0, 8.8, 3.4 Hz, 1H), 3.75–3.79 (m, 1H), 2.72 (td, *J* = 12.9, 3.4 Hz, 1H), 1.52–1.56 (m, 1H), 1.41–1.46 (m, 1H), 1.19–1.27 (m, 1H), 1.03–1.14 (m, 1H), 0.62 (qd, *J* = 12.5, 3.5 Hz, 1H), 0.50–0.54 (m, 1H) ppm. ^13^C{^1^H} NMR (151 MHz, DMSO-*d*_6_, TMS) δ = 160.2, 150.1, 147.3 (q, *J* = 34.5 Hz), 143.0, 130.5 (q, *J* = 5.3 Hz), 129.4, 129.0, 127.8, 127.6 (q, *J* = 29.7 Hz), 124.1 (q, *J* = 273.6 Hz), 121.7 (q, *J* = 275.4 Hz), 115.6, 57.6, 53.7, 41.0, 26.3, 24.5, 23.2 ppm. ^19^F NMR (400 MHz, DMSO-*d*_6_, PhCF_3_) δ = −61.40 (s, 3F), −69.22 (s, 3F) ppm. HRMS (ESI-TOF) [C_18_H_15_F_6_N_3_O + H]^+^*m*/*z* calcd: 404.1201, found: 404.1191.

### *threo*-Hexahydro-1-(2,8-Bis(trifluoromethyl)-4-quinolinyl)-imidazo-[1,5-*a*]pyridin-3(2*H*)-one (**15**)

*threo*-11-Aminomefloquine (**12**, 42.5 mg, 0.113 mmol) was dissolved in acetonitrile (3.5 mL). Next, 1,1′-carbonyldiimidazole (CDI, 20.3 mg, 0.125 mmol, 1.1 equiv) was added. After 14 days, the mixture was purified on a silica gel column with ethyl acetate. The product was isolated as a white, amorphous solid (31.0 mg, 66% yield). ^1^H NMR (600 MHz, CDCl_3_, TMS) δ = 8.24 (d, *J* = 8.6 Hz, 1H), 8.21 (d, *J* = 7.3 Hz, 1H), 8.08 (s, 1H), 7.80 (t, *J* = 8.0 Hz, 1H), 6.54 (s, 1H), 5.21 (d, *J* = 5.4 Hz, 1H), 3.88 (dd, *J* = 13.3, 4.5 Hz, 1H), 3.34–3.37 (m, 1H), 2.65 (td, *J* = 12.9, 3.2 Hz, 1H), 2.00–2.02 (m, 1H), 1.94–1.96 (m, 1H), 1.72–1.79 (m, 1H), 1.62–1.64 (m, 1H), 1.41–1.49 (m, 1H), 1.31–1.39 (m, 1H) ppm. ^13^C{^1^H} NMR (151 MHz, CDCl_3_, TMS) δ = 160.7, 150.1, 148.8 (q, *J* = 35.4 Hz), 144.1, 130.0 (q, *J* = 30.3 Hz), 129.1 (q, *J* = 5.3 Hz), 127.7, 127.1, 126.6, 123.5 (q, *J* = 273.7 Hz), 121.2 (q, *J* = 275.5 Hz), 114.9, 63.5, 56.7, 41.0, 30.9, 24.5, 23.4 ppm. ^19^F NMR (376 MHz, CDCl_3_, PhCF_3_) δ = −61.37 (s, 3F), −68.88 (s, 3F) ppm. HRMS (ESI-TOF) [C_18_H_15_F_6_N_3_O + H]^+^*m*/*z* calcd: 404.1192, found: 404.1191.

### 13-Benyzl-11-chloromefloquine Hydrochloride

*erythro*-13-Benzyl mefloquine (**5a**, 1.39 g, 2.96 mmol) was dissolved in thionyl chloride (6 mL) and heated in an oil bath at 65 °C for 18 h. Then the mixture was evaporated to give 1.47 g of light pink crystalline solid (95%). Mp: 151–159 °C. HRMS (ESI-TOF) [C_24_H_21_ClF_6_N_2_ + H]^+^*m*/*z* calcd: 487.1370, found: 487.1382.

Identical results were obtained starting from *threo*-13-benzylmefloquine (**9**, 109 mg, 0.23 mmol) and 2 mL of thionyl chloride.

### Asymmetric Michael Reaction^19^

Catalyst (15.1 mg for **4**, 0.040 mmol, 10 mol %) was dissolved in nitromethane (1 mL). Next, benzoic acid (4.88 mg, 0.040 mmol, 10 mol %) was added, and the mixture was stirred until dissolution. Finally, 2-cyclohexen-1-one (38.7 μL, 0.40 mmol) was slowly added via syringe. The mixture was stirred in an oil bath at 40 °C for 4 days and filtered through a silica gel plug (4 g) with ethyl acetate. The solvent was evaporated, and the HPLC analysis of the crude product was performed (IC-3 column, 4.6 × 250 mm, hexane/2-propanol, 6/4, flow rate: 0.8 mL/min, λ = 220 nm); *t*_R_ = 24 min (*S* enantiomer) and 31 min (*R* enantiomer).

## Supporting Information Available

Plots of NMR spectra, assignment of NMR signals for compounds **3b, 14**, and **15**, chiral HPLC chromatograms of mefloquine derivatives and catalytic products, and computational data for compounds **14** and **15** (PDF)
FAIR data, including the primary NMR FID files, for compounds **2**–**7b** and **9**–**16** (ZIP)
