# Supplementary material for: Chiral Vicinal Diamines Derived from Mefloquine
Source: J Org Chem. 2021 Jul 27;86(15):10654–64. doi: 10.1021/acs.joc.1c01316 (PMC8389910; doi:10.1021/acs.joc.1c01316)
Supplement: Supplementary file 1 — jo1c01316_si_001.pdf [file jo1c01316_si_001.pdf]

Supporting Information

Chiral vicinal diamines derived from Mefloquine

Dawid J. Kucharski,[a] Rafał Kowalczyk,[b] and Przemysław J. Boratyński [a*]

[a] *Department of Organic and Medicinal Chemistry, Wrocław University of Technology, Wyb. Wyspiańskiego 26, Wrocław, 50-370 Poland*

[b] *Department of Bioorganic chemistry, Wrocław University of Technology, Wyb. Wyspiańskiego 26, Wrocław, 50-370 Poland*

*e-mail: przemyslaw.boratynski@pwr.edu.pl

Table of contents

Spectral assignment for compounds **3b**, **14**, and **15** S-2

Plots of NMR spectra S-3

Spectral data for 11-chloro-13-benzyl-mefloquine hydrochloride S-41

Chiral HPLC chromatogram plots S-45

Scheme for resolution of mefloquine (±-**1**) S-50

Computational data listings for compounds **14** and **15** S-51

Spectral assignment for compounds 3b, 14, and 15

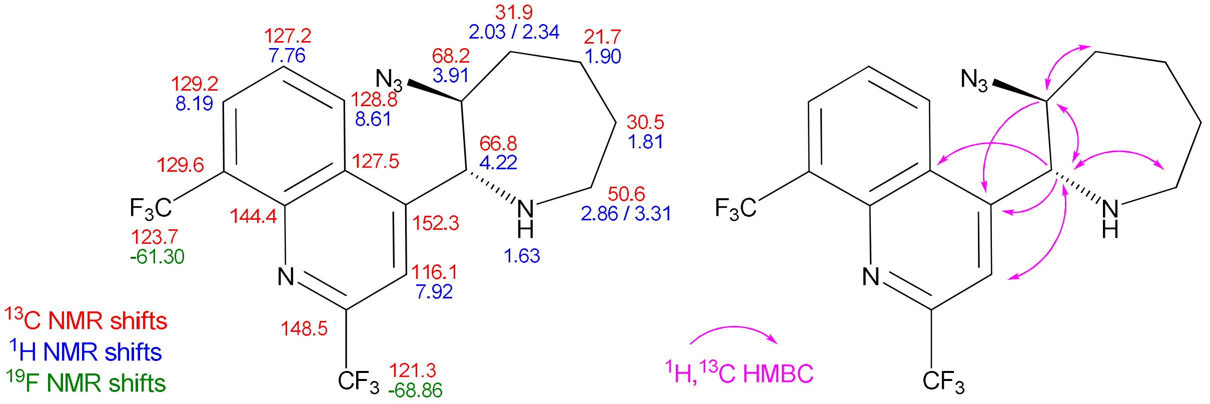

Figure S1. Spectral assignment for **3b** and diagnostic ^{1}H,^{13}C-HMBC interactions for aliphatic methine (CH) groups.

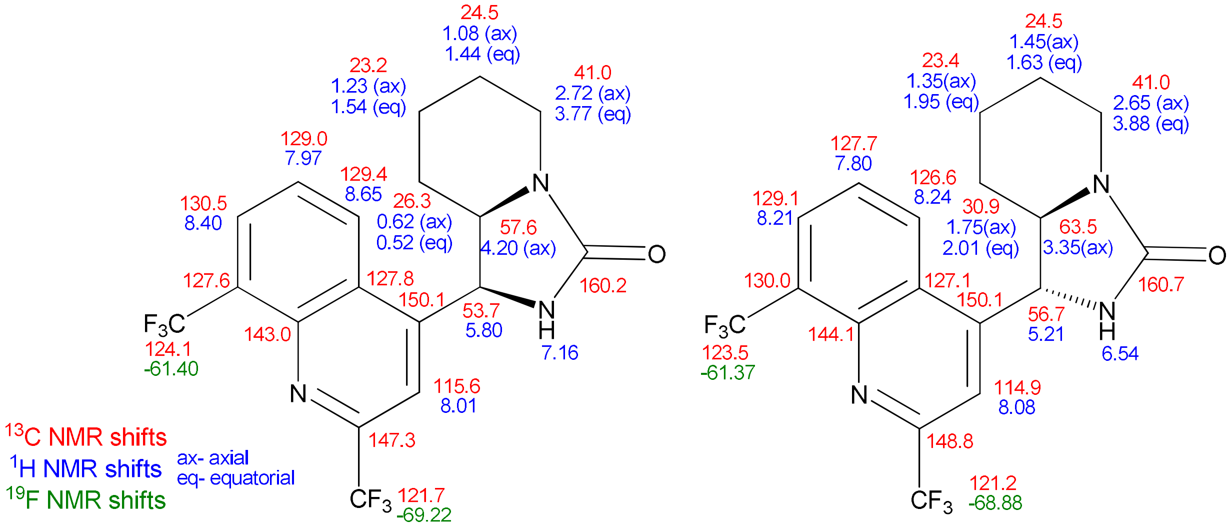

Figure S2 Spectral assignment for cyclic urea derived from *erythro*-11-aminomefloquine (**14**, left) and *threo*-11-aminomefloquine (**15**, right).

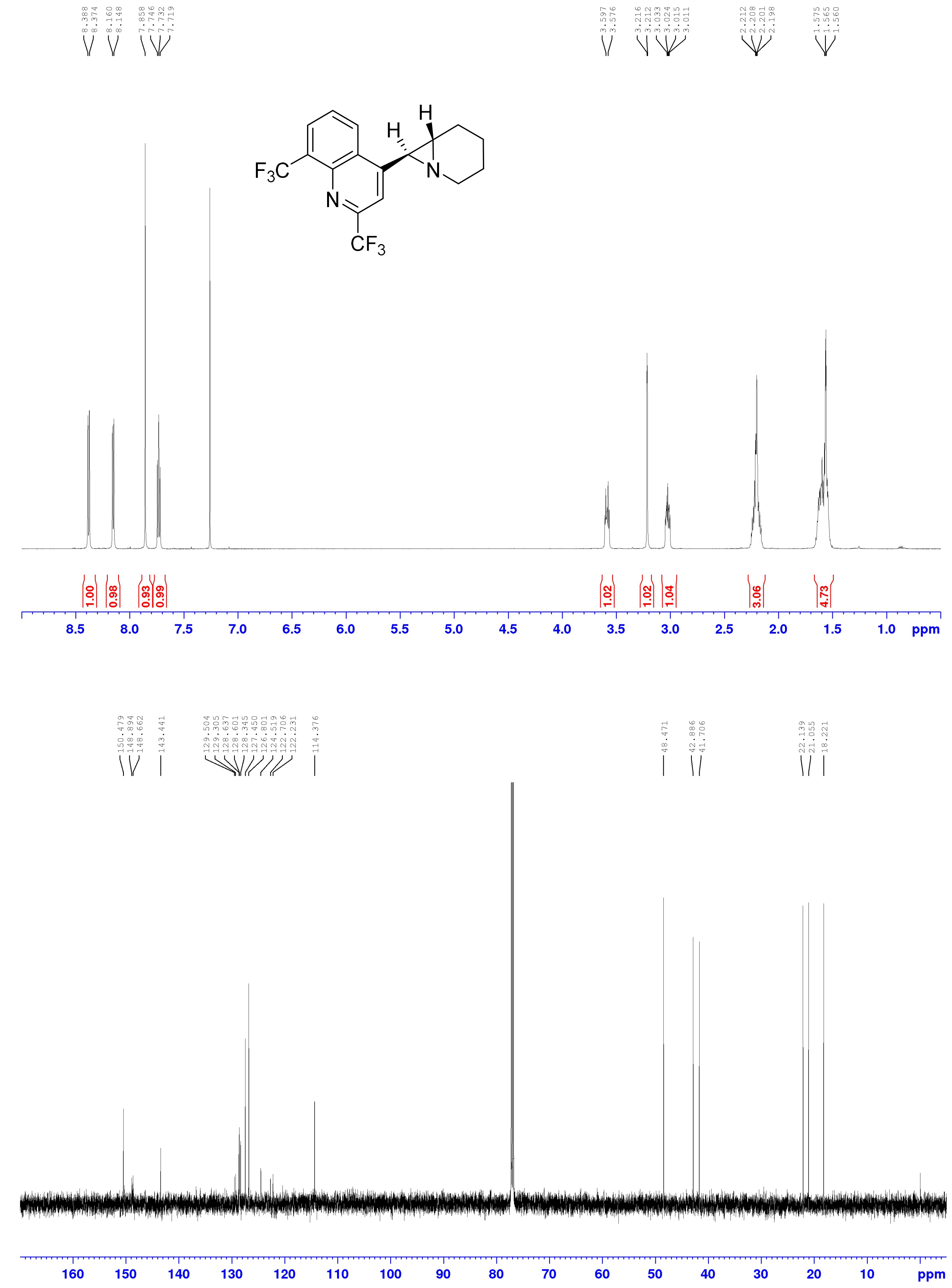

Figure S3. ^{1}H (600 MHz) and $^{13}C\{^{1}H\}$ NMR (151 MHz) spectra for **2** in $CDCl_3$+TMS

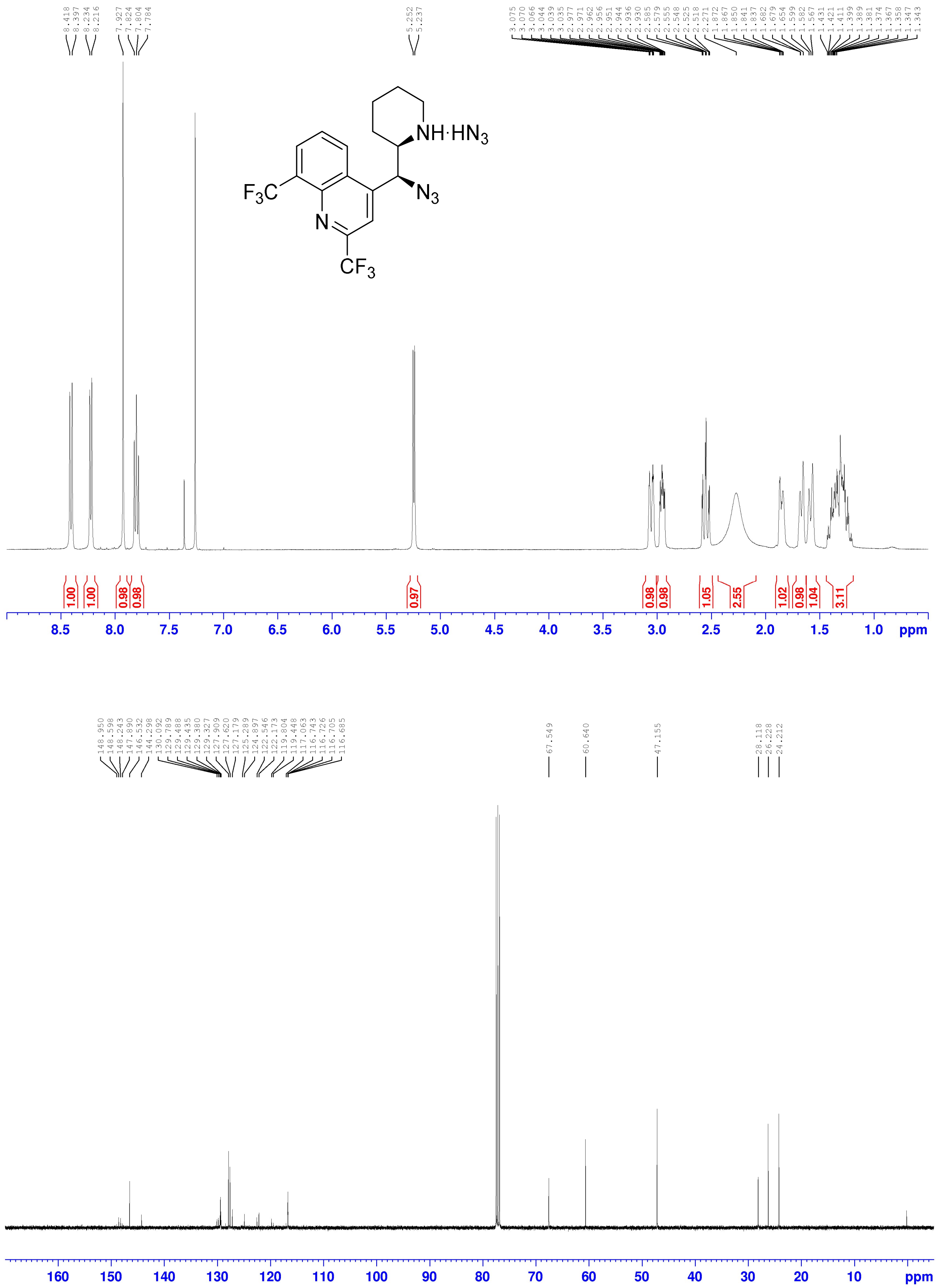

Figure S4. ^{1}H (400 MHz) and $^{13}C\{^{1}H\}$ NMR (100 MHz) spectra for **3**·HN_3 in $CDCl_3$+TMS

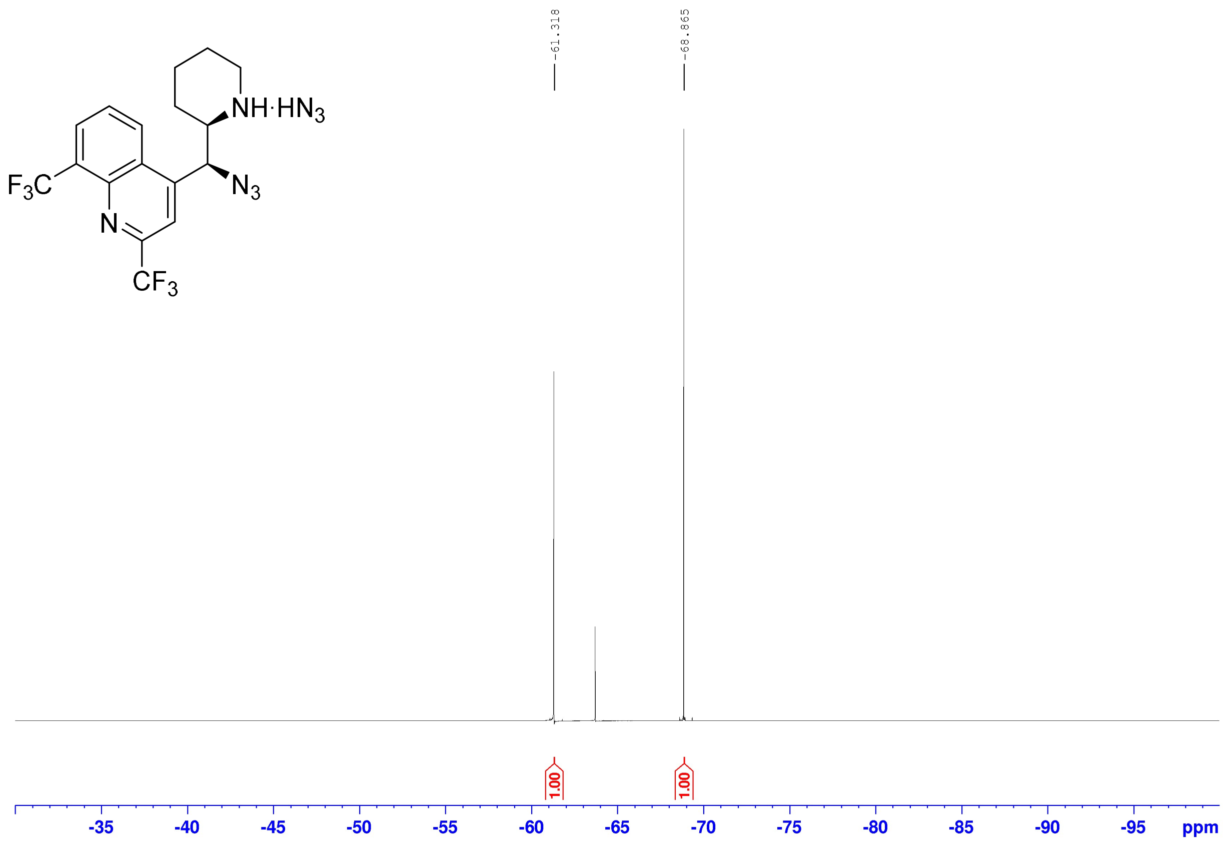

Figure S5. ^{19}F NMR (376 MHz) spectrum for **3**·HN_3 in $CDCl_3$+$PhCF_3$ ($\delta_F = -63.72$ ppm)

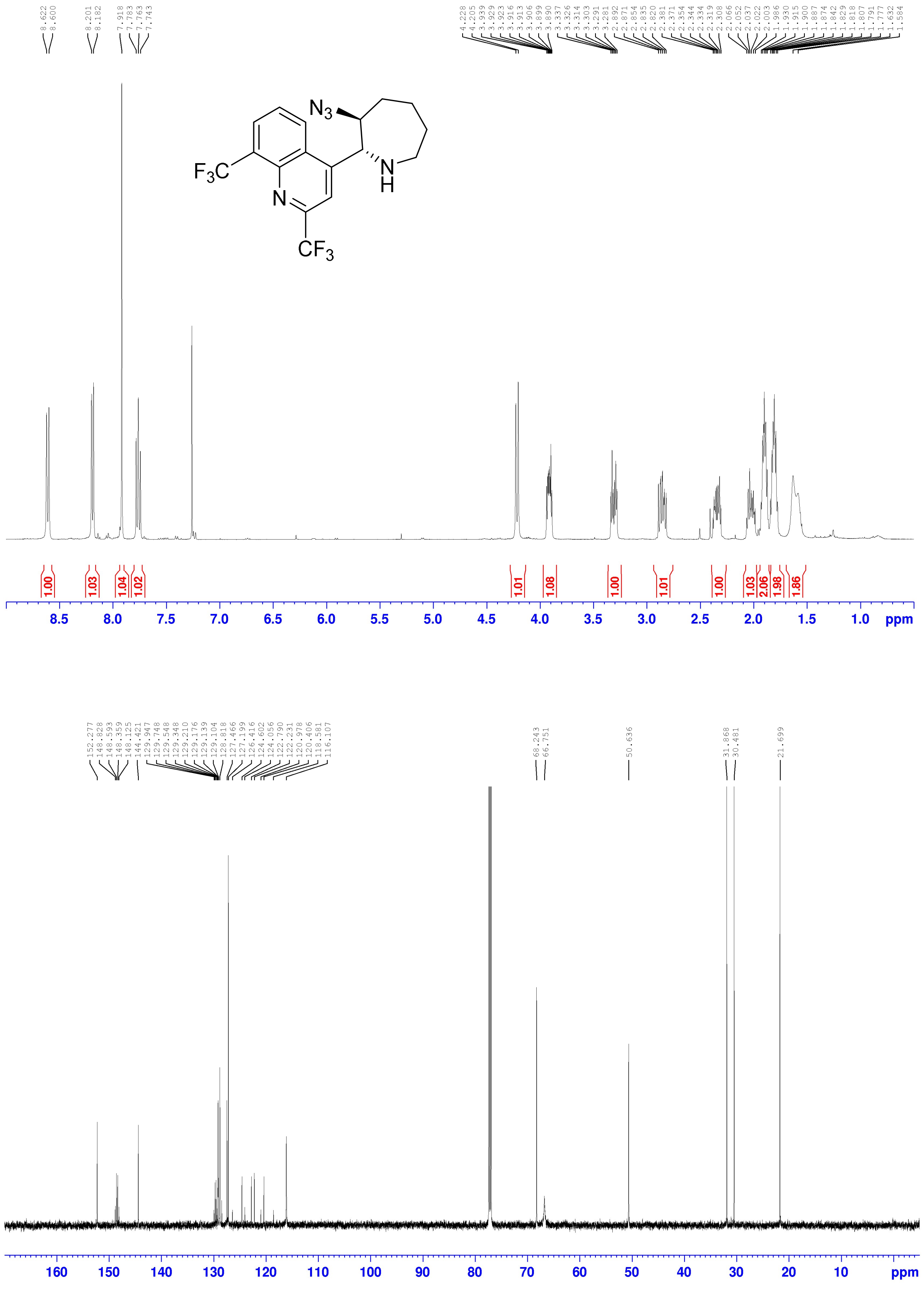

Figure S6. ^{1}H (400 MHz) and $^{13}C\{^{1}H\}$ NMR (151 MHz) spectra for **3b** in $CDCl_3$+TMS

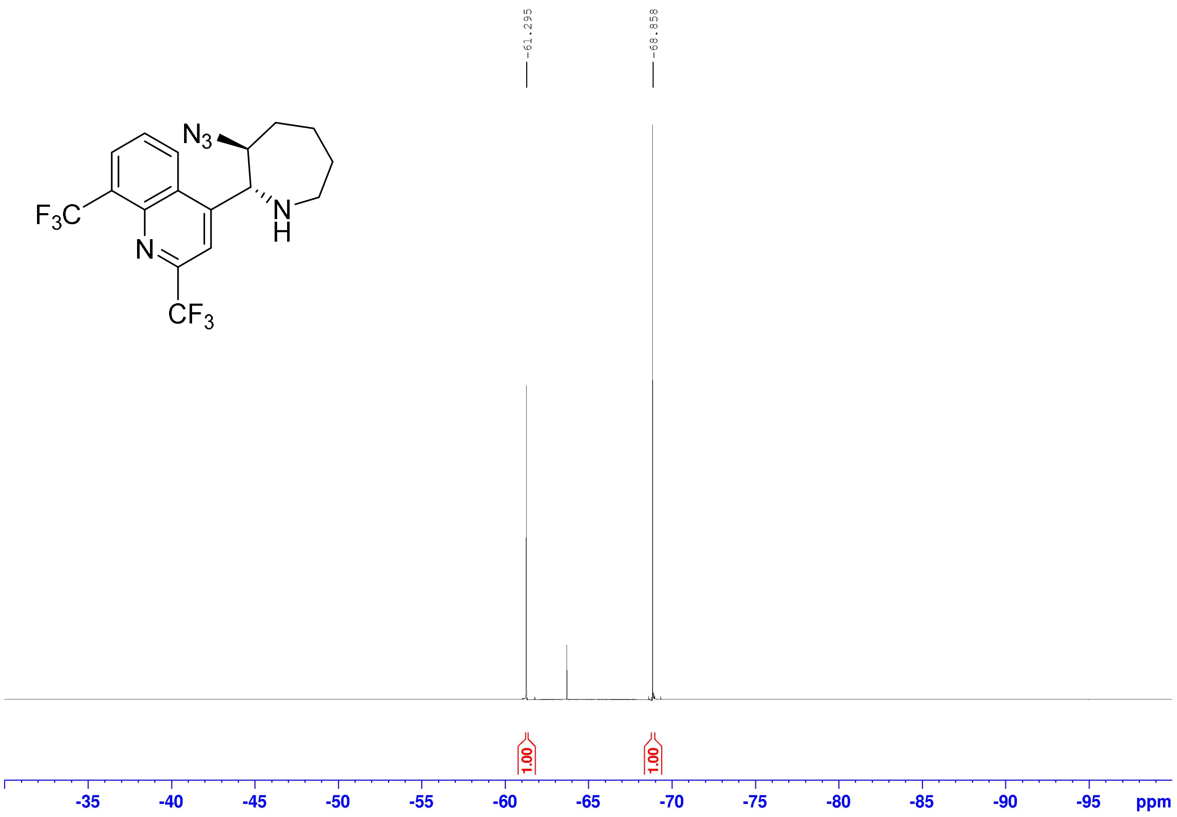

Figure S7. ^{19}F NMR (376 MHz) spectrum for **3b** in $CDCl_3$+$PhCF_3$ ($\delta_F = -63.72$ ppm)

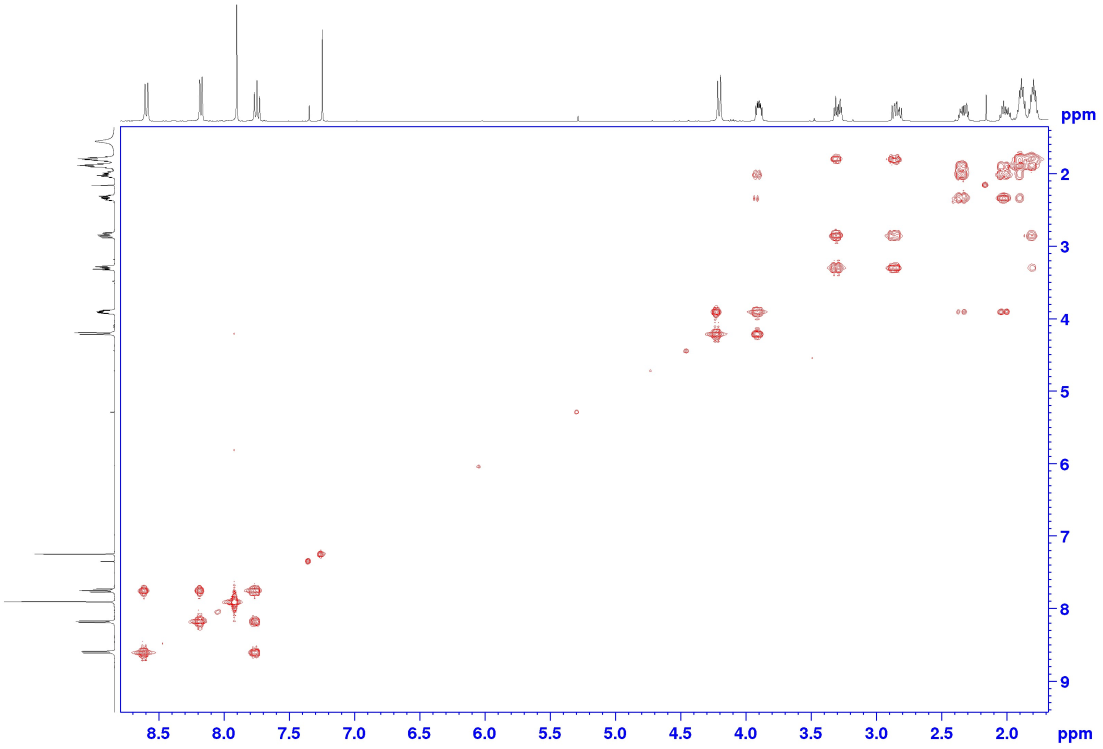

Figure S8. ^{1}H COSY experiment (600 MHz) for **3b** in $CDCl_3$. For spectral assignment, see Figure S1.

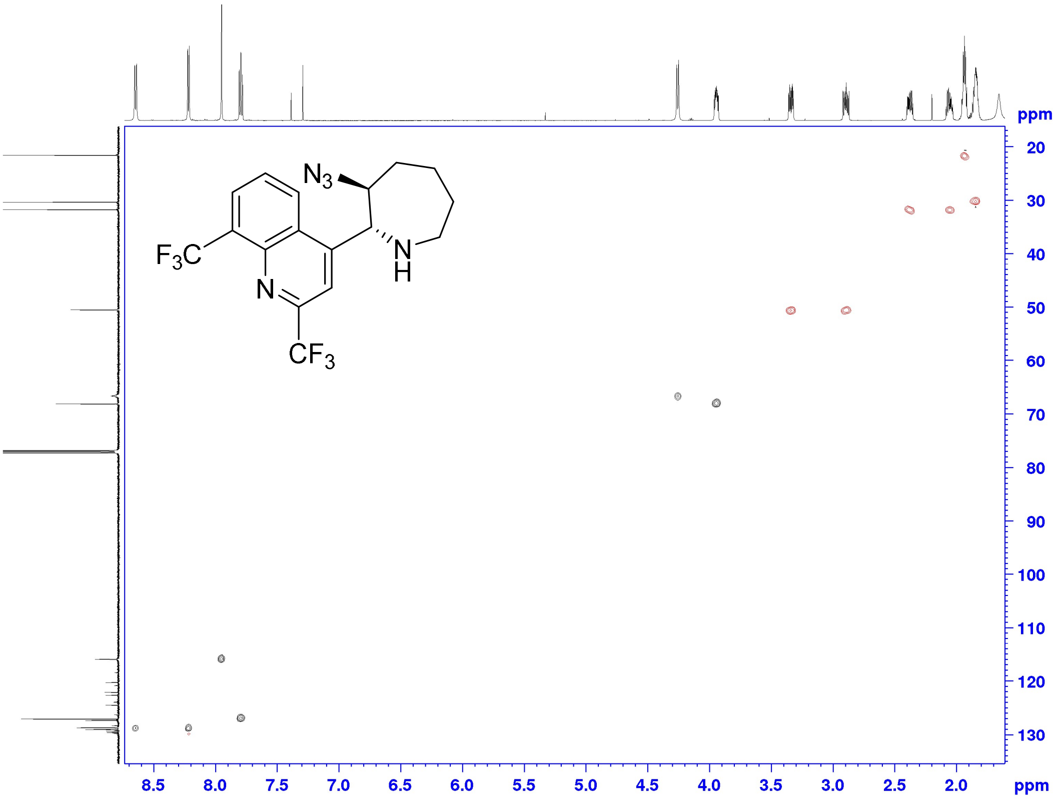

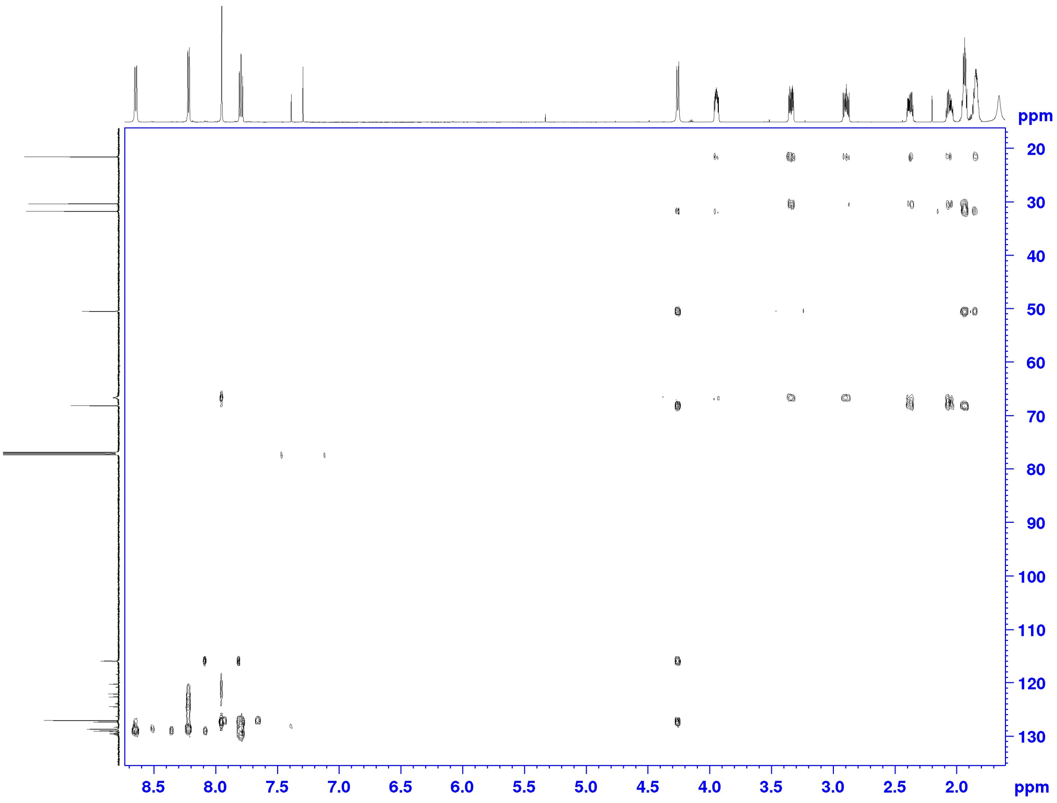

Figure S9. ^{1}H,^{13}C multiplicity-resolved HSQC (CH_2 -red, CH/CH_3 -black; top) and HMBC (bottom) experiments for **3b** in $CDCl_3$. For spectral assignment, see Figure S1.

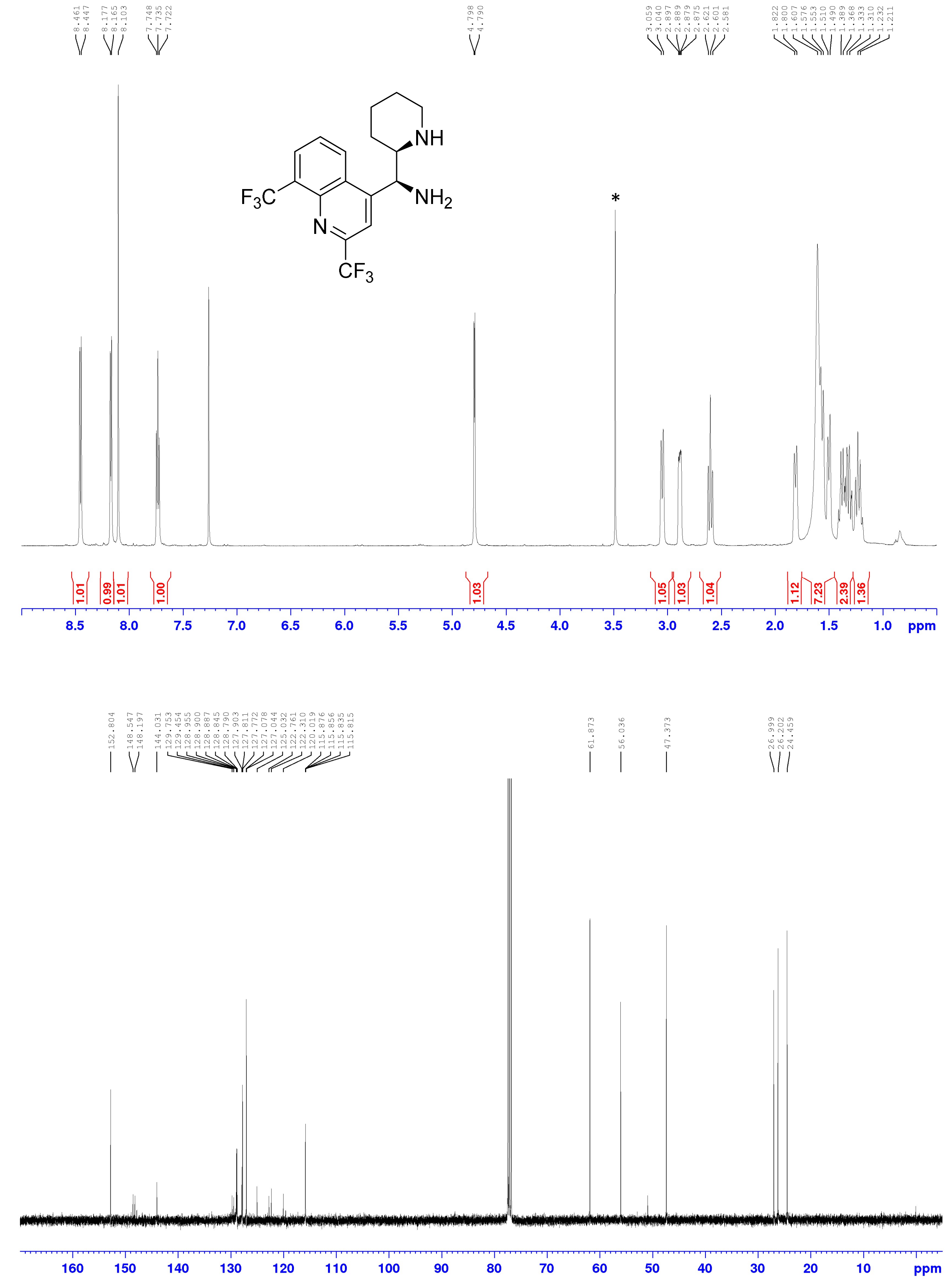

Figure S10. ¹H (400 MHz) and ¹³C{¹H} NMR (100 MHz) spectra for **4** in $CDCl_3$+TMS (* sample contains residue of MeOH from crystalline solvate)

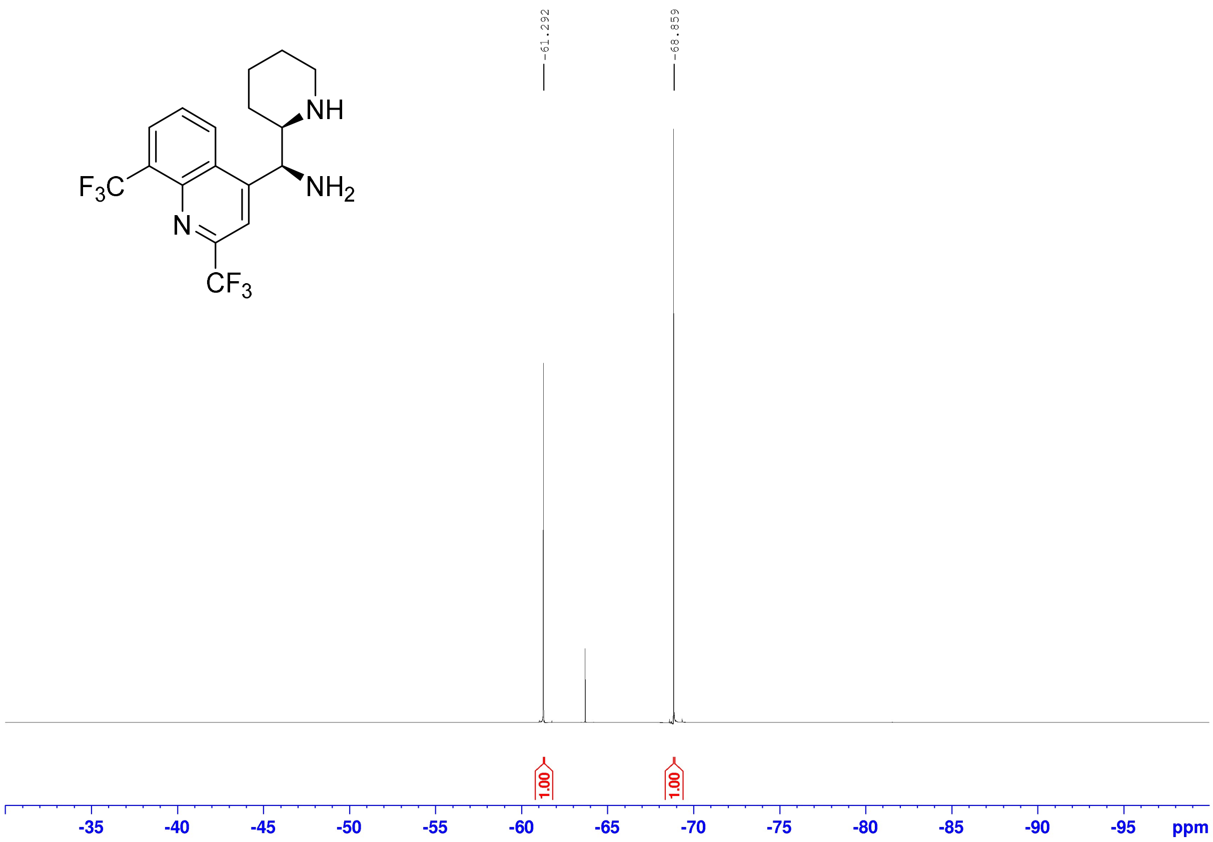

Figure S11. ^{19}F NMR (376 MHz) spectrum for **4** in $CDCl_3$+$PhCF_3$ ($\delta_F = -63.72$ ppm)

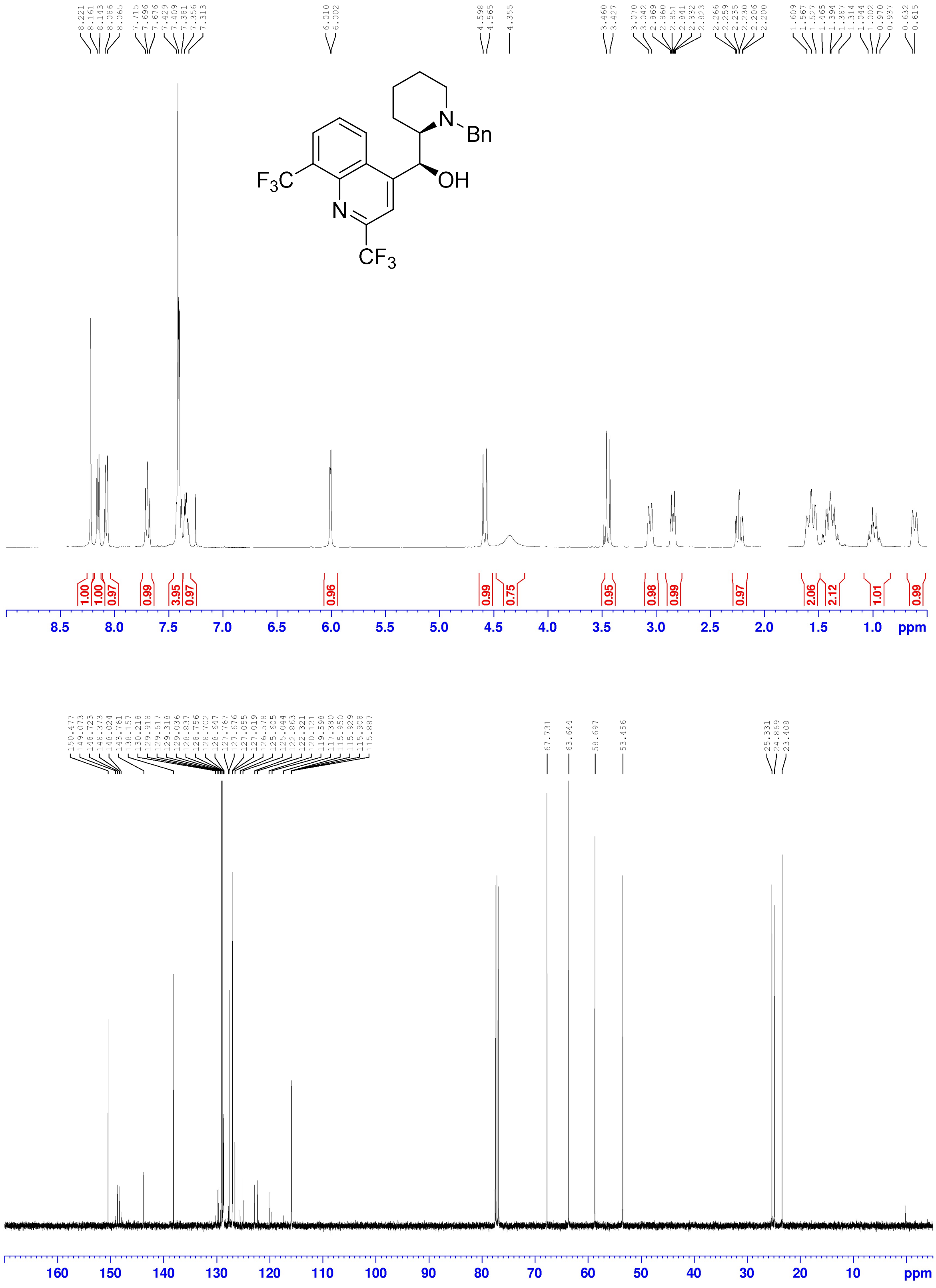

Figure S12. ^{1}H (400 MHz) and ^{13}C{^{1}H} NMR (100 MHz) spectra for **5a** in $CDCl_3$+TMS

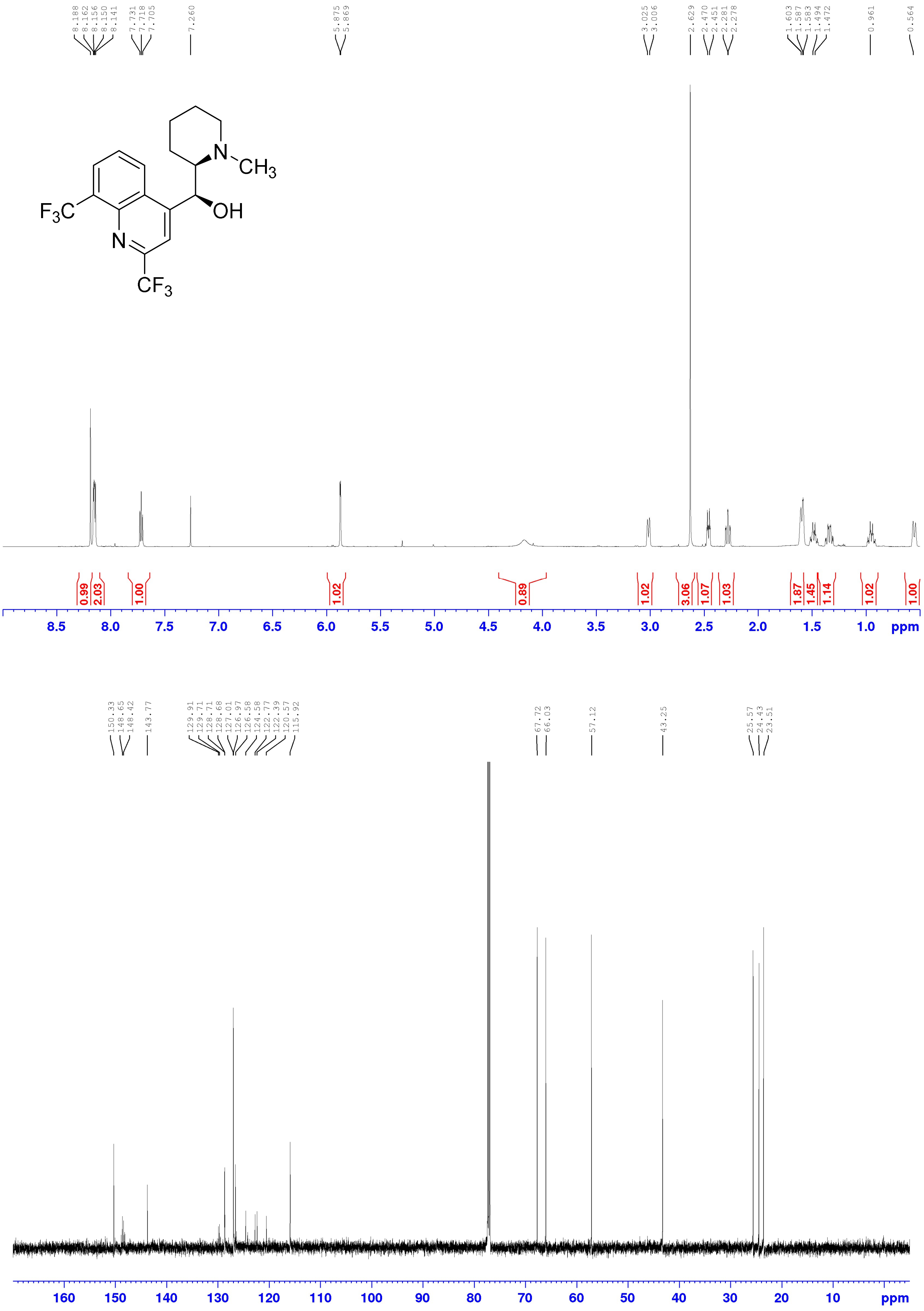

Figure S13. ^{1}H (600 MHz) and $^{13}C\{^{1}H\}$ NMR (151 MHz) spectra for **5b** in $CDCl_3$+TMS

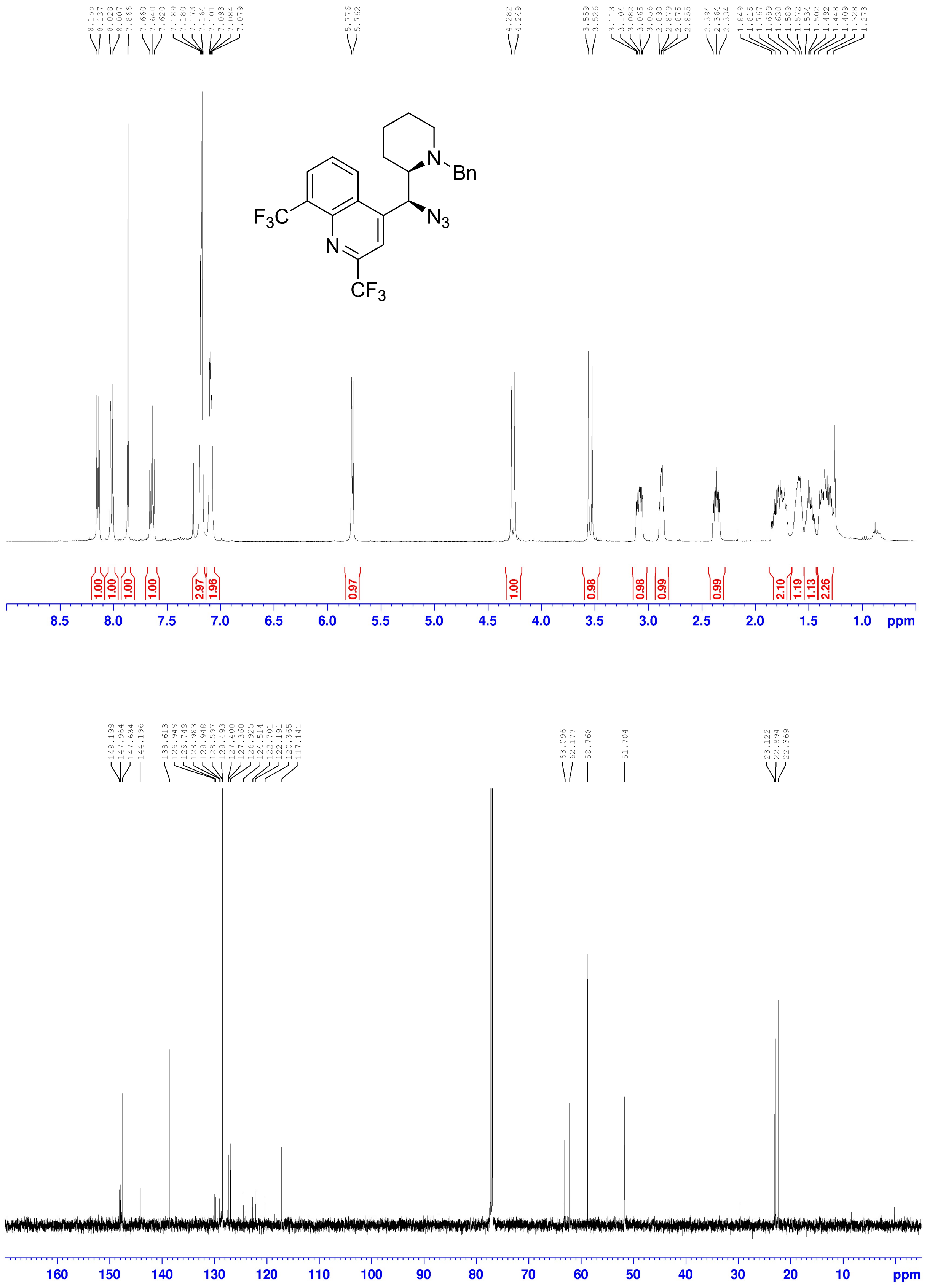

Figure S14. ^{1}H (600 MHz) and ^{13}C{^{1}H} NMR (151 MHz) spectra for **6a** in $CDCl_3$+TMS

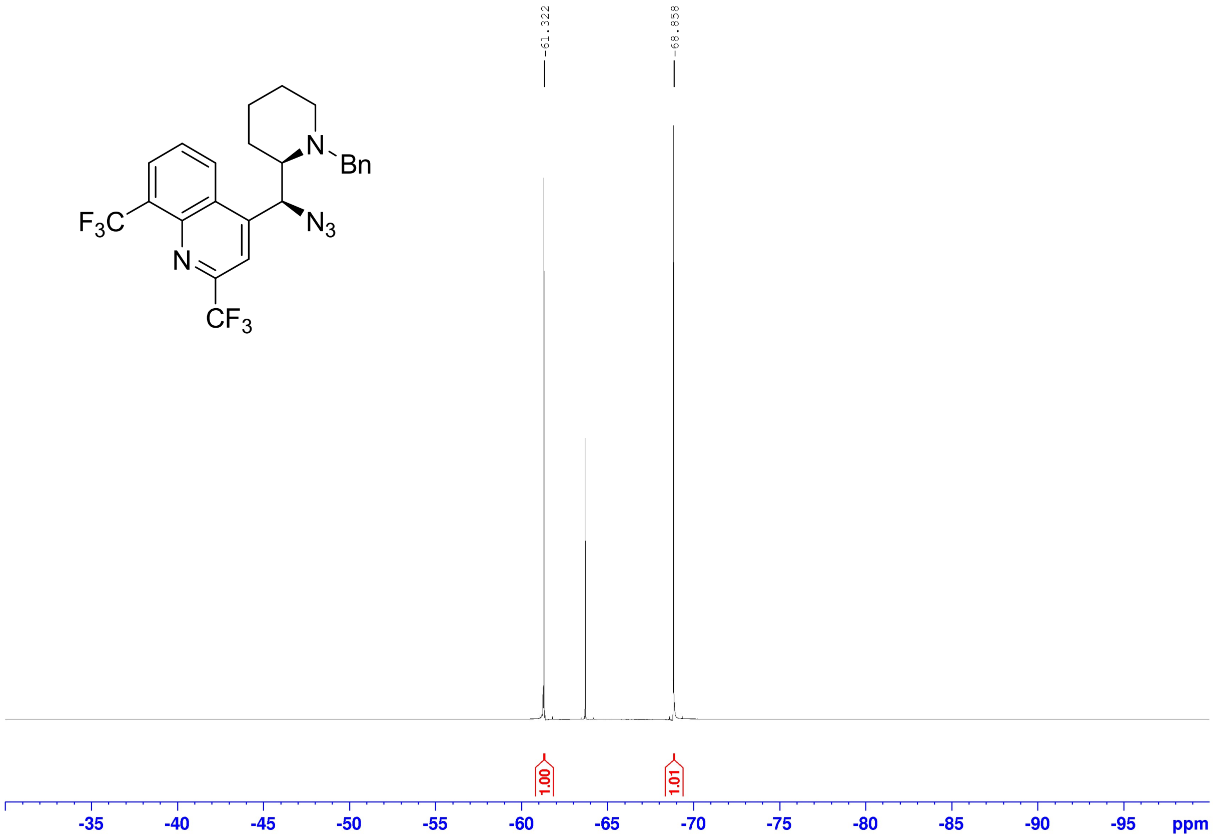

Figure S15. ^{19}F NMR (376 MHz) spectrum for **6a** in $CDCl_3$+$PhCF_3$ (δ_F = −63.72 ppm)

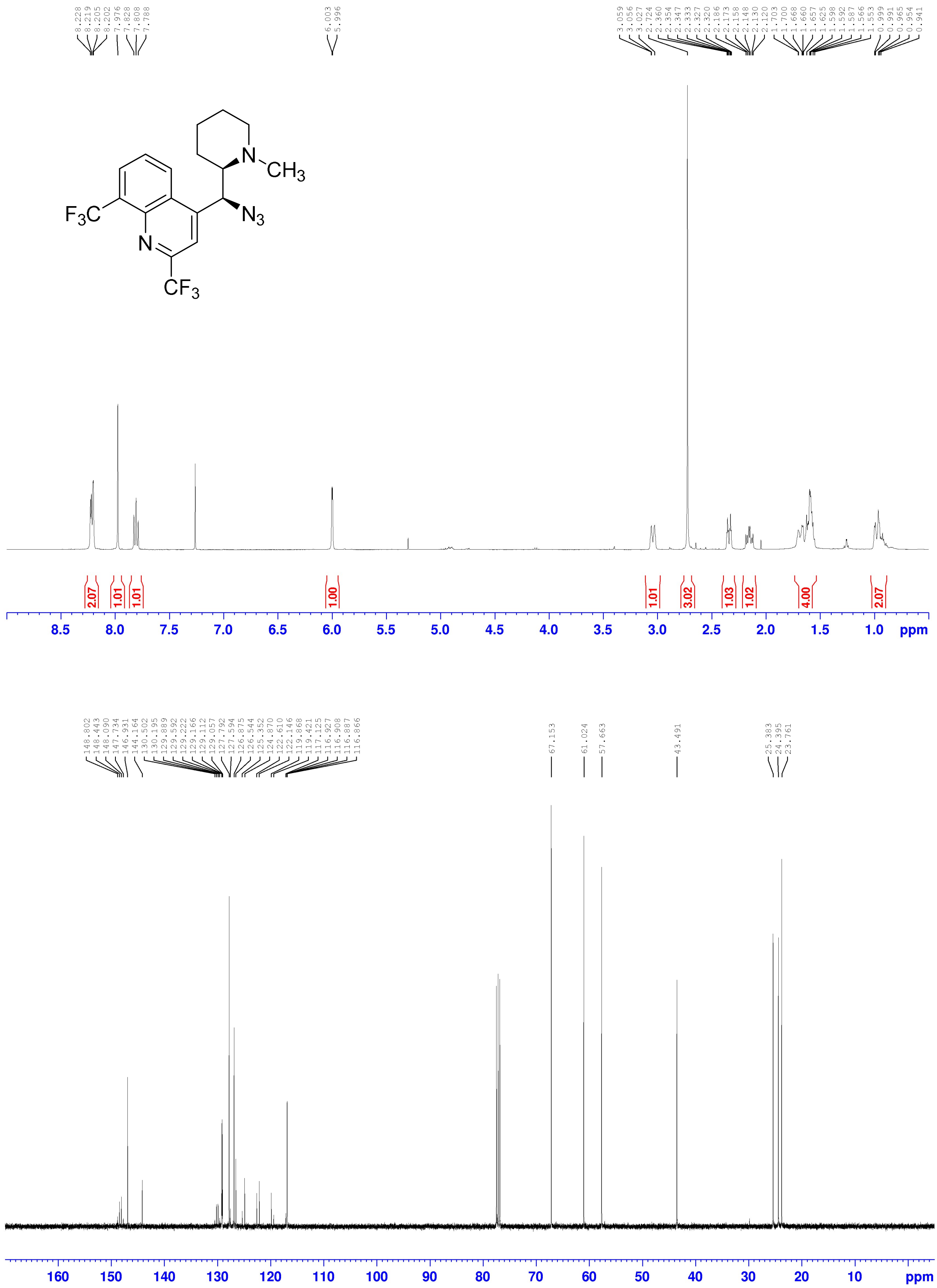

Figure S16. ¹H (400 MHz) and ¹³C{¹H} NMR (100 MHz) spectra for **6b** in $CDCl_3$+TMS

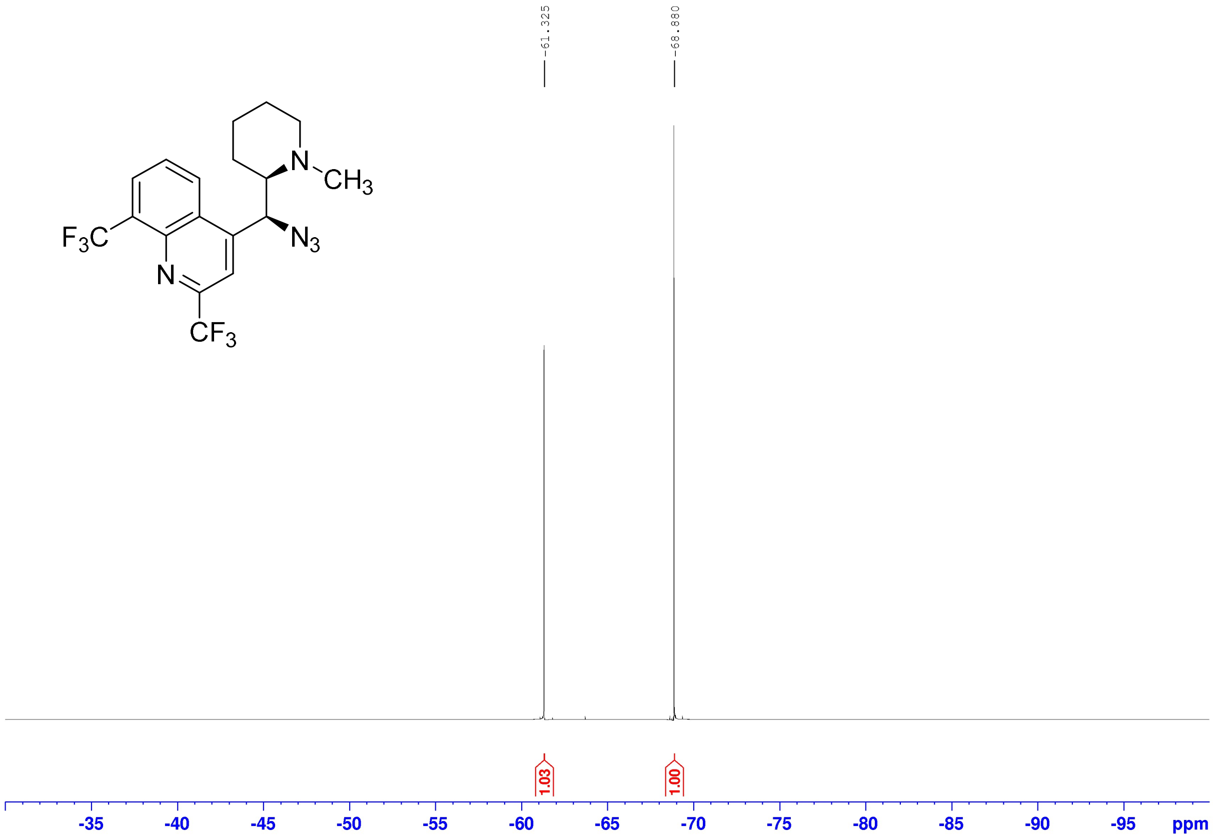

Figure S17. ^{19}F NMR (376 MHz) spectrum for 6**b** in $CDCl_3$+$PhCF_3$ ($\delta_F = -63.72$ ppm)

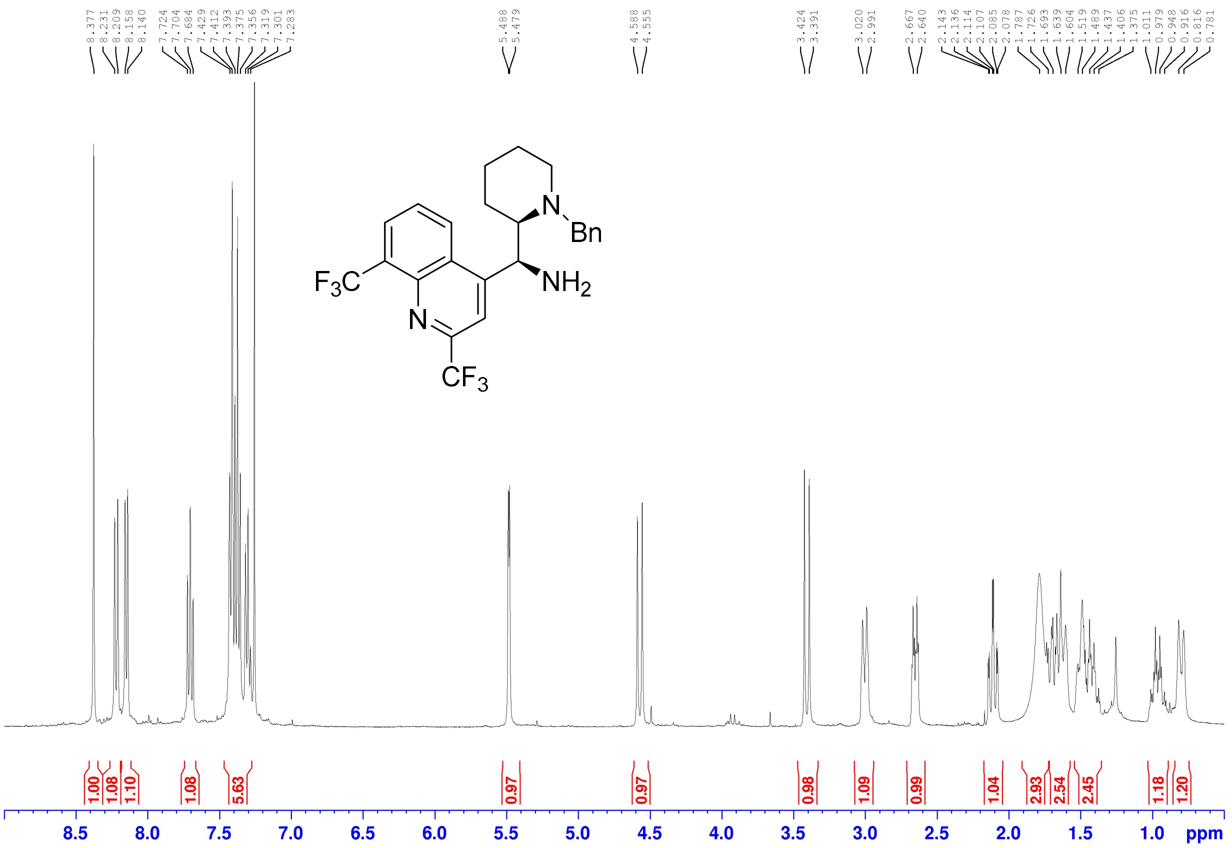

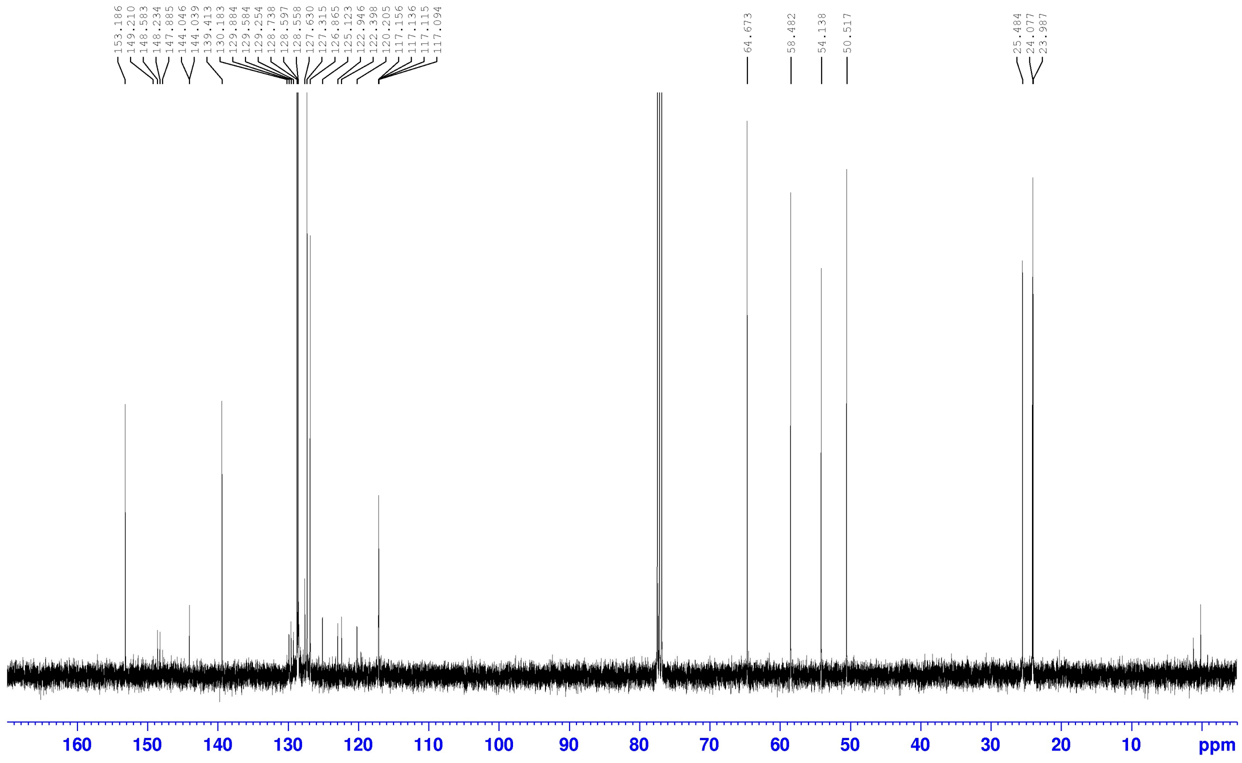

Figure S18. ^{1}H (400 MHz) and $^{13}C\{^{1}H\}$ NMR (100 MHz) spectra for **7a** in $CDCl_3$+TMS

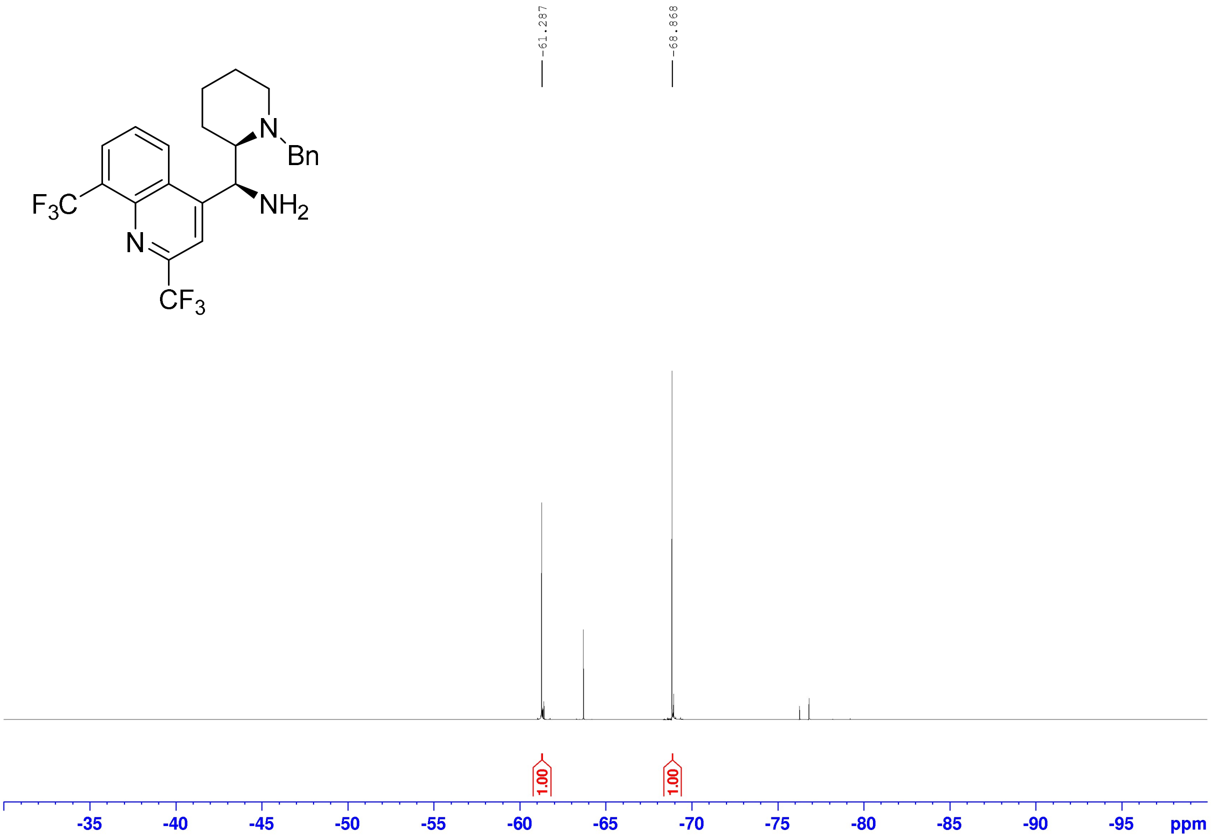

Figure S19. ^{19}F NMR (376 MHz) spectrum for **7a** in $CDCl_3$+$PhCF_3$ ($\delta_F = -63.72$ ppm)

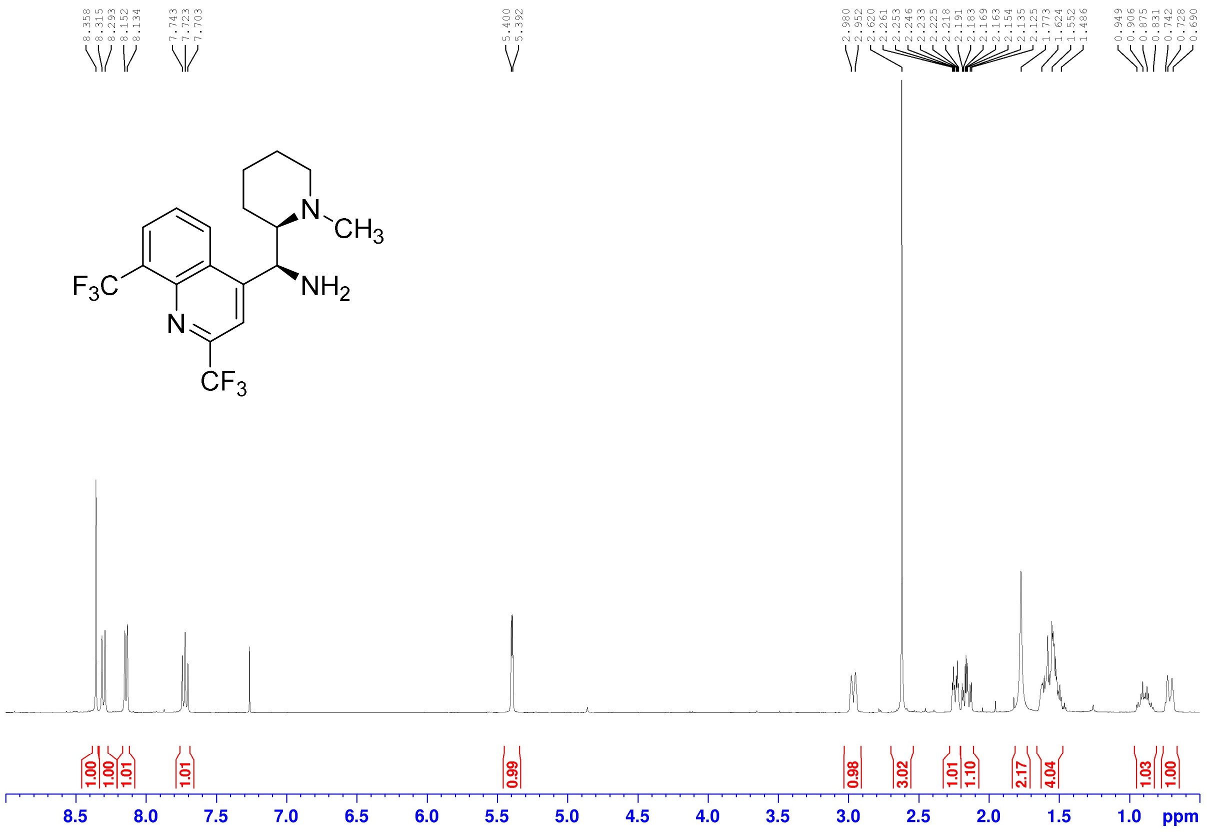

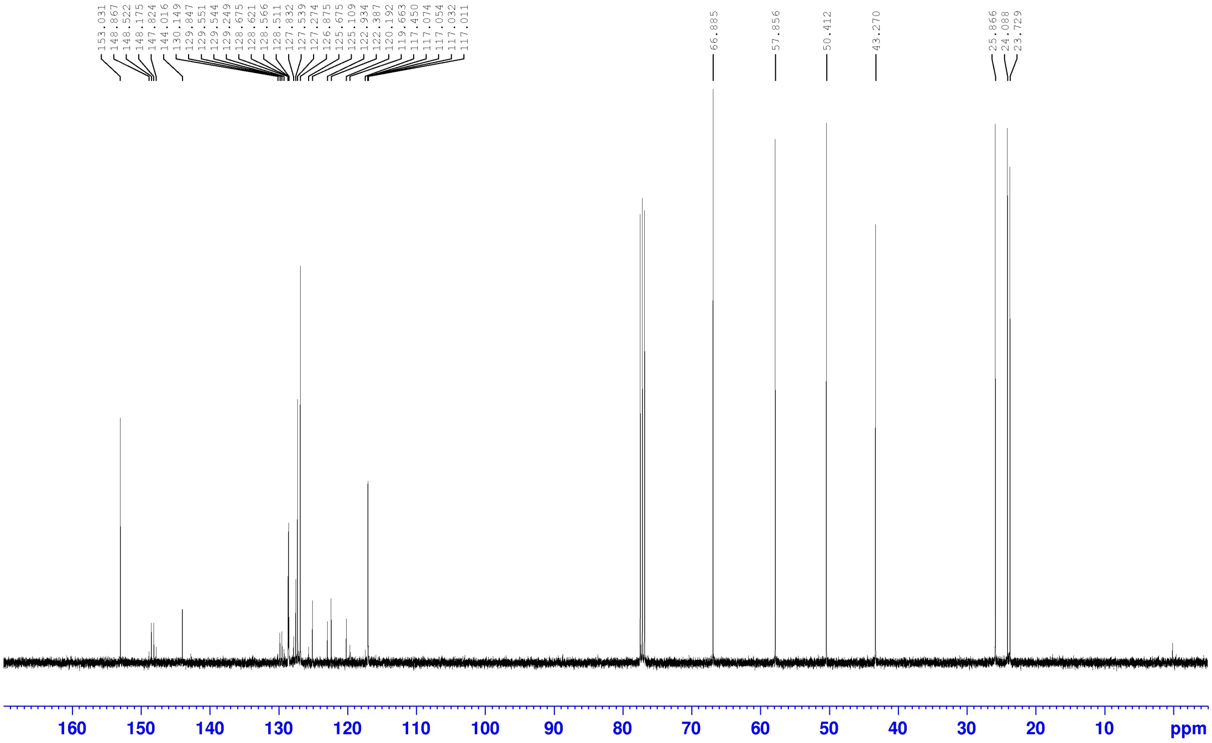

Figure S20. ^{1}H (400 MHz) and ^{13}C{^{1}H} NMR (100 MHz) spectra for **7b** in $CDCl_3$+TMS

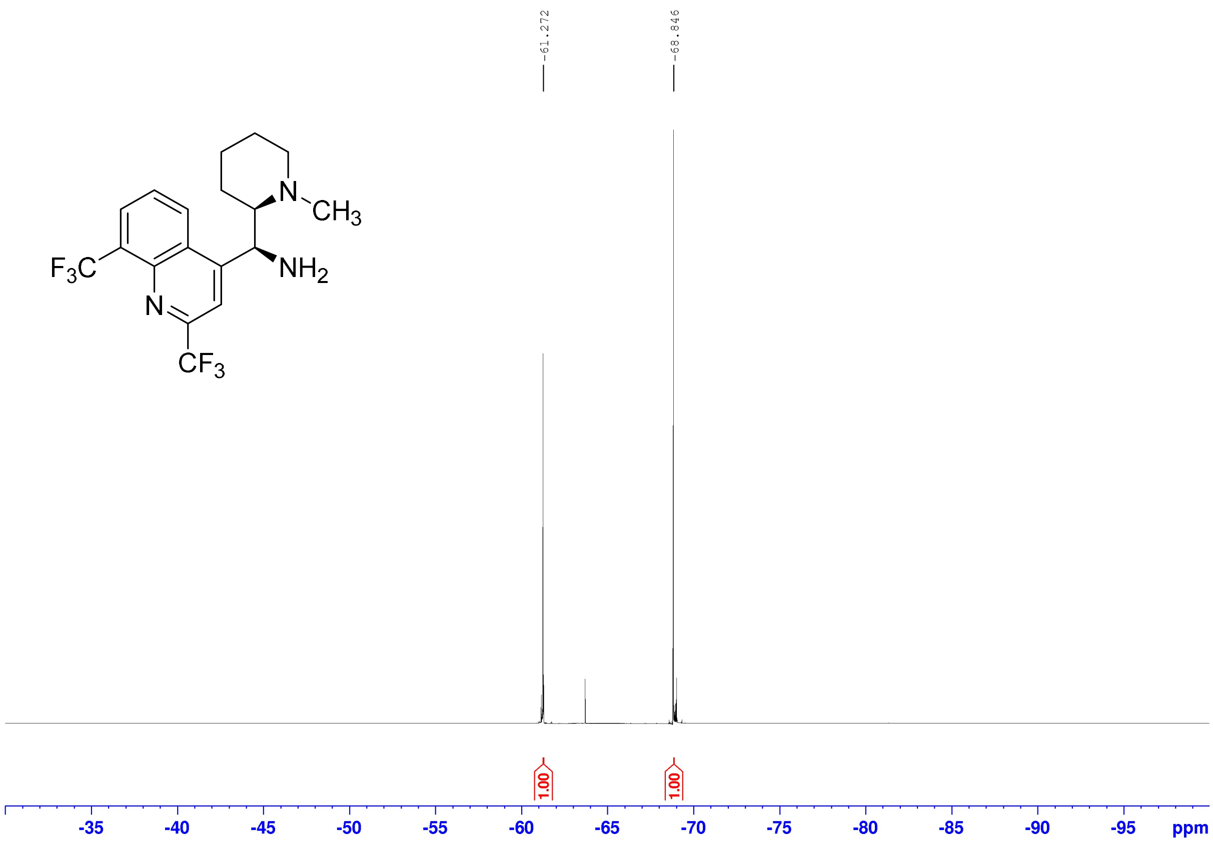

Figure S21. ^{19}F NMR (376 MHz) spectrum for **7b** in $CDCl_3$+$PhCF_3$ ($\delta_F = -63.72$ ppm)

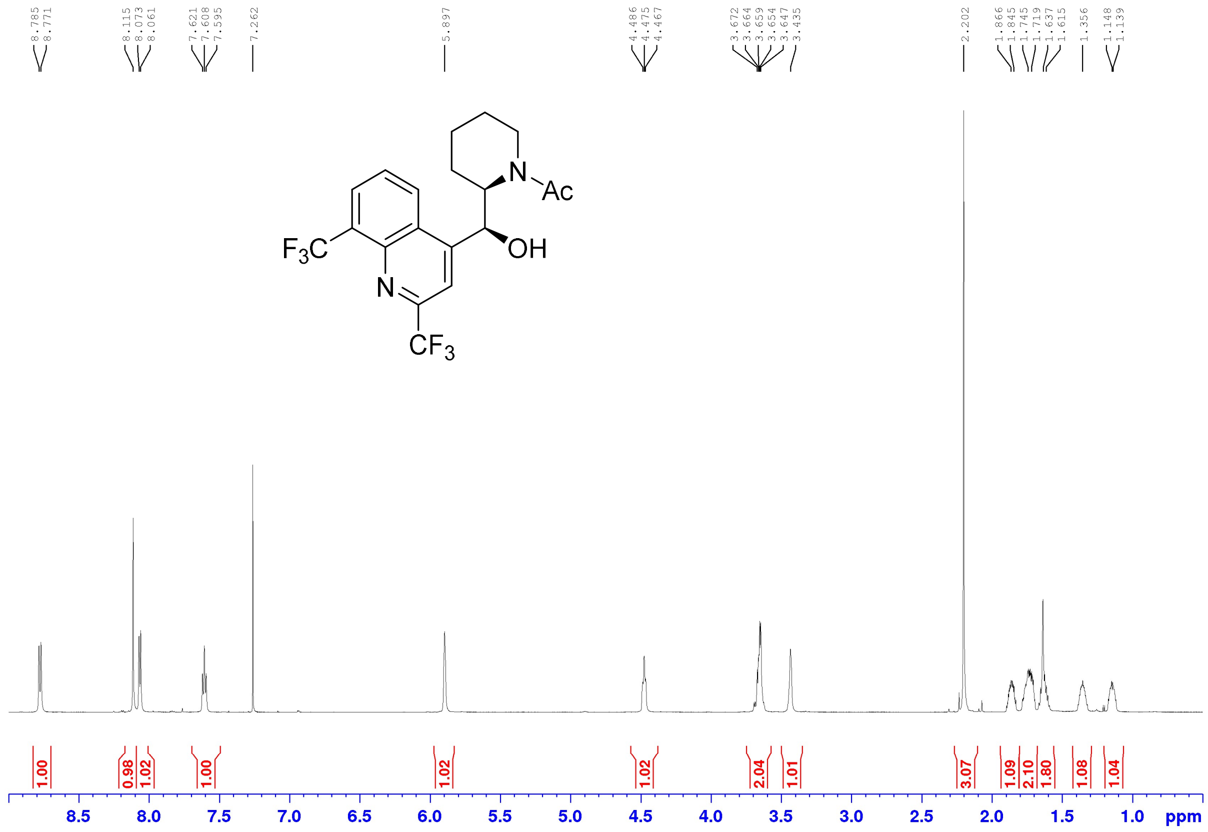

Figure S22. ^{1}H (600 MHz) spectrum for *erythro*-13-acetyl-mefloquine in $CDCl_3$+TMS

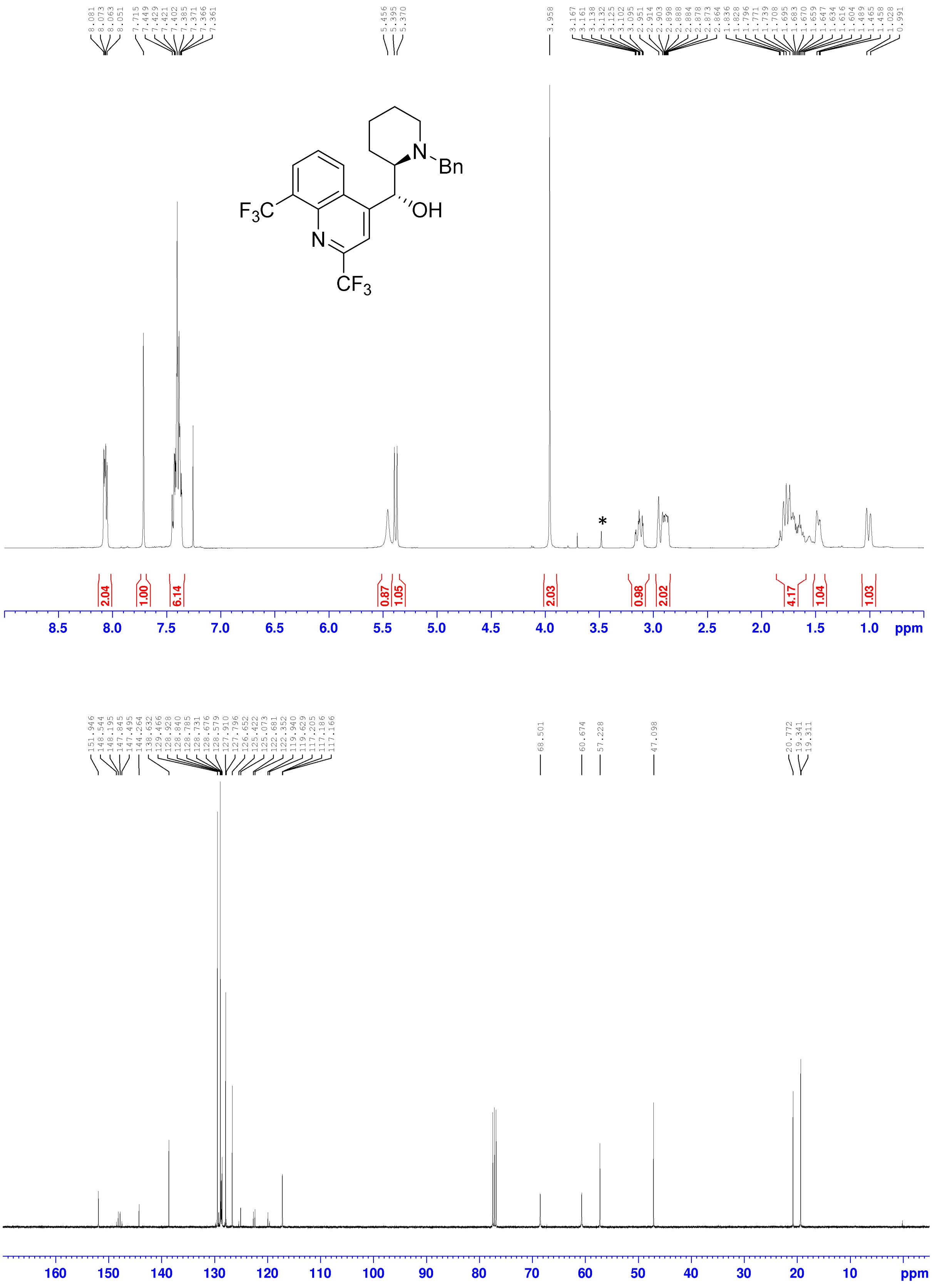

Figure S23. ^{1}H (400 MHz) and $^{13}C\{^{1}H\}$ NMR (100 MHz) spectra for **9** in $CDCl_3$+TMS. (* trace of MeOH identified)

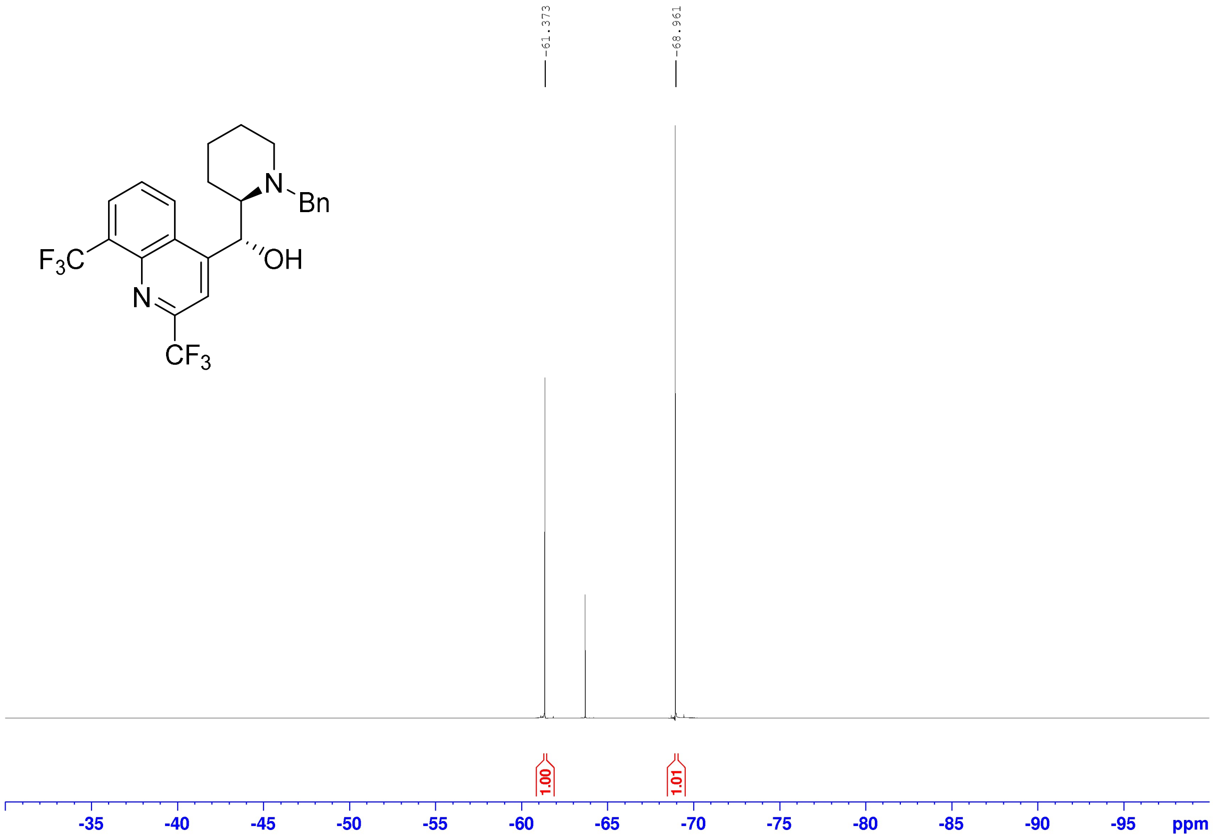

Figure S24. ^{19}F NMR (376 MHz) spectrum for **9** in $CDCl_3$+$PhCF_3$ (δ_F = −63.72 ppm)

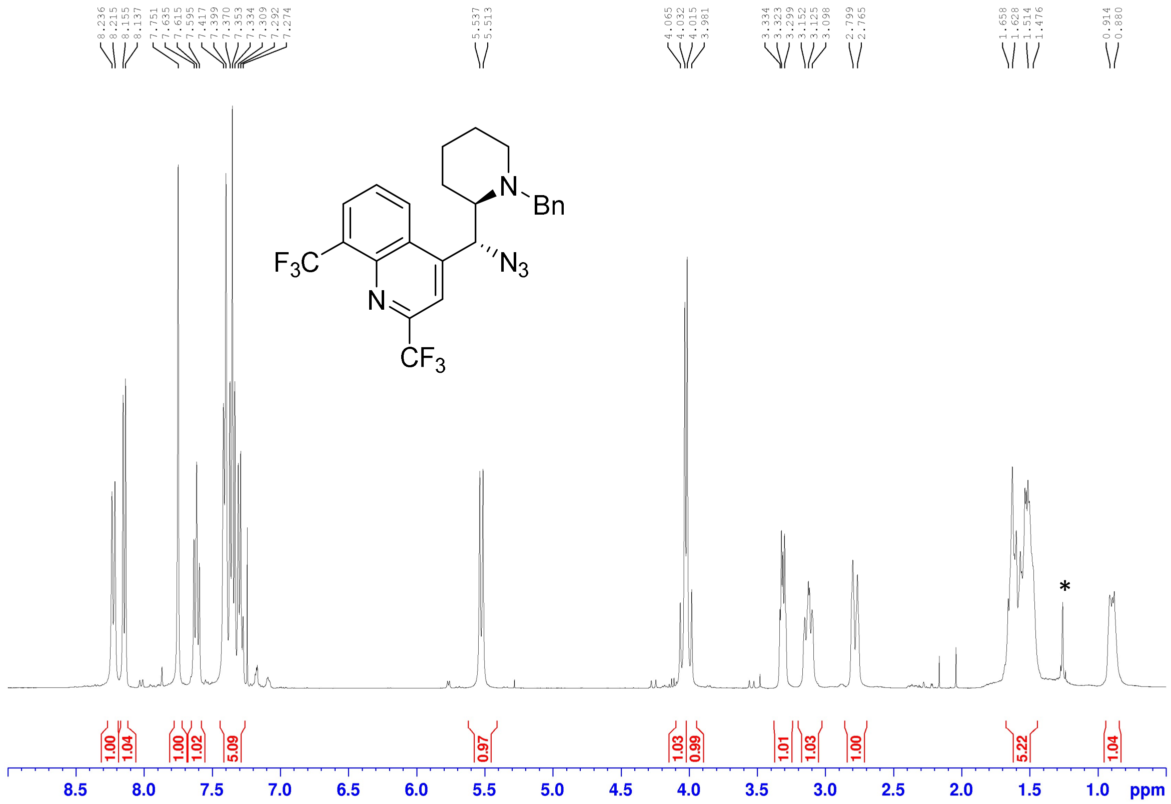

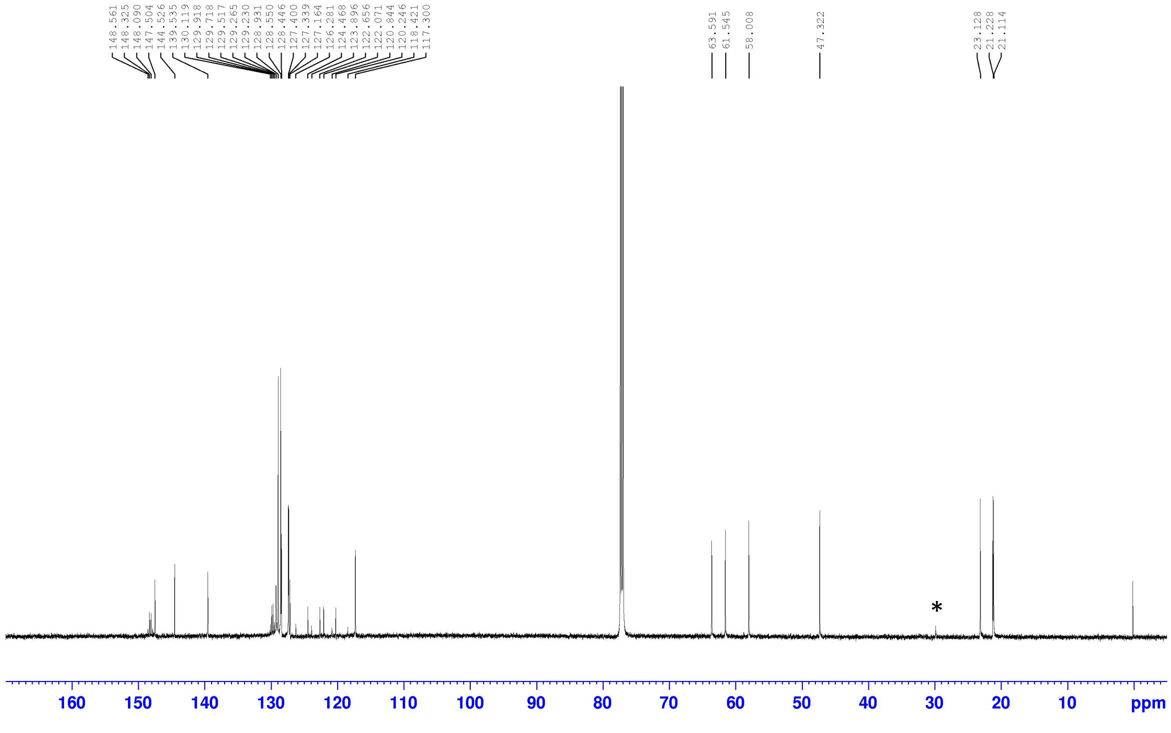

Figure S25. ^{1}H (400 MHz) and $^{13}C\{^{1}H\}$ NMR (151 MHz) spectra for **10** in $CDCl_3$+TMS (*trace of high boiling hydrocarbons identified)

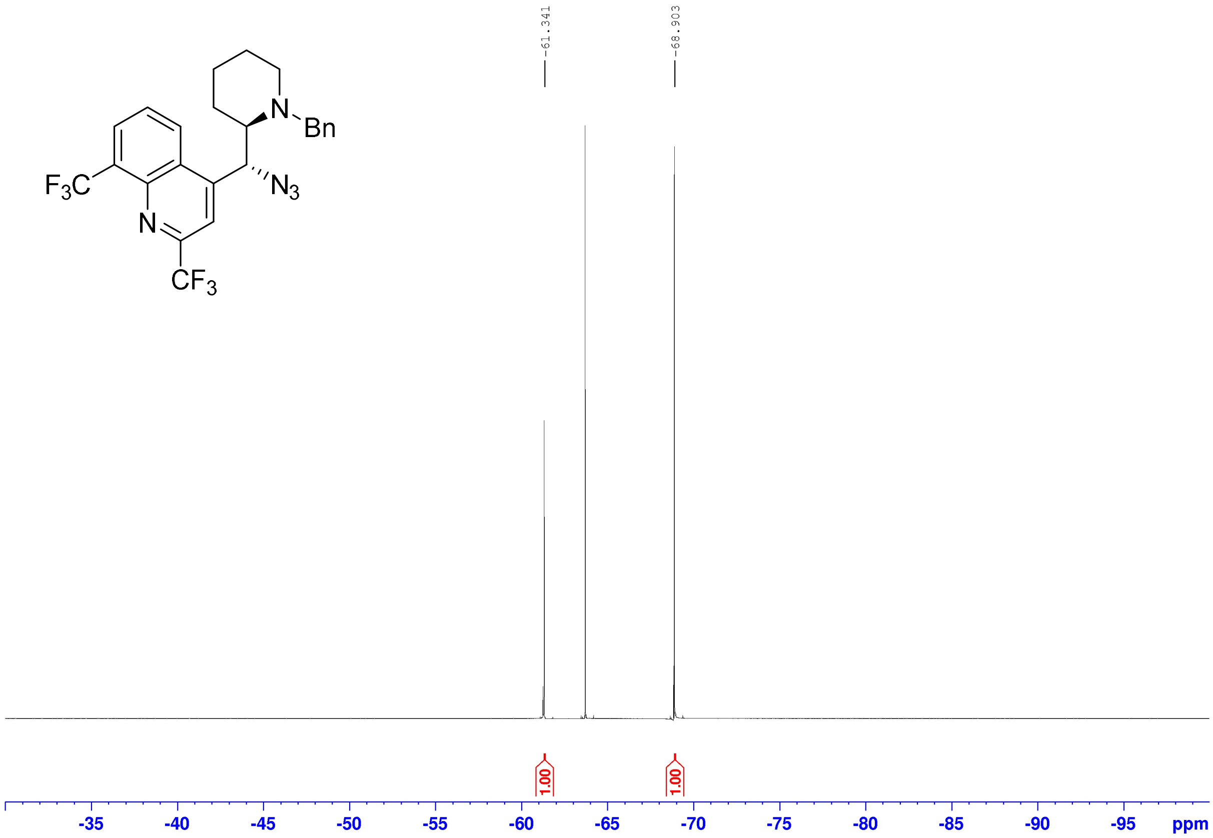

Figure S26. ^{19}F NMR (376 MHz) spectrum for **10** in $CDCl_3$+$PhCF_3$ (δ_F = −63.72 ppm)

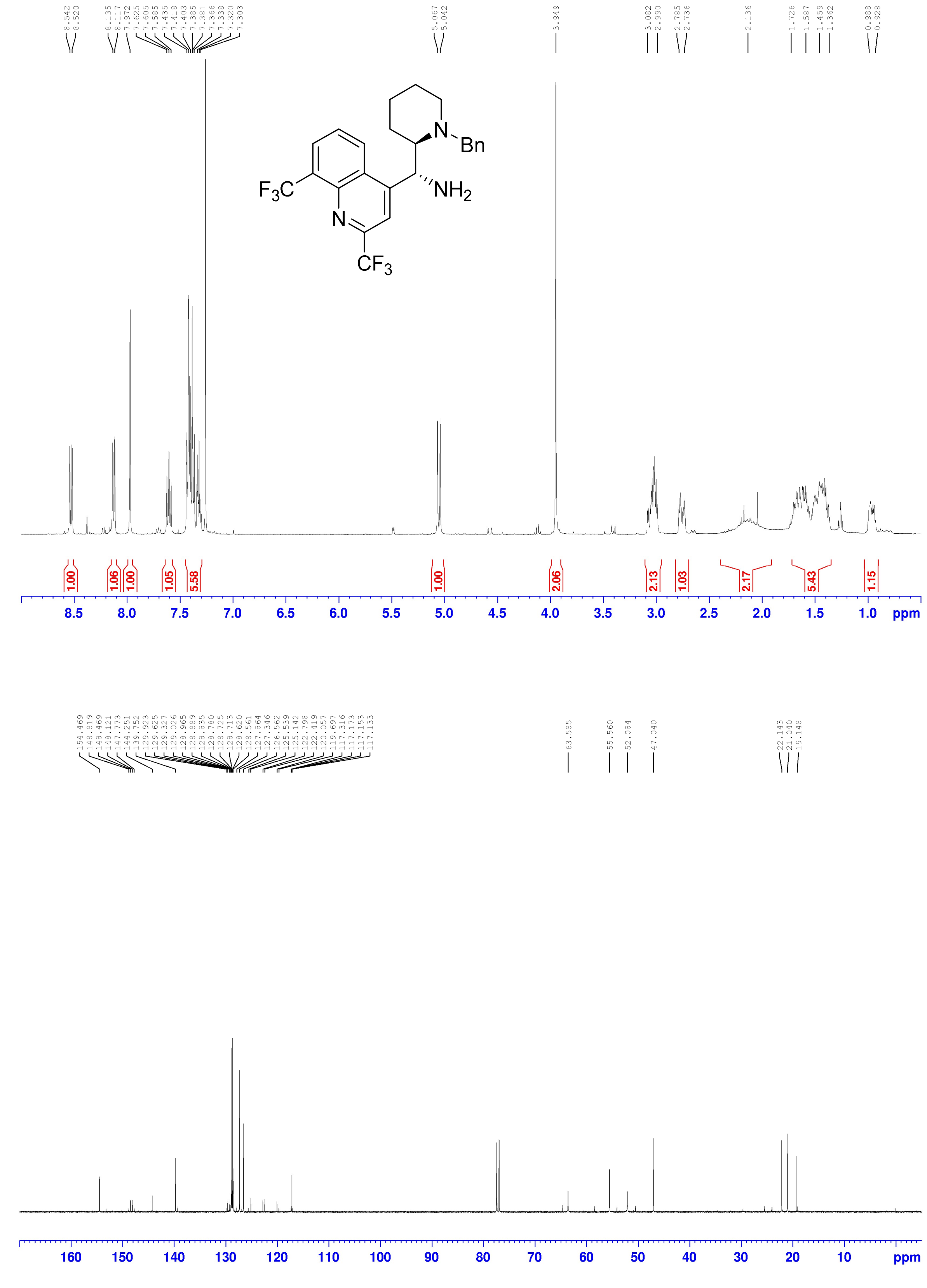

Figure S27. ^{1}H (400 MHz) and ^{13}C{^{1}H} NMR (100 MHz) spectra for **11** in $CDCl_3$+TMS

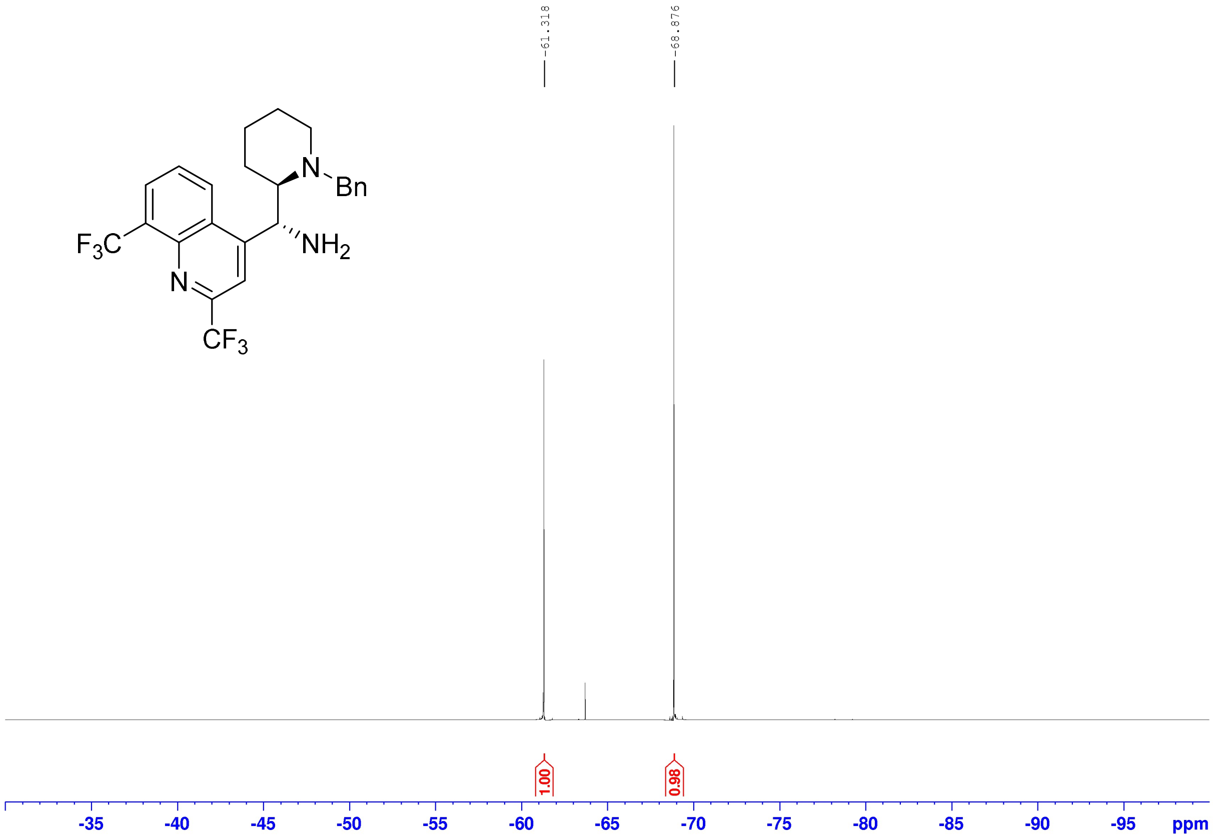

Figure S28. ^{19}F NMR (376 MHz) spectrum for **11** in $CDCl_3$+$PhCF_3$ (δ_F = −63.72 ppm)

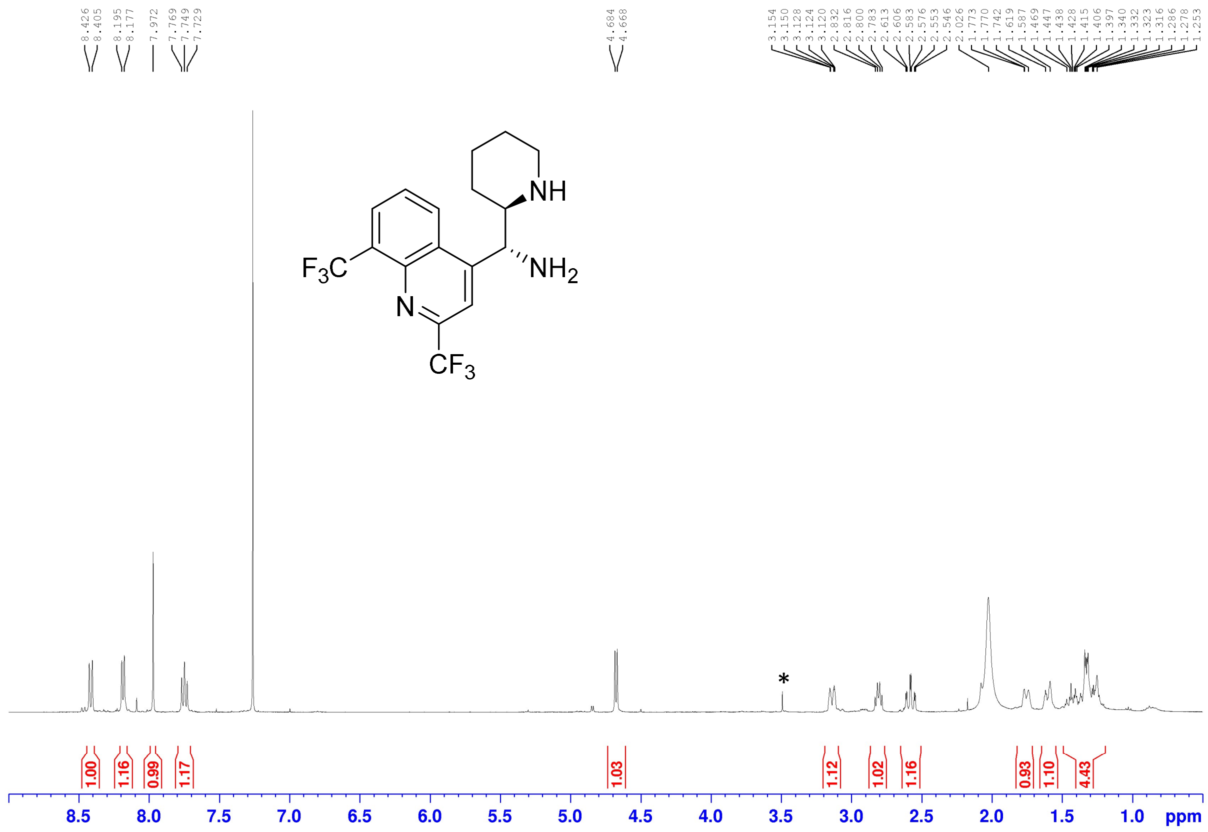

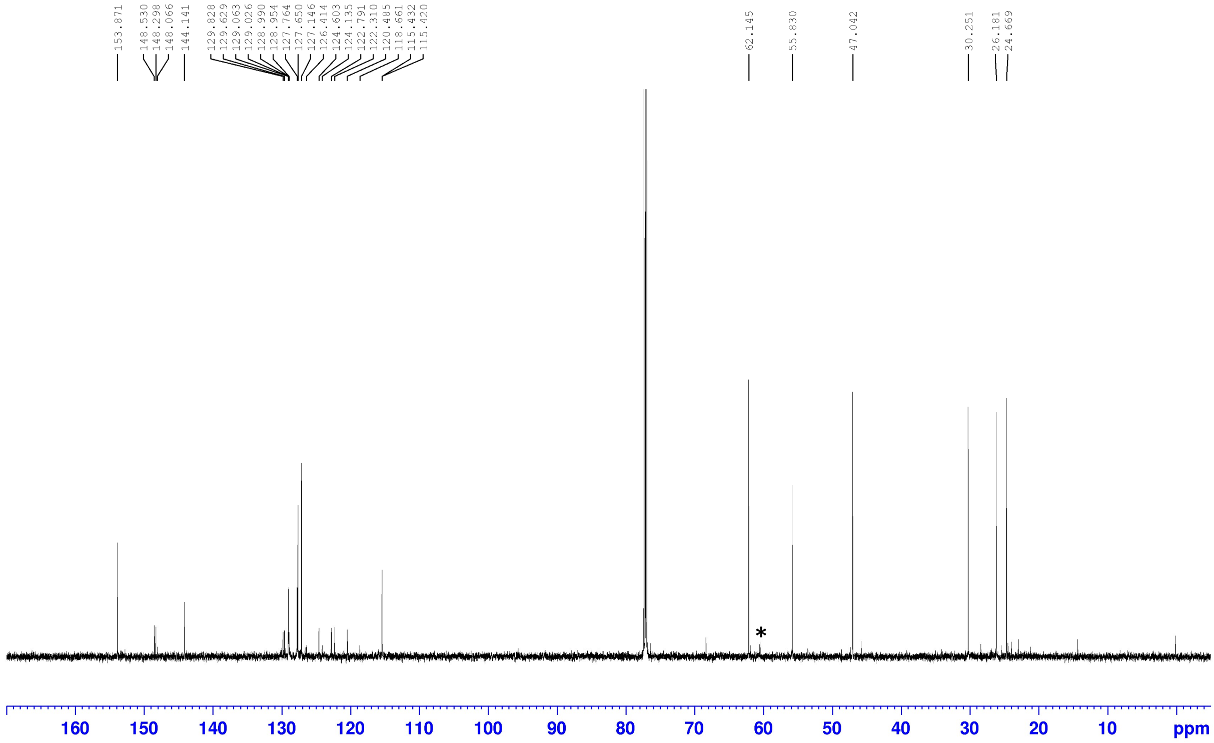

Figure S29. ^{1}H (400 MHz) and ^{13}C{^{1}H} NMR (151 MHz) spectra for **12** in $CDCl_3$+TMS. (* trace of MeOH identified)

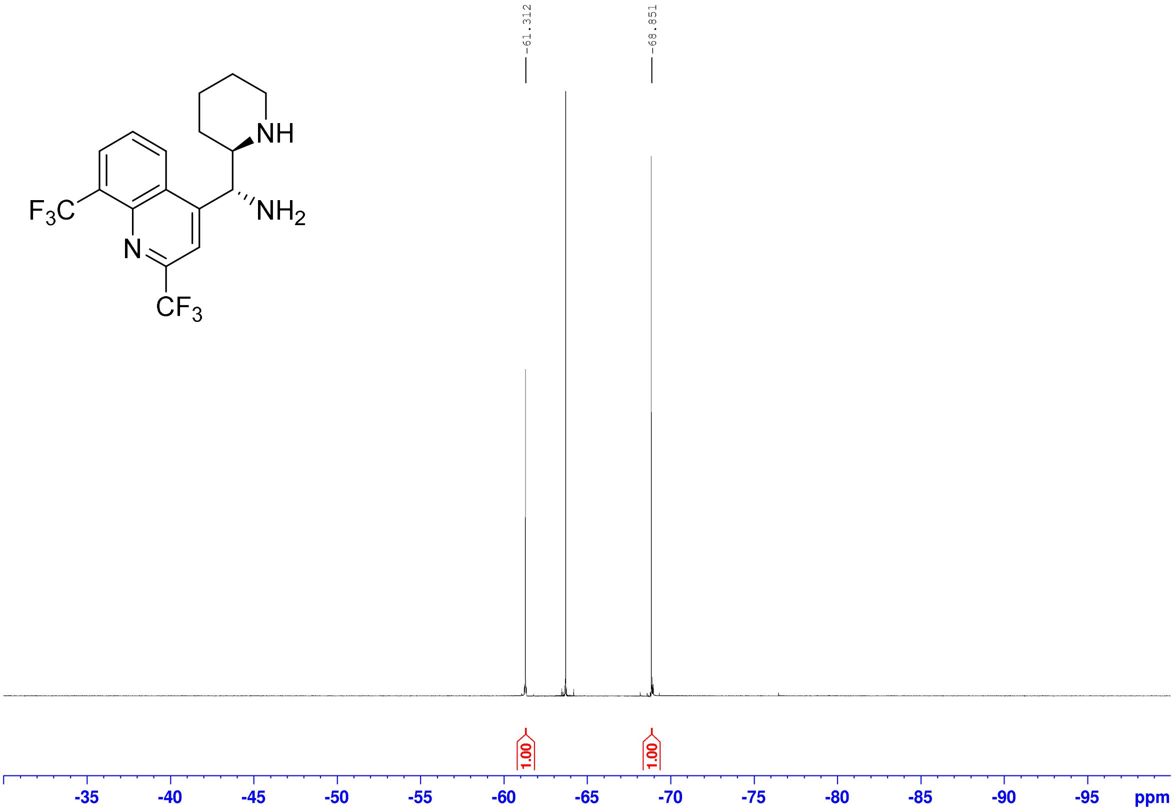

Figure S30. ^{19}F NMR (376 MHz) spectrum for **12** in $CDCl_3$+$PhCF_3$ ($\delta_F = -63.72$ ppm)

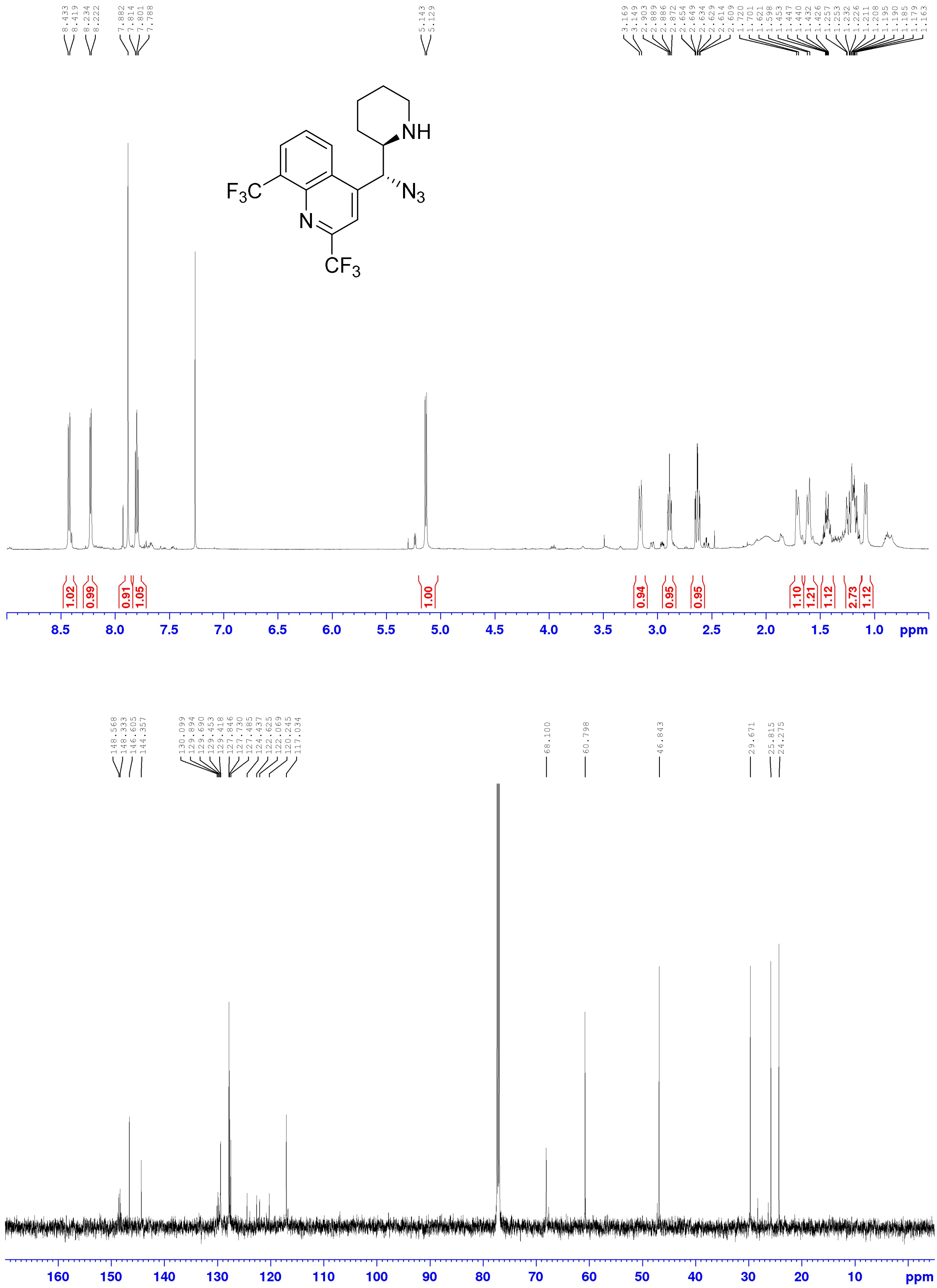

Figure S31. ^{1}H (600 MHz) and ^{13}C{^{1}H} NMR (151 MHz) spectra for **13**, diasteromeric ratio 95 : 5, in $CDCl_3$+TMS. For spectra of diasteromer, see Figures S4-S5.

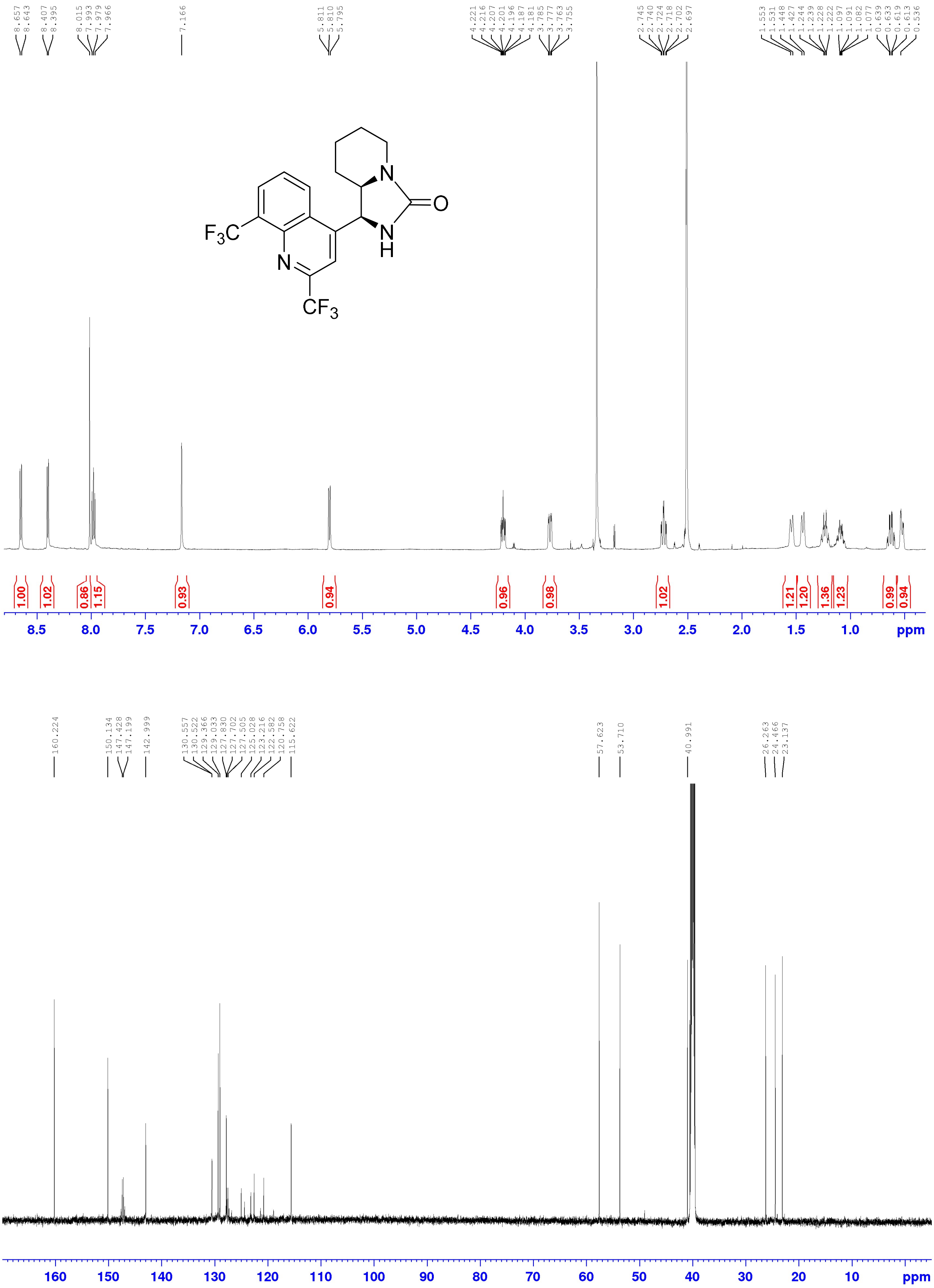

Figure S32. ^{1}H (600 MHz) and ^{13}C{^{1}H} NMR (151 MHz) spectra for **14** in DMSO-d_6+TMS

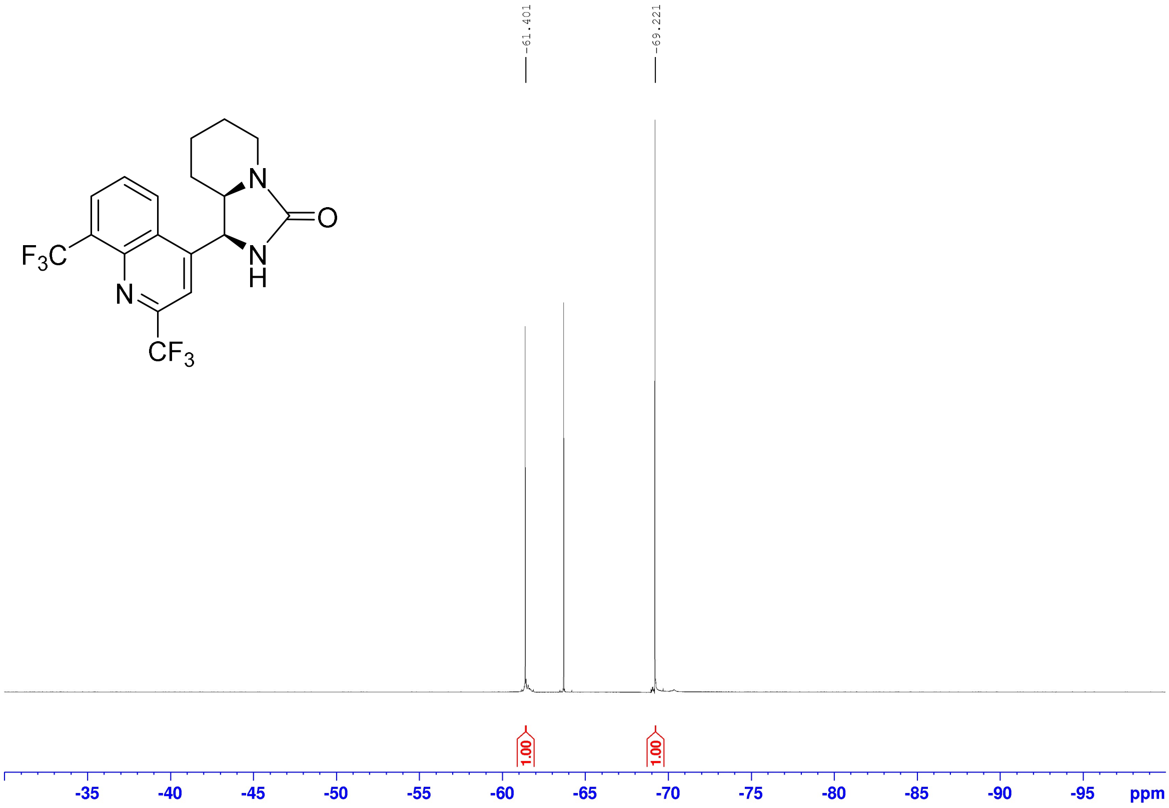

Figure S33. ^{19}F NMR (376 MHz) spectrum for **14** in $CDCl_3$+$PhCF_3$ (δ_F = −63.72 ppm)

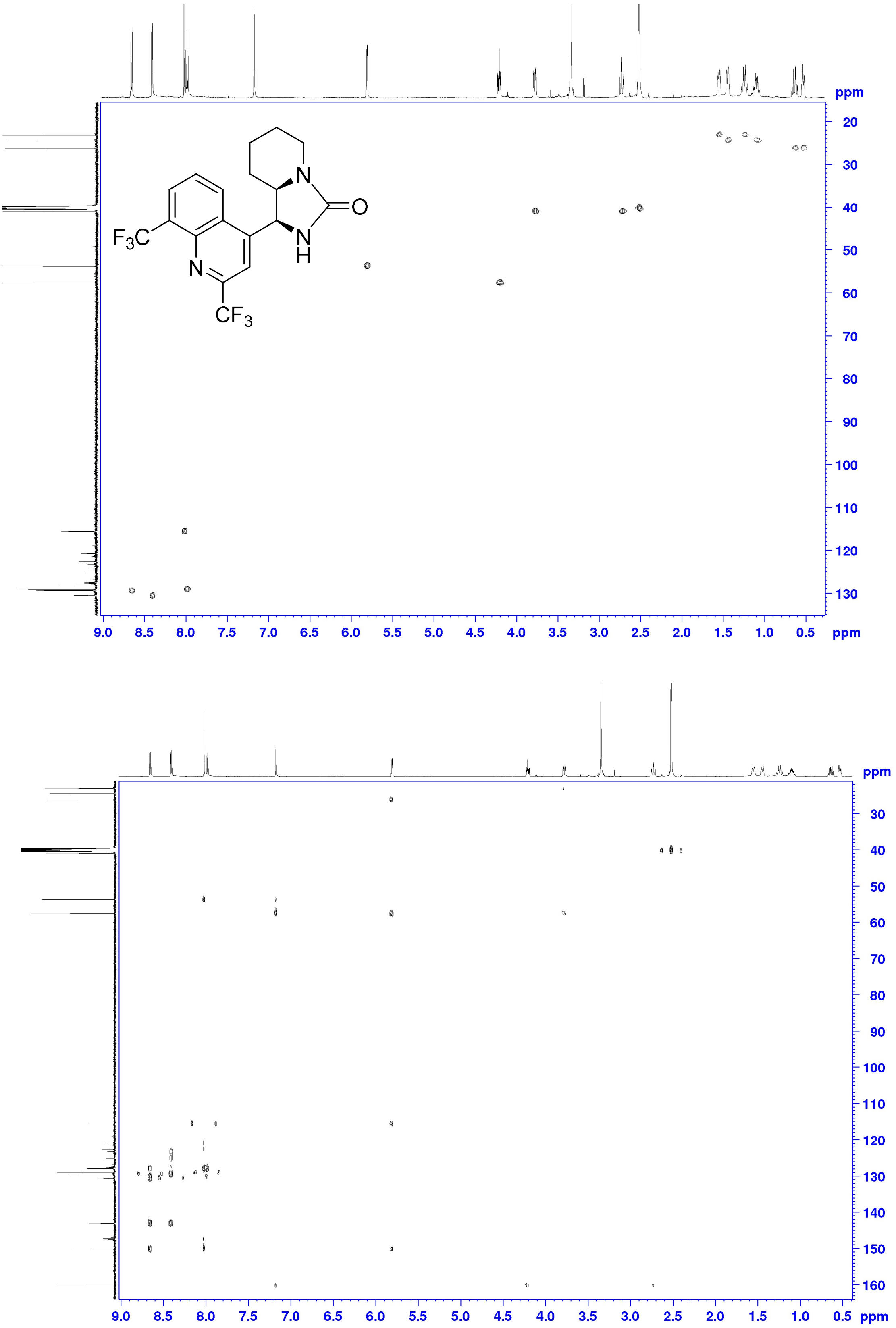

Figure S34. $^{1}H,^{13}C$ HSQC (top) and HMBC (bottom) experiments for **14** in DMSO-d_6. For spectral assignment, see Figure S2.

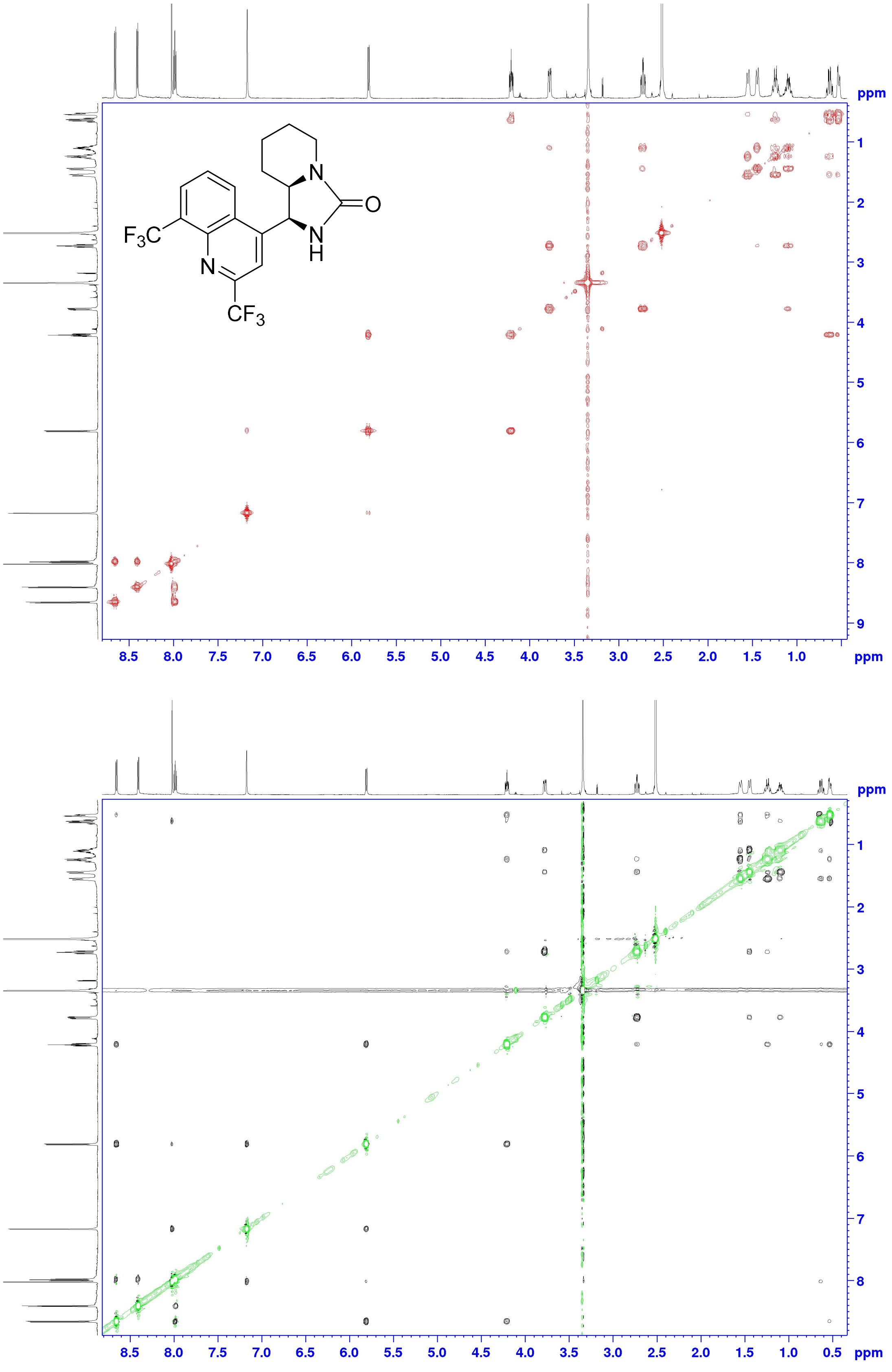

Figure S35. ^{1}H,^{1}H COSY (top) and NOESY (bottom, phases of opposite signs are drawn in black and green) experiments for **14** in DMSO-*d*$_6$. For spectral assignment, see Figure S2.

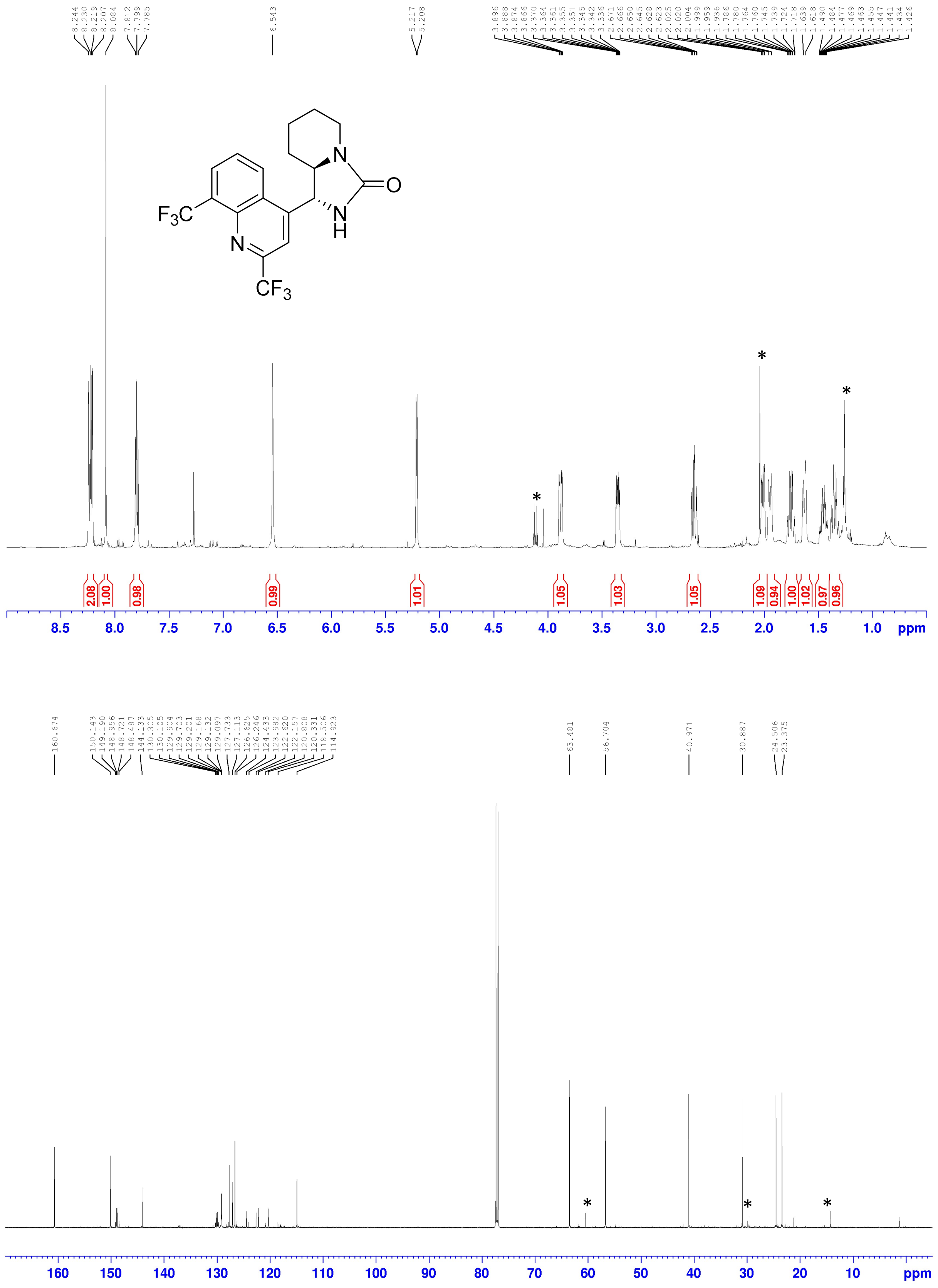

Figure S36. ^{1}H (600 MHz) and ^{13}C{^{1}H} NMR (151 MHz) spectra for **15** in $CDCl_3$+TMS. (*trace of EtOAc was identified)

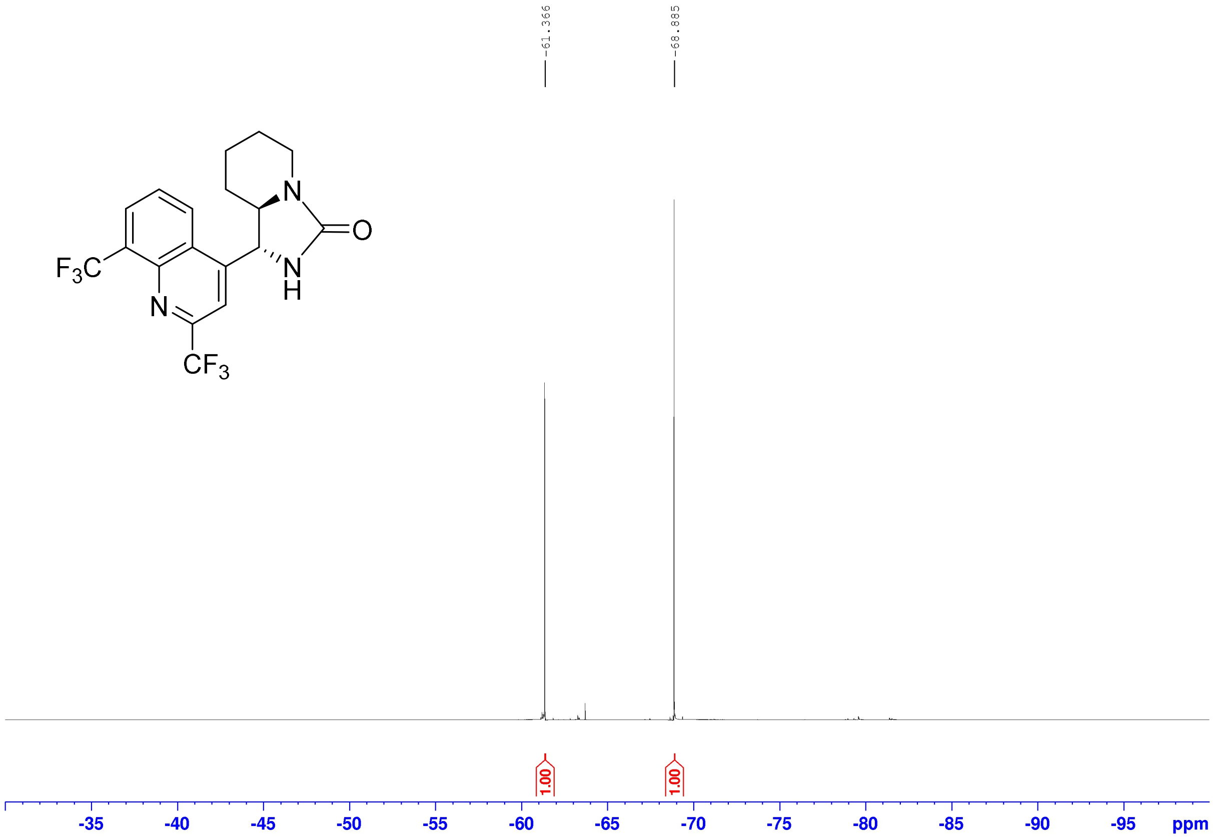

Figure S37. ^{19}F NMR (376 MHz) spectrum for **15** in $CDCl_3$+$PhCF_3$ (δ_F = −63.72 ppm)

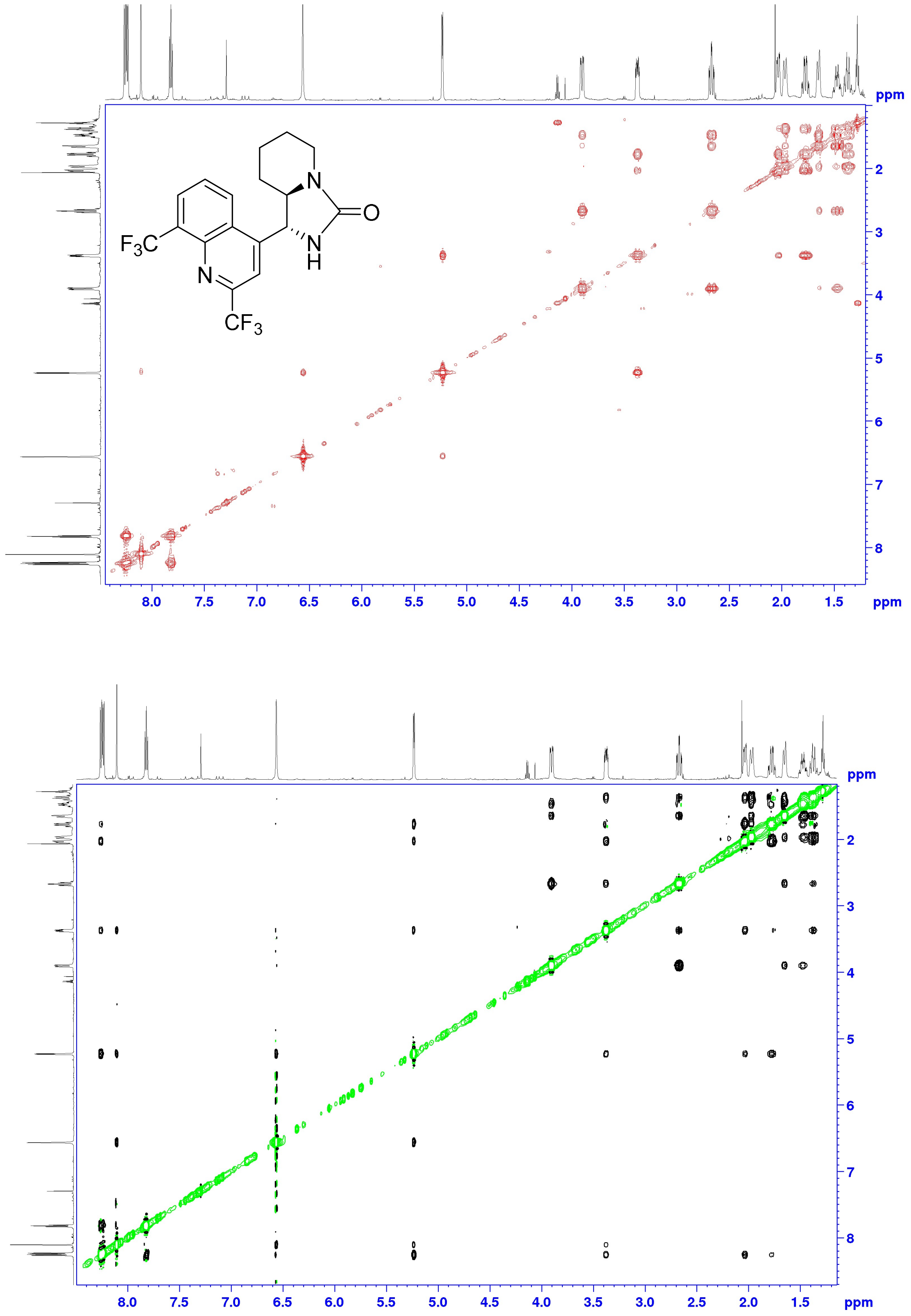

Figure S38. ^{1}H,^{1}H COSY (top) and NOESY (bottom, phases of opposite signs are drawn in black and green) experiments for **15** in $CDCl_3$. For spectral assignment, see Figure S2.

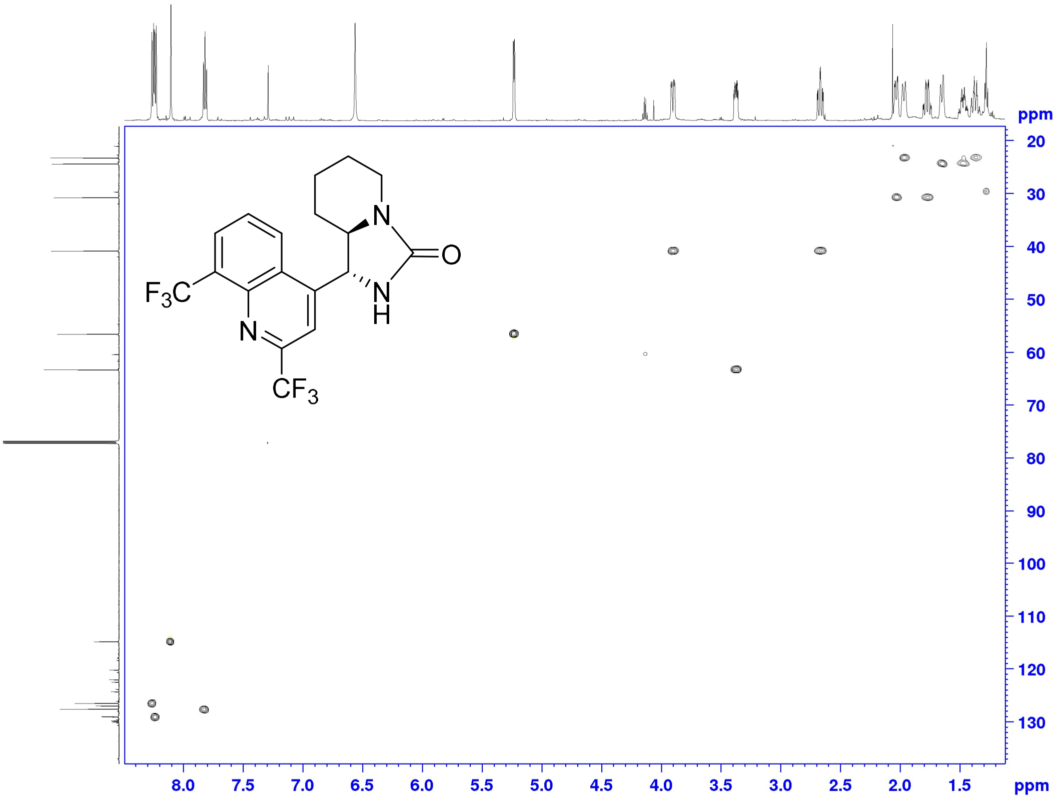

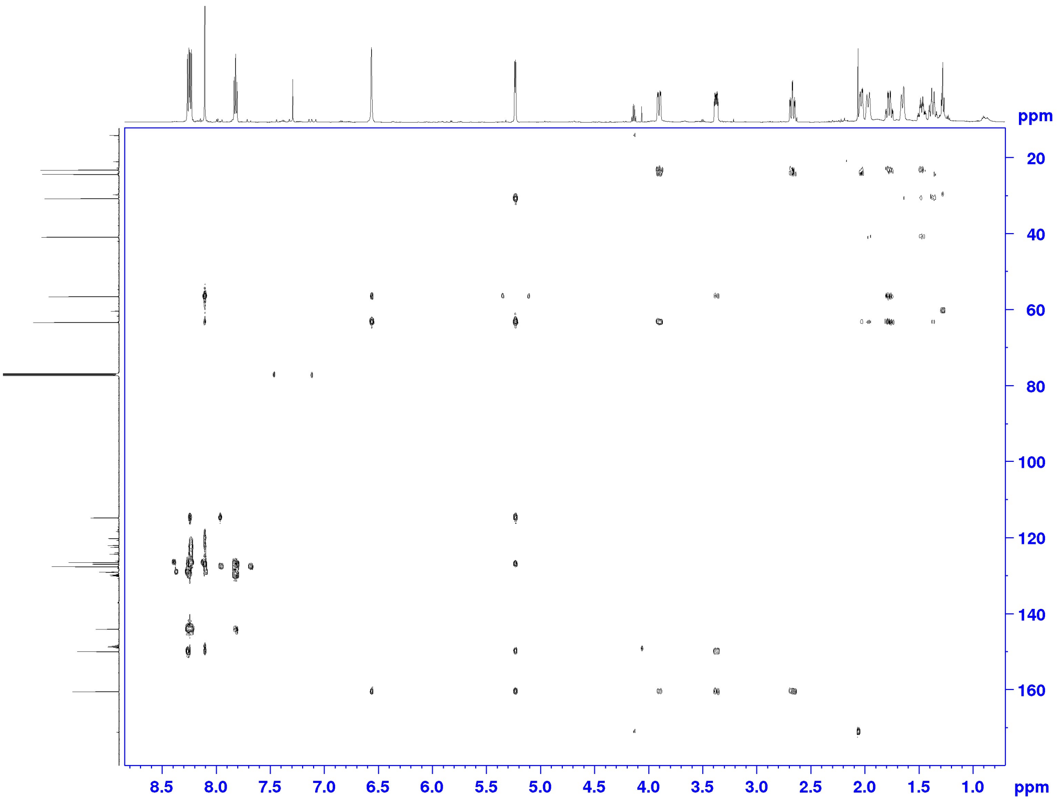

Figure S39. $^{1}H,^{13}C$ HSQC (top) and HMBC (bottom) experiments for **15** in $CDCl_3$. For spectral assignment, see Figure S2.

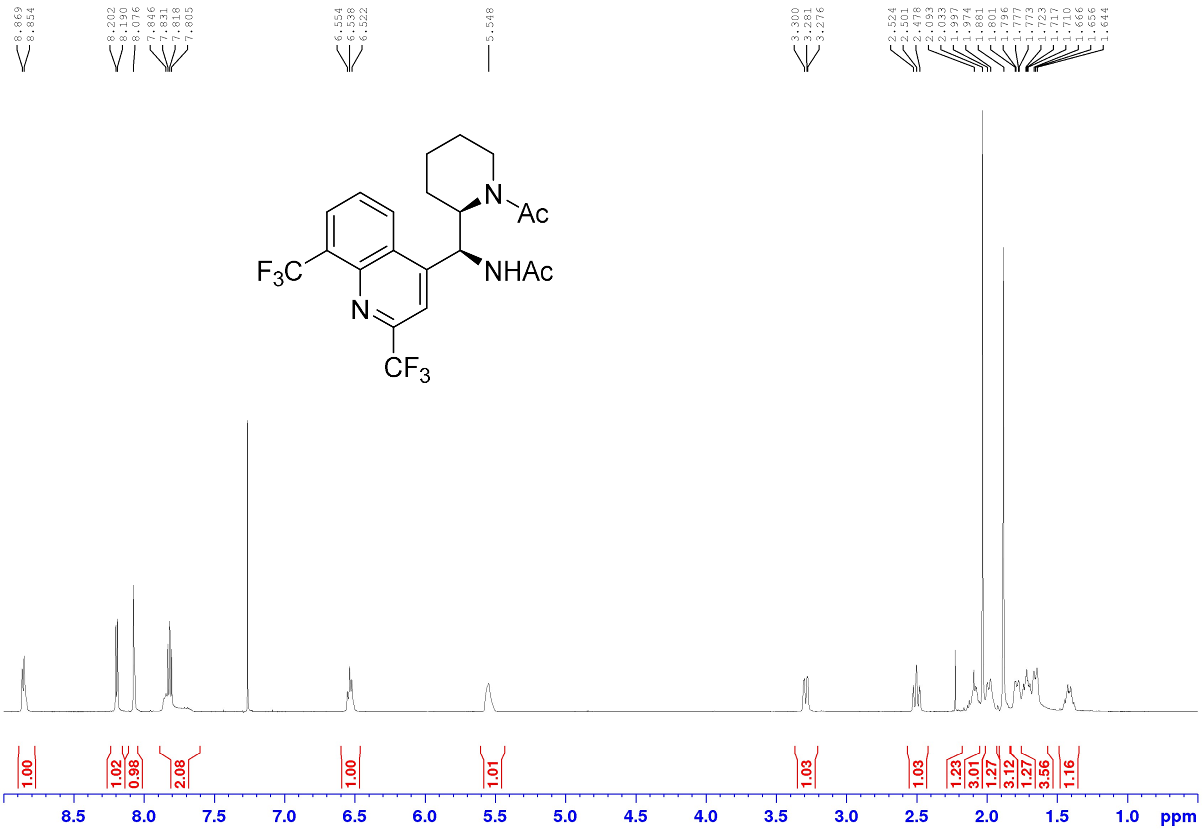

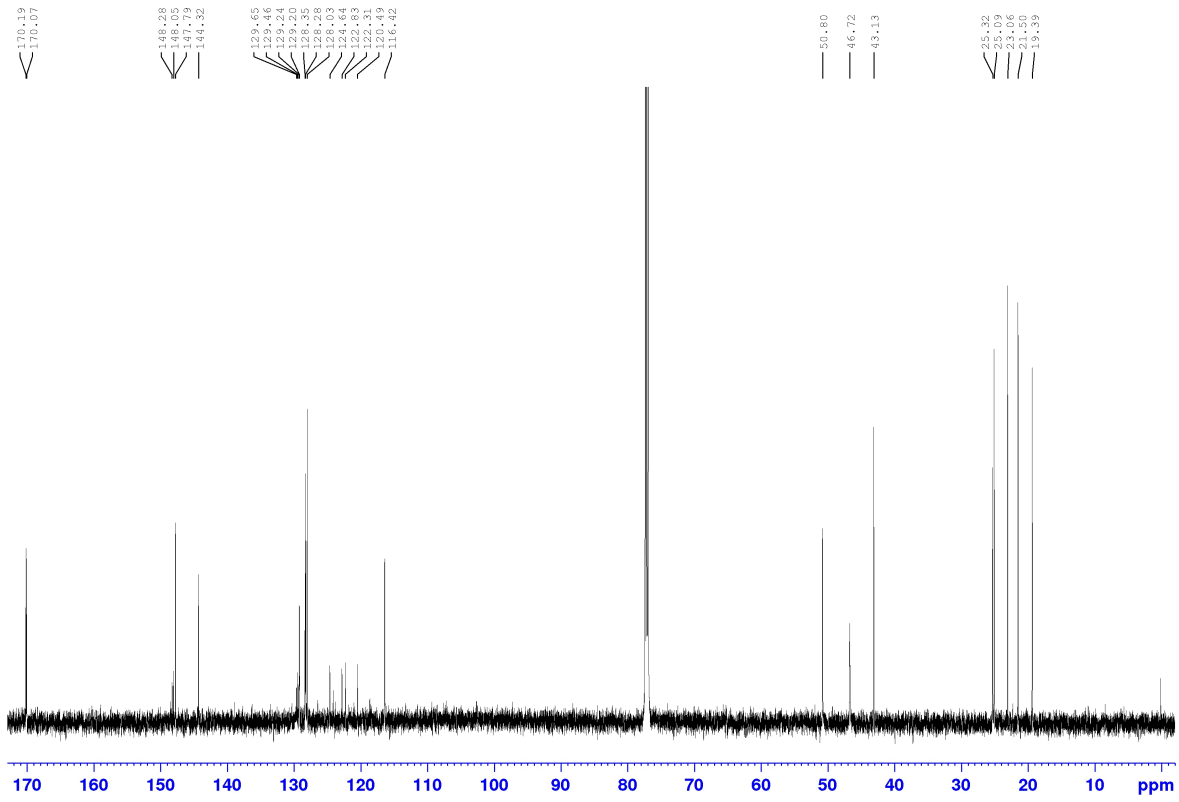

Figure S40. ^{1}H (600 MHz) and ^{13}C{^{1}H} NMR (151 MHz) spectra for **16** in $CDCl_3$+TMS

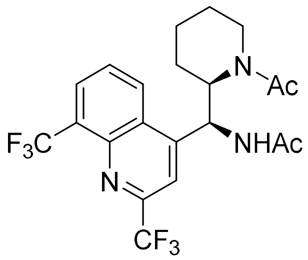

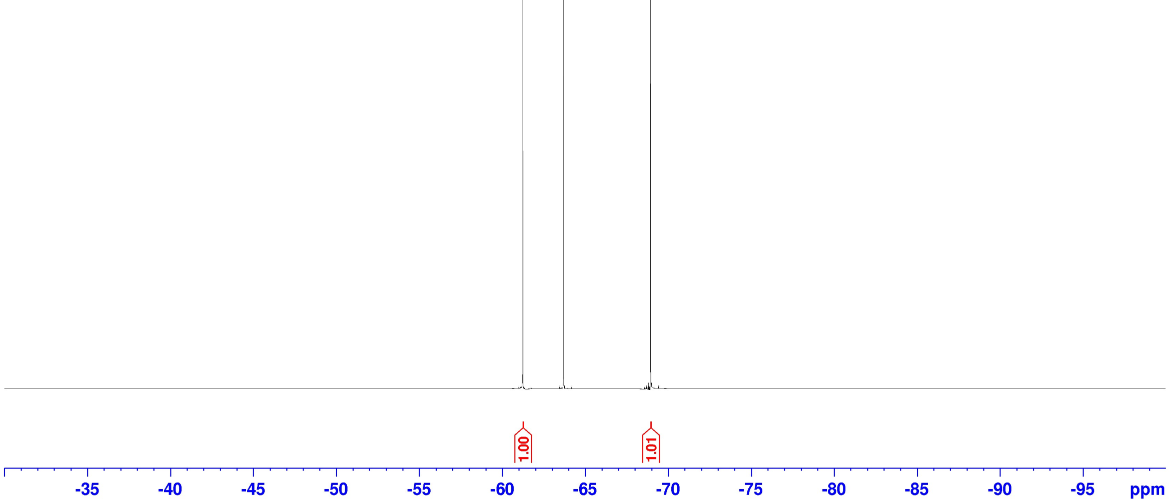

Figure S41. ^{19}F NMR (376 MHz) spectrum for **16** in $CDCl_3$+$PhCF_3$ ($\delta_F = -63.72$ ppm)

Spectral data for 11-chloro-13-benzyl-mefloquine hydrochloride

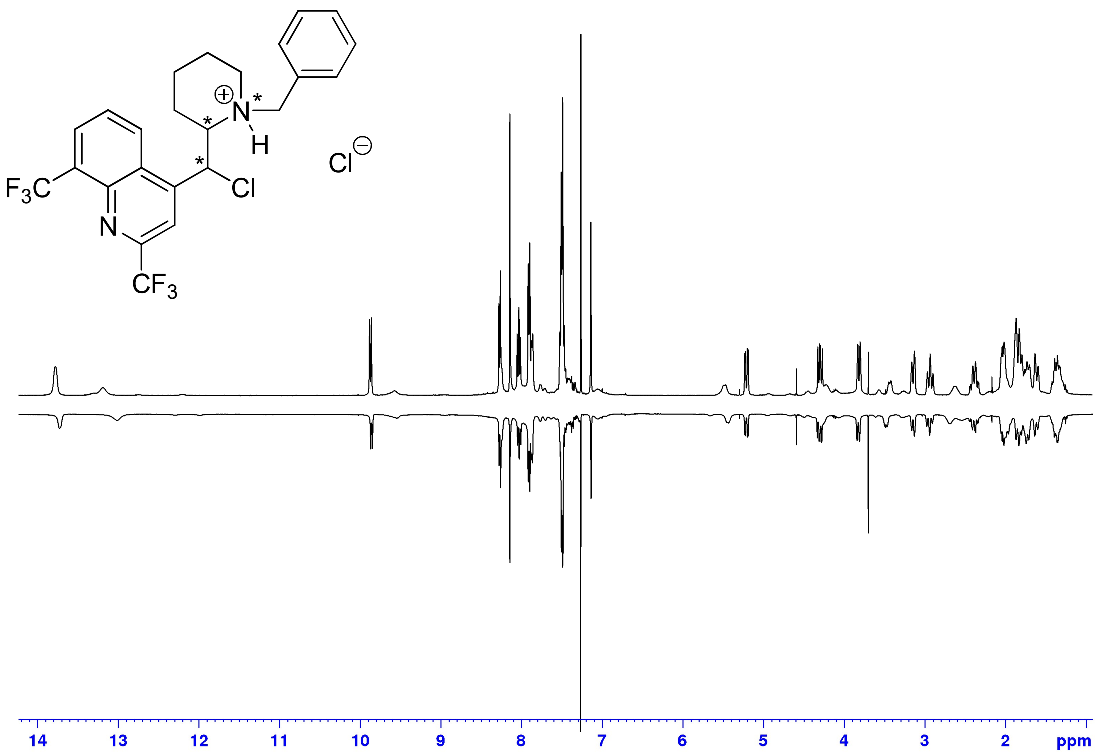

Figure S42. ^{1}H NMR (400 MHz, $CDCl_3$) spectra for 11-chloroderivatives obtained after thionyl chloride treatment of 13-benzyl-mefloquine of *erythro* configuration (**5a**, positive phase) and *threo* configuration (**9**, negative phase).

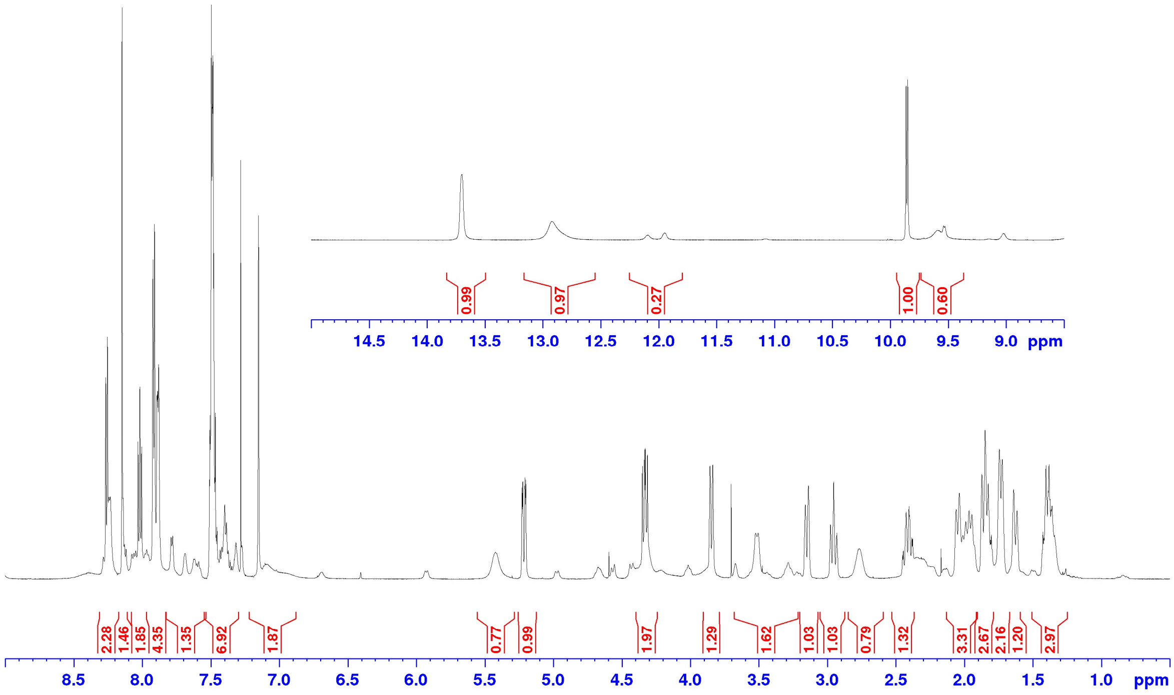

Figure S43. ^{1}H NMR (600 MHz, $CDCl_3$) spectra for 11-chloroderivatives obtained after thionyl chloride treatment of 13-benzyl-mefloquine. See preceding Figure S42 for comparison of samples obtained from different starting materials.

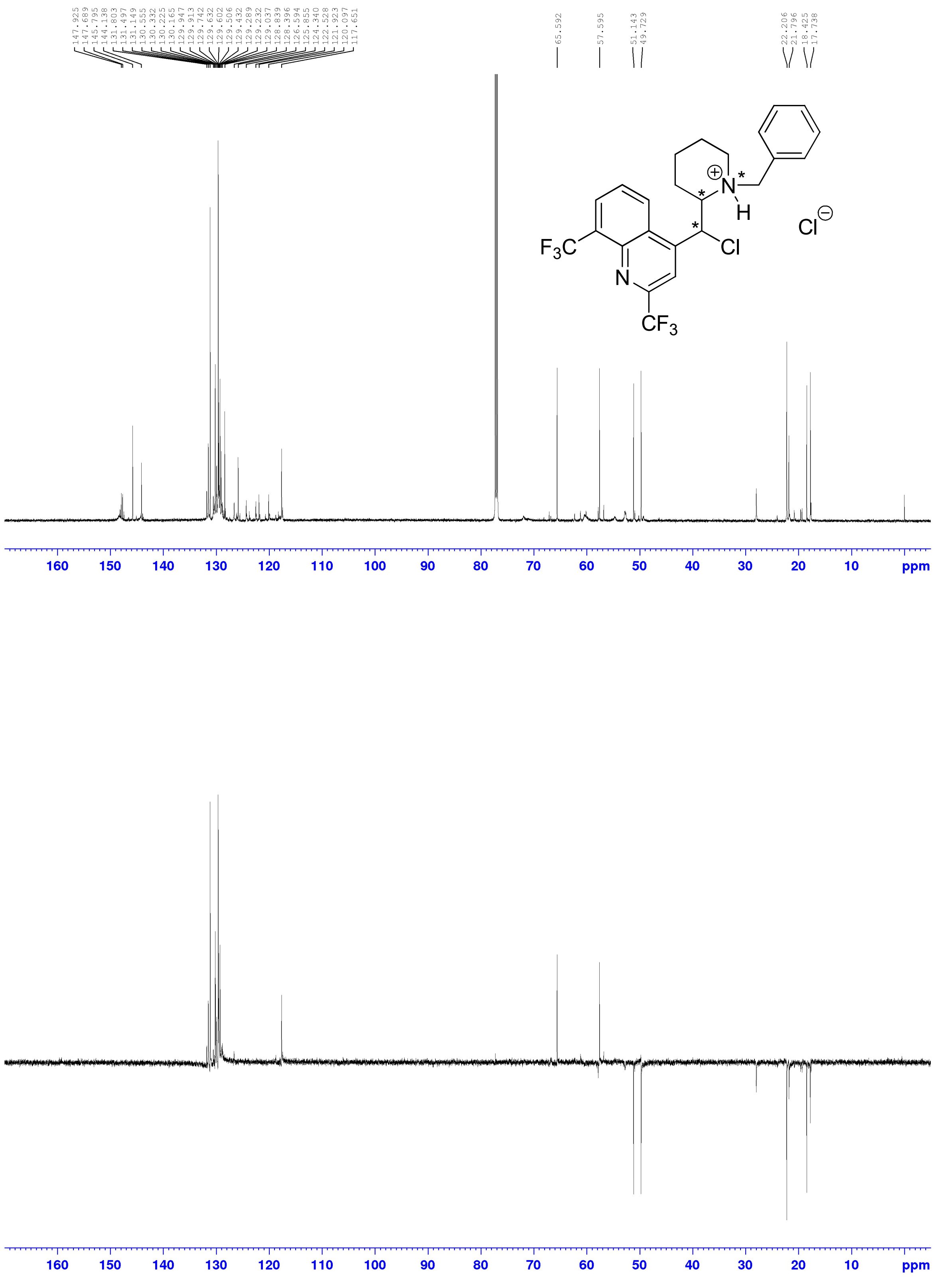

Figure S44. $^{13}C\{^{1}H\}$ and ^{13}C DEPT-135° NMR experiments (151 MHz, $CDCl_3$) for likely 11-chloroderivatives obtained after thionyl chloride treatment of 13-benzyl-mefloquine

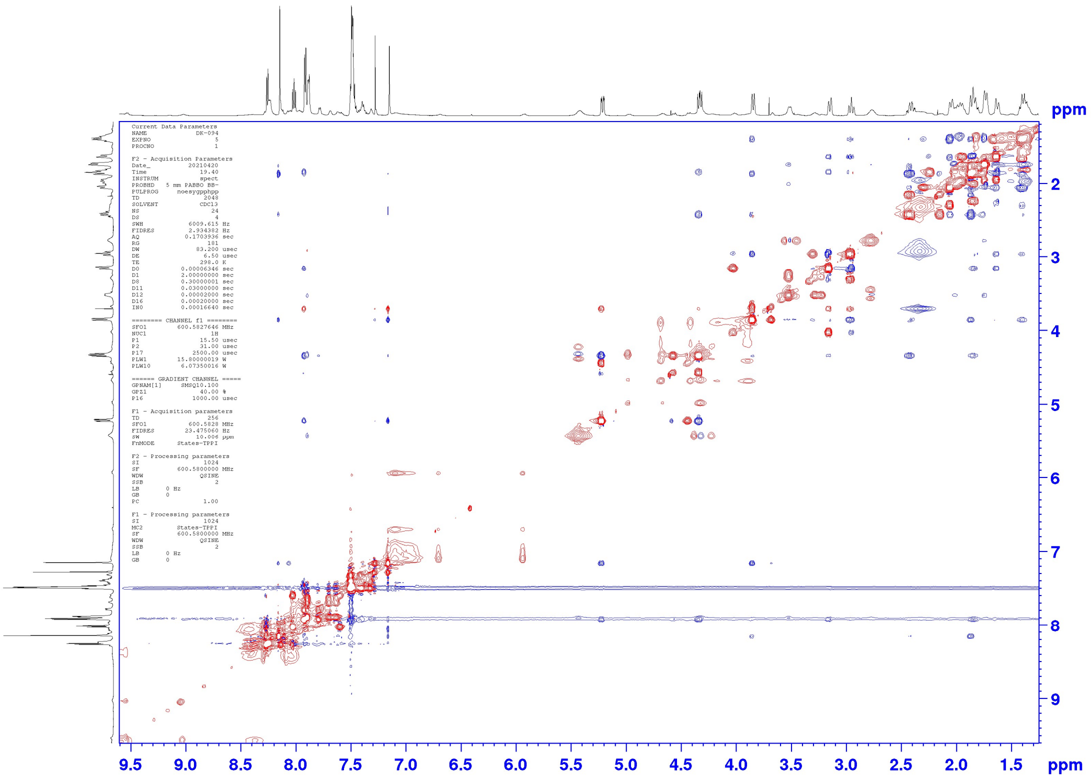

Figure S45. ^{1}H NOESY/EXSY experiment (600 MHz, $CDCl_3$, TMS) for 11-chloroderivatives obtained after thionyl chloride treatment of 13-benzyl-mefloquine. Positive phase correlations are drawn in red (diagonal and EXSY) and negative phase correlations are drawn in blue (NOE). Considered equilibrating species include a series of rotamers/conformers and epimers at nitrogen atom shown below:

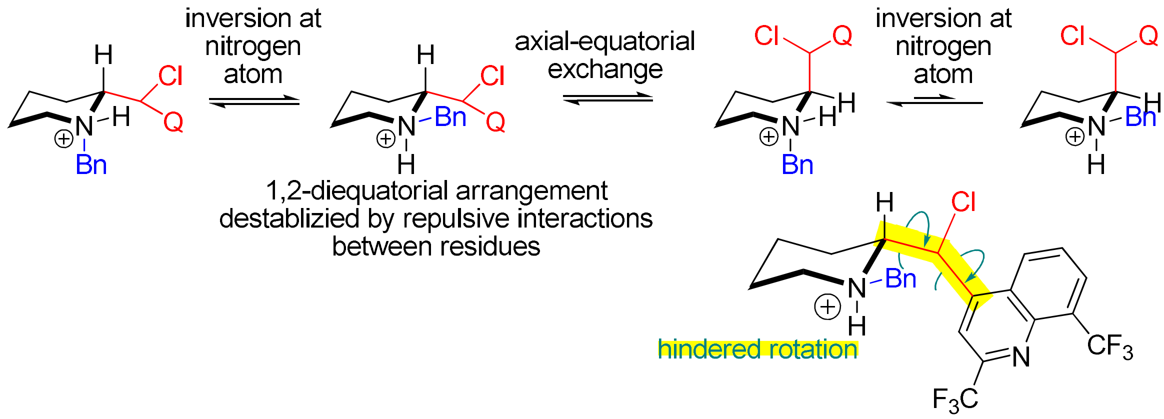

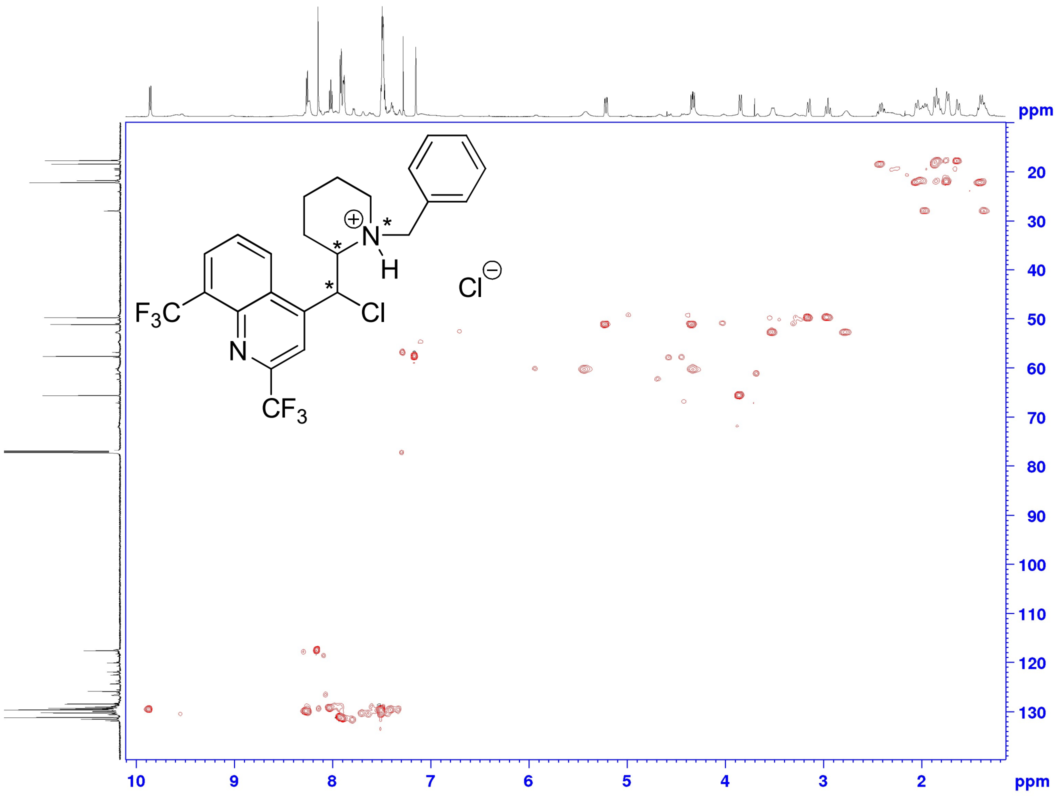

Figure S46. $^{1}H,^{13}C$ HSQC NMR experiment (600/151 MHz, $CDCl_3$, TMS) for 11-chloroderivatives obtained after thionyl chloride treatment of 13-benzyl-mefloquine

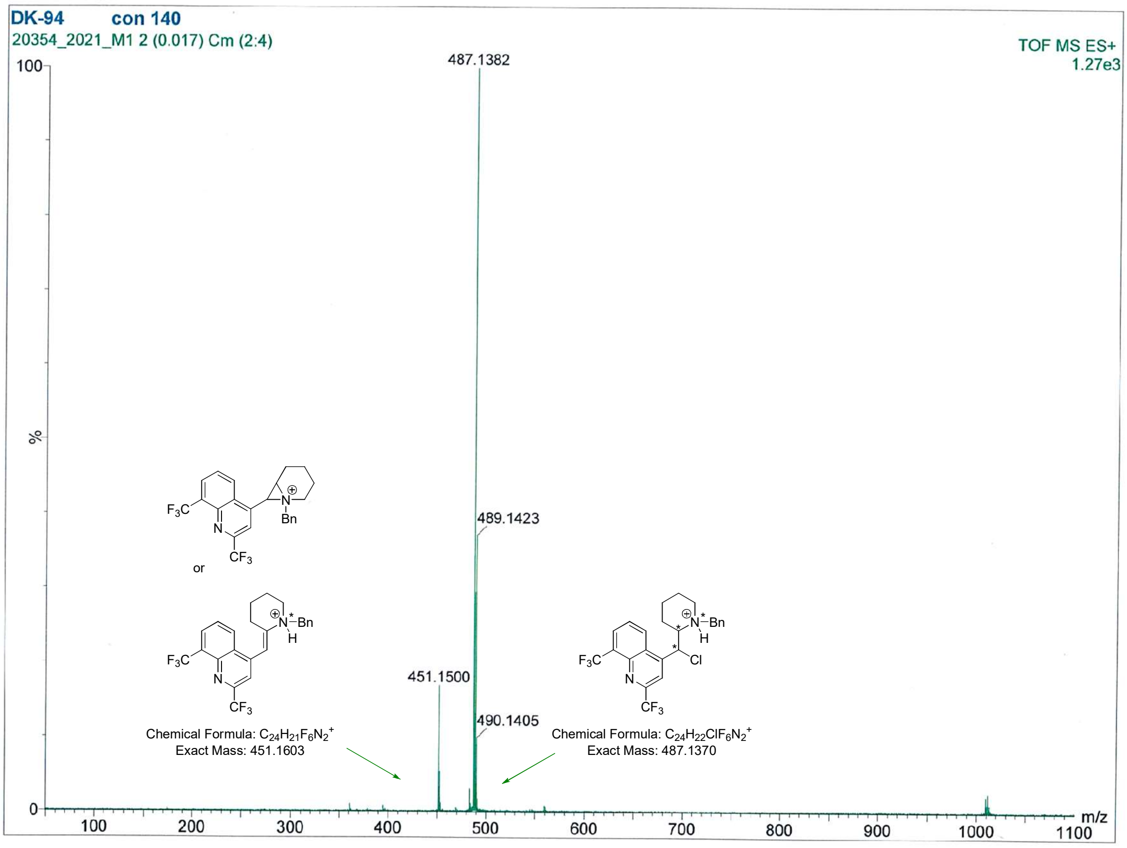

Figure S47. HRMS (ESI-TOF) for 11-chloroderivatives obtained after thionyl chloride treatment of 13-benzyl-mefloquine, plausible structures and their calculated *m*/*z*.

Chiral HPLC chromatogram plots

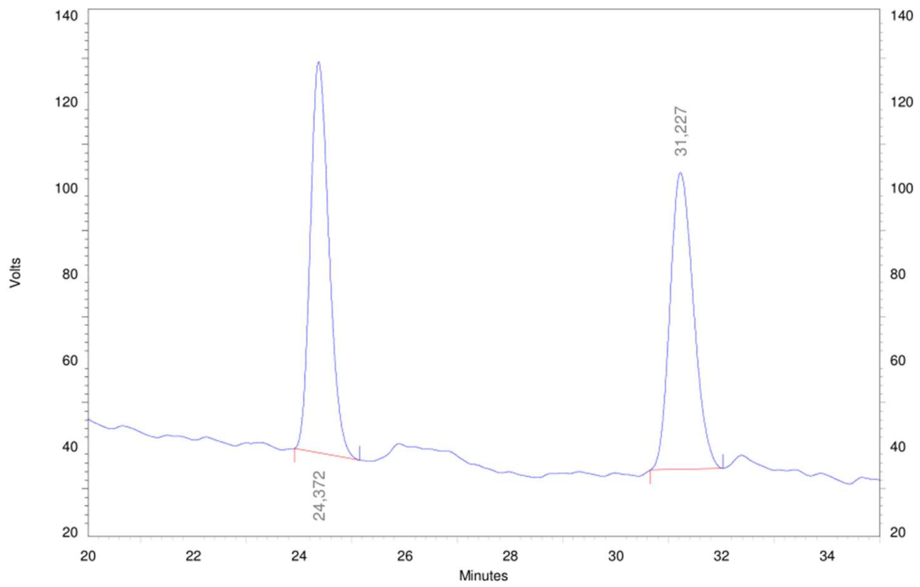

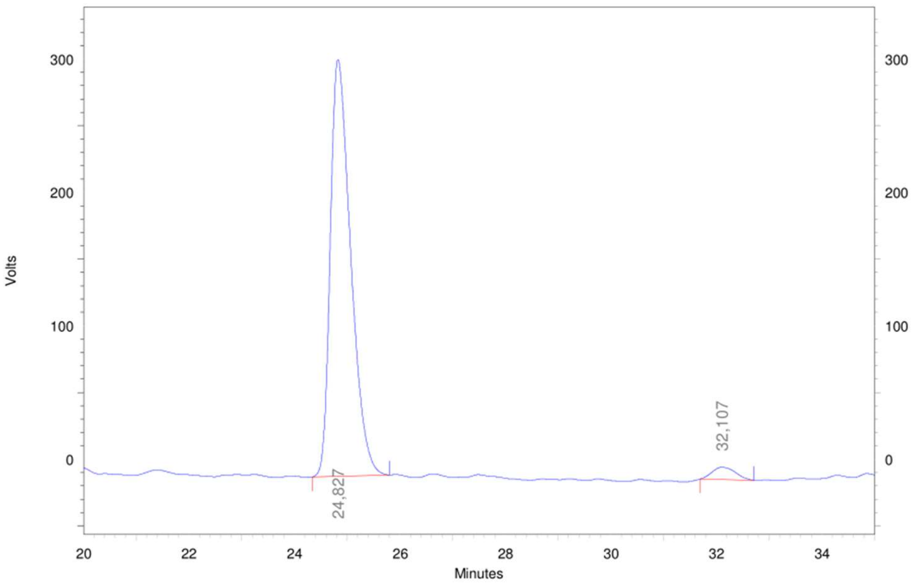

UV2000-220nm
Results (System
(2021-04-19
14:22:26)
(Reprocessed))

Retention Time	Area	Area %	Height	Height %
24,372	2231613	51,11	90854	56,86
31,227	2134929	48,89	68924	43,14
Totals	4366542	100,00	159778	100,00

UV2000-220nm
Results (System
(2021-04-27
12:47:59)
(Reprocessed))

Retention Time	Area	Area %	Height	Height %
24,827	8393829	96,74	312361	97,13
32,107	282936	3,26	9232	2,87
Totals	8676765	100,00	321593	100,00

Figure S48. HPLC chromatograms (IC-3 4.6×250 mm column, *n*-hexane : 2-propanol, 6:4, flow rate 0.8 mL/min, 220 nm) and integration for samples of 3-nitromethyl-cyclohexanone obtained with *erythro*-11-aminomefloquine catalysts: *racemic* (±)-**4** (left) and (+)-**4** of over 99%ee (right). The result of enantiomerically pure catalyst corresponds to Table 1 entry 1.

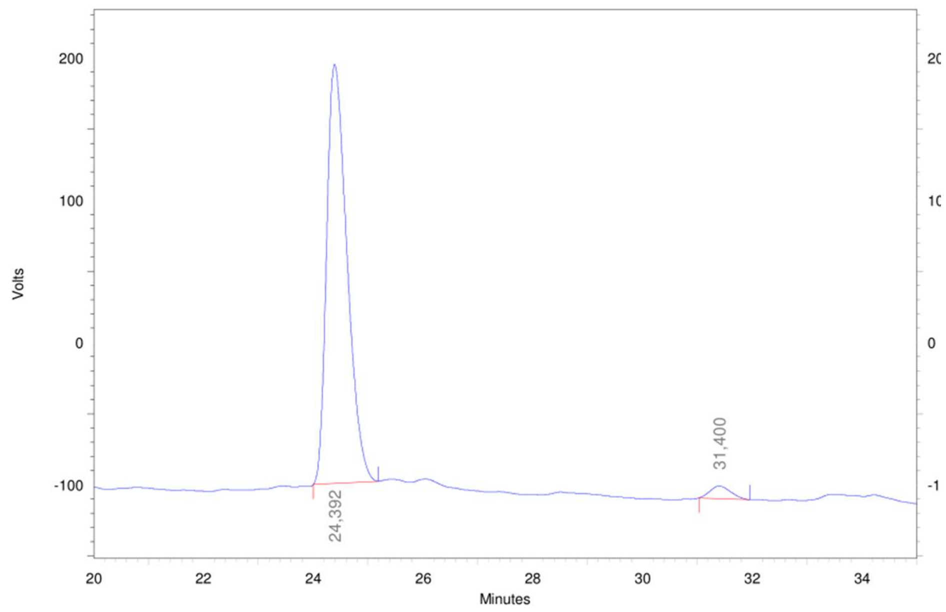

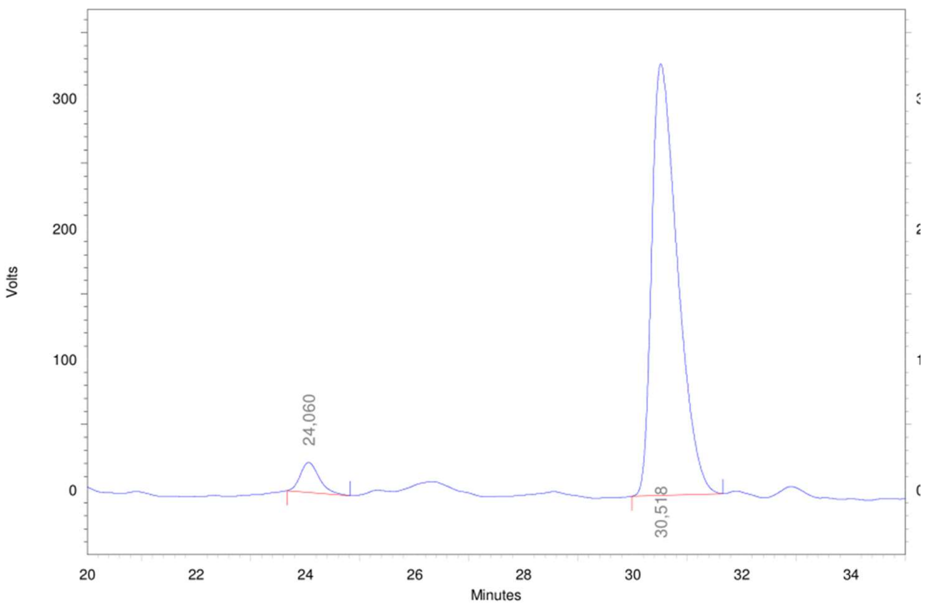

UV2000-220nm
Results (System
(2021-05-12
09:28:29)
(Reprocessed))

Retention Time	Area	Area %	Height	Height %
24,392	7581988	97,08	294437	97,10
31,400	228426	2,92	8785	2,90
Totals	7810414	100,00	303222	100,00

UV2000-220nm
Results (System
(2021-05-12
09:59:40)
(Reprocessed))

Retention Time	Area	Area %	Height	Height %
24,060	549989	4,82	22845	6,46
30,518	10860726	95,18	330580	93,54
Totals	11410715	100,00	353425	100,00

Figure S49. HPLC chromatograms (IC-3 4.6×250 mm column, hexane : 2-propanol, 6:4, flow rate 0.8 mL/min, 220 nm) and integration for sample of 3-nitromethyl-cyclohexanone obtained with catalyst (+)-**4** of 98%ee (left) and catalyst (–)-**4** of 98%ee (right). .

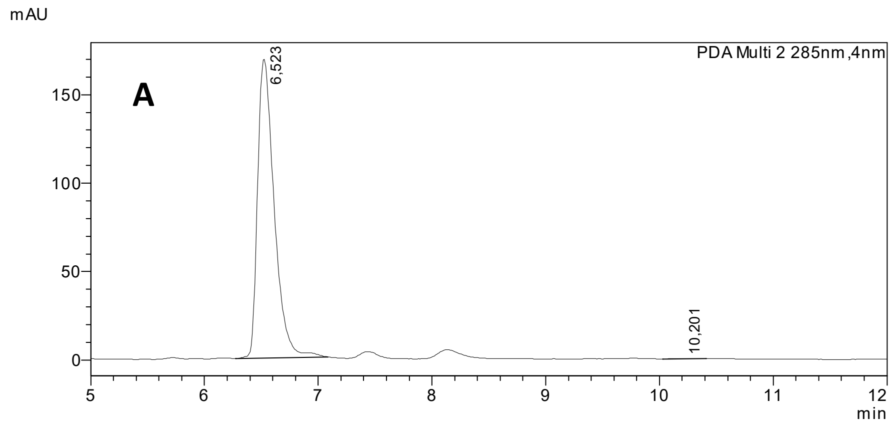

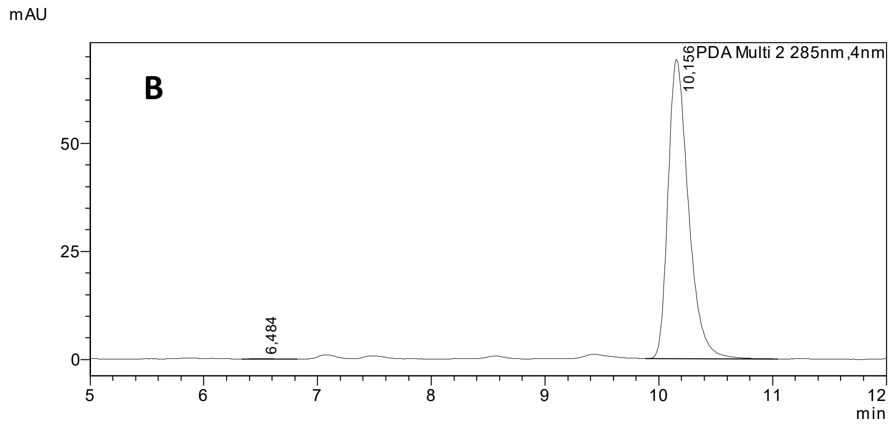

Peak#	Ret. Time	Area	Area%	Height	Height%	Resolution(USP)	Tailing F.
1	6,523	1714215	99,868	168744	99,856	--	1,565
2	10,201	2261	0,132	243	0,144	13,672	1,058
Total		1716476	100,000	168986	100,000		

Peak#	Ret. Time	Area	Area%	Height	Height%	Resolution(USP)	Tailing F.
1	6,484	1206	0,139	118	0,170	--	1,324
2	10,156	866406	99,861	69172	99,830	11,674	1,390
Total		867612	100,000	69290	100,000		

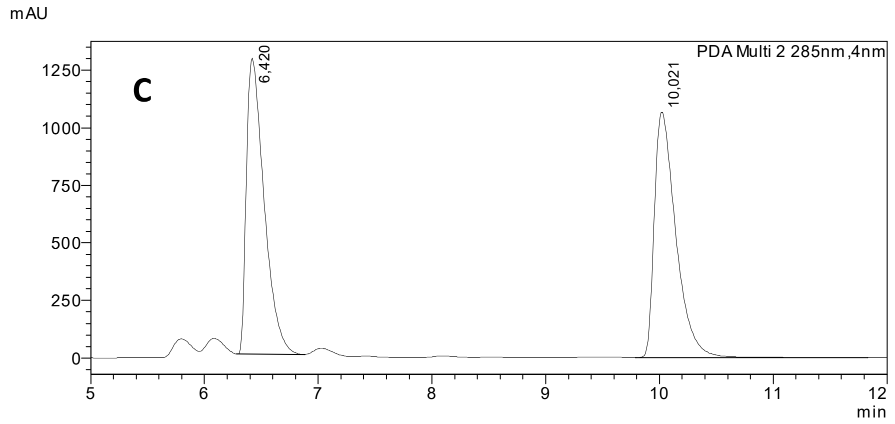

Peak#	Ret. Time	Area	Area%	Height	Height%	Resolution(USP)	Tailing F.
1	6,420	13995675	49,778	1285257	54,648	--	1,941
2	10,021	14120657	50,222	1066606	45,352	11,160	1,790
Total		28116332	100,000	2351862	100,000		

Figure S50. HPLC chromatograms (IA-3 4.6×250 mm column, hexane : 2-propanol, 9:1, flow rate 1 mL/min, 285 nm) and integration for samples of 11,13-diacetyl derivatives obtained from separated (+)-mefloquine (Panel **A**), (−)-mefloquine (Panel **B**) and racemic mefloquine (Panel **C**).

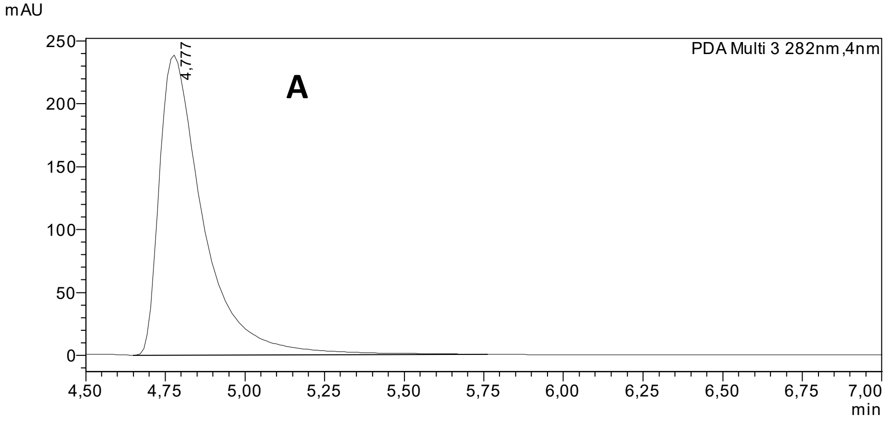

Peak#	Ret. Time	Area	Area%	Height	Height%	Resolution(USP)	Tailing F.
1	4,777	2279962	100,000	238368	100,000	--	2,115
Total		2279962	100,000	238368	100,000		

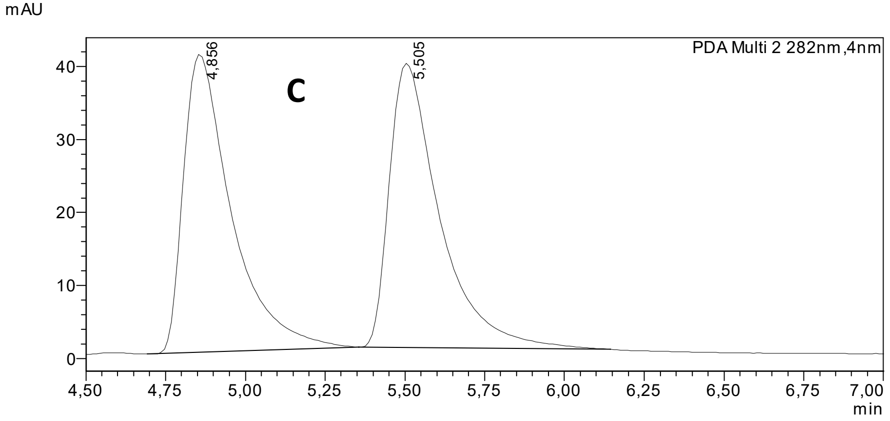

Peak#	Ret. Time	Area	Area%	Height	Height%	Resolution(USP)	Tailing F.
1	4,856	426983	49,970	40796	51,153	--	2,091
2	5,505	427498	50,030	38957	48,847	2,351	1,985
Total		854482	100,000	79753	100,000		

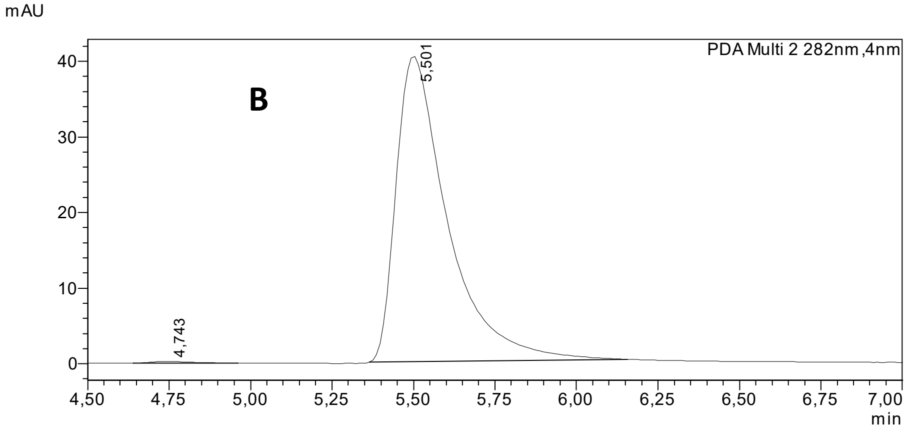

Peak#	Ret. Time	Area	Area%	Height	Height%	Resolution(USP)	Tailing F.
1	4,743	2292	0,496	232	0,569	--	1,438
2	5,501	460129	99,504	40536	99,431	2,853	2,104
Total		462420	100,000	40768	100,000		

Figure S51. HPLC chromatograms (IA-3 4.6×250 mm column, hexane : 2-propanol, 9:1, flow rate 1 mL/min, 282 nm) and integration for sample of (−)-(11*R*,12*S*)-**3** obtained from (−)-*erythro*-mefloquine (Panel **A**), (+)-(11*S*,12*R*)-**3** obtained from (+)-*erythro*-mefloquine (Panel **B**), and racemic **3**·HN_3 (Panel **C**).

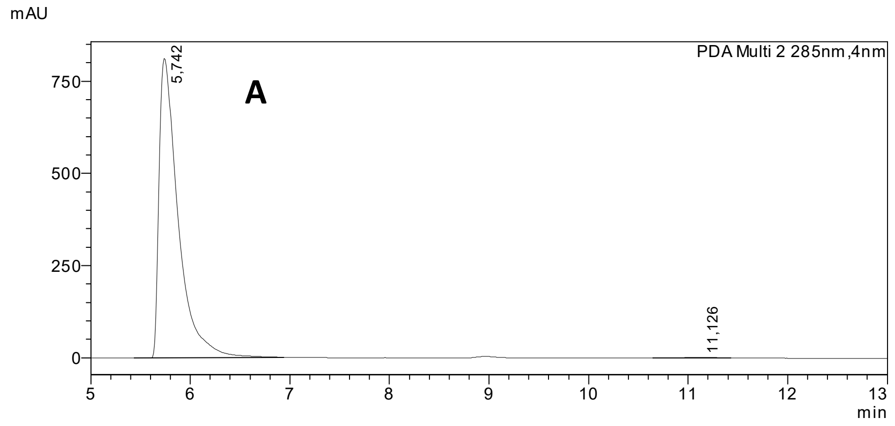

Peak#	Ret. Time	Area	Area%	Height	Height%	Resolution(USP)	Tailing F.
1	5,742	10763679	99,672	811421	99,811	--	2,598
2	11,126	35380	0,328	1537	0,189	11,106	0,836
Total		10799059	100,000	812958	100,000		

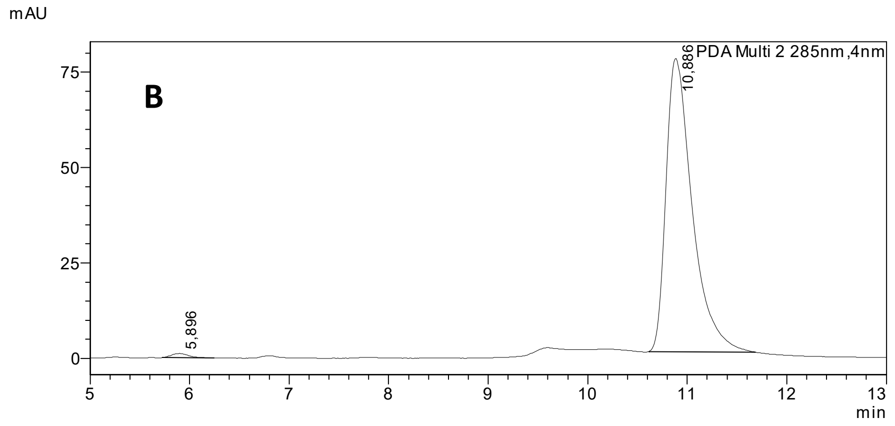

Peak#	Ret. Time	Area	Area%	Height	Height%	Resolution(USP)	Tailing F.
1	5,896	13627	0,960	1112	1,428	--	1,333
2	10,886	1406021	99,040	76786	98,572	12,516	1,673
Total		1419647	100,000	77899	100,000		

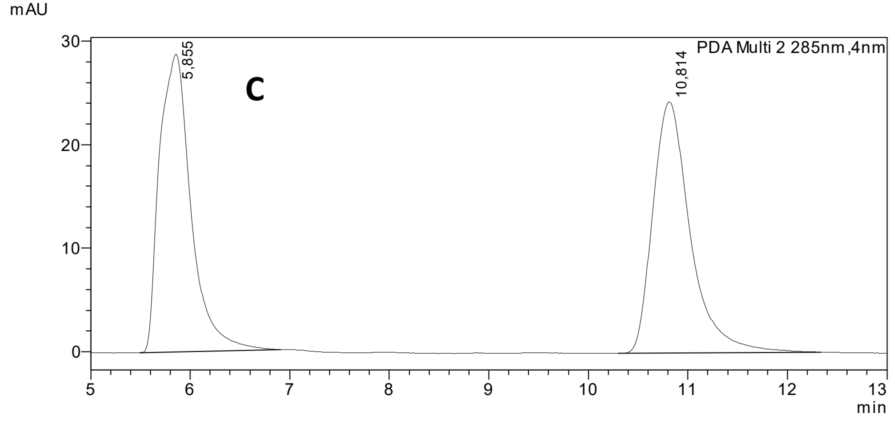

Peak#	Ret. Time	Area	Area%	Height	Height%	Resolution(USP)	Tailing F.
1	5,855	634497	49,915	28755	54,222	--	1,371
2	10,814	636658	50,085	24277	45,778	8,375	1,452
Total		1271155	100,000	53032	100,000		

Figure S52. HPLC chromatograms (IA-3 4.6×250 mm column, hexane : 2-propanol, 9:1, flow rate 1 mL/min, 285 nm) and integration for samples of 11,13-diacetyl derivatives obtained from (+)-(11*S*,12*R*)-11-aminomefloquine (Panel **A**), (−)-(11*R*,12*S*)-11-aminomefloquine (Panel **B**), and racemic *erythro*-11-aminomefloquine (Panel **C**).

Scheme for resolution of mefloquine (±-1)

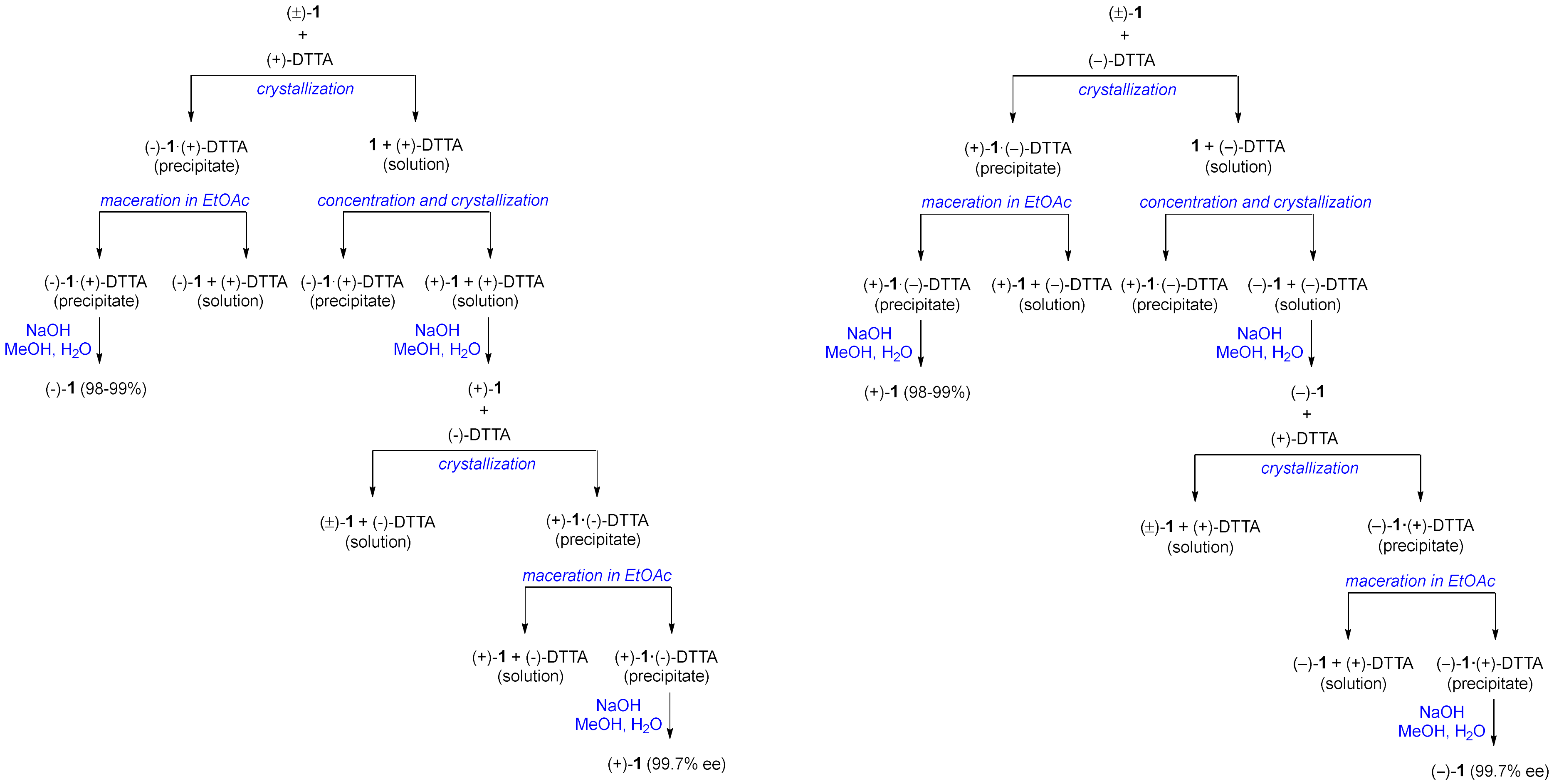

Scheme S1. Resolution scheme of *erythro*-mefloquine (**1**) with *O*,*O*-ditoluyltartaric acids (DTTA)

Computational data listings for compounds 14 and 15

Computations performed using Gaussian 16 code [S1]

Compound 14 (DFT/B3LYP/cc-PVDZ)

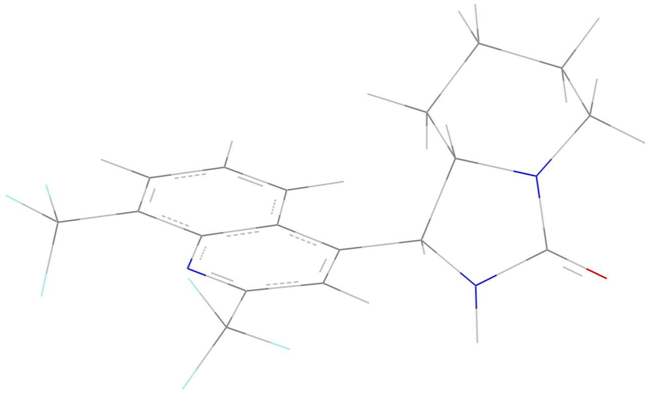

```
C  1.94728300 -0.45333100  1.10443800
H  1.89044500 -1.20233500  1.91634100
N  2.78629900  0.67052600  1.50271400
H  2.62348700  1.11148900  2.40327700
O  5.09021400  1.03439300  1.61339900
C  4.13239500  0.40020800  1.21407500
N  4.15368500 -0.71545900  0.39143800
C  2.80583600 -1.07427600 -0.04937700
H  2.69703600 -2.17303100 -0.04817300
C  2.53203500 -0.55224800 -1.46875000
H  1.56034000 -0.92768300 -1.82825800
H  2.46873200  0.54919600 -1.44685600
C  3.66370300 -0.98279100 -2.41661200
H  3.47007600 -0.59525400 -3.42964400
H  3.67212400 -2.08646700 -2.50099600
C  5.02952400 -0.49725400 -1.90904000
H  5.83809100 -0.84570700 -2.57301300
H  5.05595900  0.60692000 -1.91954500
C  5.29010400 -0.99024400 -0.47916800
H  6.16211600 -0.49754500 -0.02755300
H  5.47481500 -2.07987300 -0.48112000
C  0.53430100 -0.05406800  0.72208900
C -0.50898700 -1.02976600  0.62173900
C  0.21876000  1.26548000  0.46273700
C -0.31700600 -2.41875600  0.87190600
C -1.81770400 -0.57074700  0.24818800
C -1.10694100  1.59910900  0.10391900
H  0.98416000  2.03548500  0.53568200
C -1.36279700 -3.30707300  0.75490200
H  0.66700100 -2.78796600  1.16161000
C -2.88382500 -1.52025500  0.13476300
C -2.65210900 -2.85656000  0.38336900
H -1.20273300 -4.36876300  0.95123900
H -3.47134000 -3.56906100  0.29437700
N -2.09246000  0.73575500 -0.00518800
C -1.48384700  3.04407400 -0.19114700
C -4.27031600 -1.05512500 -0.25951600
F -4.28619100 -0.47814600 -1.47592300
F -5.13514500 -2.10090400 -0.31240800
F -4.78122300 -0.17442700  0.62101900
F -2.44916900  3.47952900  0.63795800
F -1.93274700  3.18635500 -1.45149700
F -0.42076300  3.86997300 -0.04452900
 SCF                                          -1533.623191 (Hartree/Particle)
 Sum of electronic and zero-point Energies=           -1533.305425
 Sum of electronic and thermal Energies=              -1533.282701
 Sum of electronic and thermal Enthalpies=            -1533.281756
 Sum of electronic and thermal Free Energies=         -1533.360348
 No of imaginary frequencies:   0
 Lowest frequencies:            9.3443, 22.4641, 33.6579 cm-1
```

[S1] Gaussian 16, Revision C.01, Frisch, M. J.; Trucks, G. W.; Schlegel, H. B.; Scuseria, G. E.; Robb, M. A.; Cheeseman, J. R.; Scalmani, G.; Barone, V.; Petersson, G. A.; Nakatsuji, H.; Li, X.; Caricato, M.; Marenich, A. V.; Bloino, J.; Janesko, B. G.; Gomperts, R.; Mennucci, B.; Hratchian, H. P.; Ortiz, J. V.; Izmaylov, A. F.; Sonnenberg, J. L.; Williams-Young, D.; Ding, F.; Lipparini, F.; Egidi, F.; Goings, J.; Peng, B.; Petrone, A.; Henderson, T.; Ranasinghe, D.; Zakrzewski, V. G.; Gao, J.; Rega, N.; Zheng, G.; Liang, W.; Hada, M.; Ehara, M.; Toyota, K.; Fukuda, R.; Hasegawa, J.; Ishida, M.; Nakajima, T.; Honda, Y.; Kitao, O.; Nakai, H.; Vreven, T.; Throssell, K.; Montgomery, J. A., Jr.; Peralta, J. E.; Ogliaro, F.; Bearpark, M. J.; Heyd, J. J.; Brothers, E. N.; Kudin, K. N.; Staroverov, V. N.; Keith, T. A.; Kobayashi, R.; Normand, J.; Raghavachari, K.; Rendell, A. P.; Burant, J. C.; Iyengar, S. S.; Tomasi, J.; Cossi, M.; Millam, J. M.; Klene, M.; Adamo, C.; Cammi, R.; Ochterski, J. W.; Martin, R. L.; Morokuma, K.; Farkas, O.; Foresman, J. B.; Fox, D. J. Gaussian, Inc., Wallingford CT, 2016.

Compound 15 (DFT/B3LYP/cc-PVDZ)

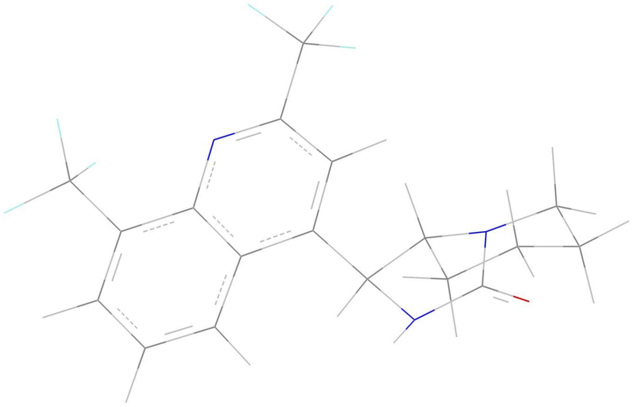

```
C  1.80450854 -1.26976618  0.05920434
H  1.69599369 -2.25866832  0.52545819
N  2.35157124 -1.40619671 -1.29422871
H  1.74228796 -1.65061032 -2.06830871
O  3.80344404 -0.16856361 -2.63553313
C  3.30582653 -0.41813354 -1.55445470
N  3.58405017  0.20833533 -0.34301103
C  2.96477659 -0.51466335  0.76783429
H  2.57083892  0.20106562  1.50967490
C  3.97400424 -1.44633001  1.45888246
H  3.51527742 -1.88331958  2.36219319
H  4.21586685 -2.27922692  0.77475968
C  5.25859896 -0.68147069  1.81425726
H  5.98375896 -1.36250074  2.28816227
H  5.02550189  0.09809920  2.56438882
C  5.87264882 -0.02661714  0.56764732
H  6.76973868  0.55566786  0.83623050
H  6.19622939 -0.81064002 -0.14001219
C  4.85685152  0.88835220 -0.13030916
H  5.21115436  1.21446687 -1.11786596
H  4.67391052  1.78929760  0.48237301
C  0.45482356 -0.55713583  0.07057112
C -0.77351065 -1.29470848  0.04497975
C  0.38061092  0.82496511  0.05778439
C -0.84429982 -2.71844037  0.01456944
C -2.00215904 -0.54700461  0.03933131
C -0.88536818  1.44950248  0.04676222
H  1.28267283  1.43410872  0.04703171
C -2.05950159 -3.36471973 -0.00822335
H  0.07131548 -3.30963821 -0.00084261
C -3.24993778 -1.25087869  0.02346859
C -3.26883483 -2.62871665  0.00090607
H -2.09709971 -4.45524840 -0.03430792
H -4.22370504 -3.15286071 -0.01335893
N -2.03488850  0.81001216  0.04508120
C -0.99481089  2.96752205  0.04968733
C -4.55248467 -0.47784898  0.02694002
F -4.67697709  0.31550817 -1.05331909
F -5.61632433 -1.32199593  0.01610928
F -4.68243886  0.29411695  1.12243279
F -1.63087582  3.41035546  1.15023923
F -1.67178383  3.41529803 -1.02137141
F  0.22862849  3.54907395  0.02970014
SCF                                                 -1533.620958 (Hartree/Particle)
Sum of electronic and zero-point Energies=          -1533.303298
Sum of electronic and thermal Energies=             -1533.280474
Sum of electronic and thermal Enthalpies=           -1533.279530
Sum of electronic and thermal Free Energies=        -1533.358764
No of imaginary frequencies:   0
Lowest frequencies:            11.3276, 12.2058, 34.7804 cm-1
```
