# Supplementary figures and images for: Chiral Vicinal Diamines Derived from Mefloquine
Source: J Org Chem. 2021 Jul 27;86(15):10654–64. doi: 10.1021/acs.joc.1c01316 (PMC8389910; doi:10.1021/acs.joc.1c01316)

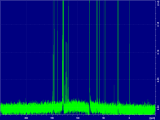

Supplement: Supplementary file 2 — jo1c01316_si_002.zip [file jo1c01316_si_002.zip › 10/13C/pdata/1/thumb.png]

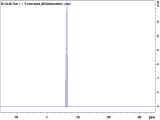

Supplement: Supplementary file 2 — jo1c01316_si_002.zip [file jo1c01316_si_002.zip › 10/19F/pdata/1/thumb.png]

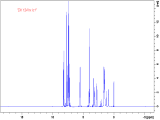

Supplement: Supplementary file 2 — jo1c01316_si_002.zip [file jo1c01316_si_002.zip › 10/1H/pdata/1/thumb.png]

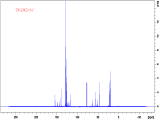

Supplement: Supplementary file 2 — jo1c01316_si_002.zip [file jo1c01316_si_002.zip › 11/13C/pdata/1/thumb.png]

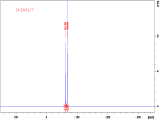

Supplement: Supplementary file 2 — jo1c01316_si_002.zip [file jo1c01316_si_002.zip › 11/19F/pdata/1/thumb.png]

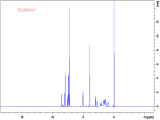

Supplement: Supplementary file 2 — jo1c01316_si_002.zip [file jo1c01316_si_002.zip › 11/1H/pdata/1/thumb.png]

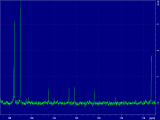

Supplement: Supplementary file 2 — jo1c01316_si_002.zip [file jo1c01316_si_002.zip › 12/13C/pdata/1/thumb.png]

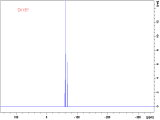

Supplement: Supplementary file 2 — jo1c01316_si_002.zip [file jo1c01316_si_002.zip › 12/19F/pdata/1/thumb.png]

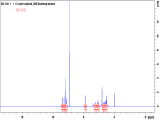

Supplement: Supplementary file 2 — jo1c01316_si_002.zip [file jo1c01316_si_002.zip › 12/1H/pdata/1/thumb.png]
